# Dibenzofuran Derivatives Inspired from Cercosporamide as Dual Inhibitors of Pim and CLK1 Kinases

**DOI:** 10.3390/molecules26216572

**Published:** 2021-10-30

**Authors:** Viet Hung Dao, Isabelle Ourliac-Garnier, Cédric Logé, Florence O. McCarthy, Stéphane Bach, Teresinha Gonçalves da Silva, Caroline Denevault-Sabourin, Jérôme Thiéfaine, Blandine Baratte, Thomas Robert, Fabrice Gouilleux, Marie Brachet-Botineau, Marc-Antoine Bazin, Pascal Marchand

**Affiliations:** 1 Cibles et Médicaments des Infections et du Cancer, IICiMed, EA 1155, Université de Nantes, 44000 Nantes, France; daoviethung@live.com (V.H.D.); isabelle.ourliac@univ-nantes.fr (I.O.-G.); cedric.loge@univ-nantes.fr (C.L.); jerome.thiefaine@univ-nantes.fr (J.T.); marc-antoine.bazin@univ-nantes.fr (M.-A.B.); 2 School of Chemistry, Analytical and Biological Chemistry Research Facility, University College Cork, Western Road, T12 K8AF Cork, Ireland; f.mccarthy@ucc.ie; 3 Sorbonne Université, CNRS, UMR8227, Integrative Biology of Marine Models Laboratory (LBI2M), Station Biologique de Roscoff, 29680 Roscoff, France; bach@sb-roscoff.fr (S.B.); baratte@sb-roscoff.fr (B.B.); thomas.robert@sb-roscoff.fr (T.R.); 4 Sorbonne Université, CNRS, FR2424, Plateforme de Criblage KISSf (Kinase Inhibitor Specialized Screening Facility), Station Biologique de Roscoff, 29680 Roscoff, France; 5 Centre of Excellence for Pharmaceutical Sciences, North-West University, Private Bag X6001, Potchefstroom 2520, South Africa; 6 Departamento de Antibióticos, Universidade Federal de Pernambuco, Recife 50670-901, PE, Brazil; teresinha.goncalves@ufpe.br; 7 EA GICC-ERL 7001 CNRS, Team IMT, University of Tours, 37200 Tours, France; caroline.denevault@univ-tours.fr; 8 CNRS ERL7001 LNOx «Leukemic Niche and redOx Metabolism», EA GICC, University of Tours, 37000 Tours, France; fabrice.gouilleux@univ-tours.fr (F.G.); marie.brachet.botineau@gmail.com (M.B.-B.)

**Keywords:** cercosporamide, dibenzo[*b*,*d*]furan, Pim kinases, CLK1 kinase, kinase inhibitors, anticancer agents

## Abstract

Pim kinases (proviral integration site for Moloney murine leukemia virus kinases) are overexpressed in various types of hematological malignancies and solid carcinomas, and promote cell proliferation and survival. Thus, Pim kinases are validated as targets for antitumor therapy. In this context, our combined efforts in natural product-inspired library generation and screening furnished very promising dibenzo[*b*,*d*]furan derivatives derived from cercosporamide. Among them, lead compound **44** was highlighted as a potent Pim-1/2 kinases inhibitor with an additional nanomolar IC_50_ value against CLK1 (cdc2-like kinases 1) and displayed a low micromolar anticancer potency towards the MV4-11 (AML) cell line, expressing high endogenous levels of Pim-1/2 kinases. The design, synthesis, structure–activity relationship, and docking studies are reported herein and supported by enzyme, cellular assays, and *Galleria mellonella* larvae testing for acute toxicity.

## 1. Introduction

**Figure 1:**
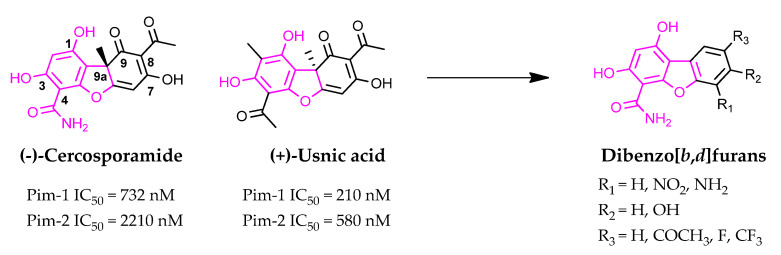
Drug design of the 1,3-dihydroxydibenzo[*b*,*d*]furan-4-carboxamide scaffold-based derivatives.

Cercosporamide is a natural product isolated from pathogen *fungus Cercosporidium henningsii*, commonly found in extensively cultivated plants (*Manihot esculenta*) for domestic consumption of tapioca in tropical and subtropical regions worldwide [1]. Moreover, this substance can be found in cultures of *Phoma* fungi isolated from Guinea plant *Saurauia scaberrinae* [2] and Chinese medicinal plant *Arisaema erubescensor* [3], or can be produced by fungi of the genus *Lachnum* and *Pseudaegerita* [4]. As the main structural features, (−)-cercosporamide exhibits a tricyclic unit containing an asymmetric quaternary carbon with absolute configuration *S* at position 9a (Figure 1). Additionally, cercosporamide presents two phenol groups at the position 1 and 3, a hydroxyl group (position 7), and a carboxamide group (position 4). An acetyl group (position 8) and an oxo group (position 9) are also attached to the dihydrodibenzofuran portion. Sugawara and co-workers [1] first identified this phytotoxin in 1991 from African strains of *C. henningsii*. In this work, cercosporamide was characterized as a tautomeric mixture (both in solution and in the solid state) through single-crystal X-ray diffraction, 2-D long-range ^1^H-^13^C, and COSY NMR techniques.

Since the discovery of cercosporamide, important findings concerning its biological activities have been reported. In 2004, Sussman et al. [5] reported cercosporamide as a potent selective inhibitor of *Candida albicans Ca*Pkc1 (PKC-like 1 kinase), which is central to fungal cell wall integrity and was validated as a fungal drug target in cases where drug resistance is evident. In addition, according to the literature [6], cercosporamide was shown to be a potent ATP-competitive *Ca*Pkc1 inhibitor with IC_50_ = 44 nM. In the last decade, nanomolar range inhibition of MAPK-interacting kinases (Mnk1/2) by cercosporamide has been shown to be an efficient alternative to block the activation of the translation initiation factor 4E (eIF4E) pathway and consequently to prevent tumor growth and progression [7,8]. In this way, activity against lung cancer [8], acute myeloid leukemia [9], and hepatocellular carcinoma [10] have been achieved by employing cercosporamide as an Mnk1/2 kinase inhibitor. In addition, inhibition of Mnk pathways by cercosporamide enhanced glioblastoma cell response to chemotherapy and radionuclide therapy [11]. Recent results in the zebrafish model, through the inhibition of bone morphogenetic protein receptor (BMPR) type I kinase, suggest that cercosporamide represents a potential therapeutic strategy to combat diseases with overactive BMPR signaling, including the rare genetic disorder fibrodysplasia ossificans progressiva (FOP) and the rare childhood brainstem tumor diffuse intrinsic pontine glioma (DIPG) [12].

In addition, cercosporamide-inspired synthetic compounds have also been reported. Furukawa et al. described the preparation of novel antihyperglycemic agents from cercosporamide [13]. In this work, a potent plasma glucose-lowering effect was observed in hyperglycemic KK/Ta mice. The same group completed the previous work by designing cercosporamide derivatives endowed with an antidiabetic effect and attenuated adverse effects as novel selective PPARγ modulators [14]. 

With the aim of developing new heterocyclic compounds displaying biological activities, our research group also validated cercosporamide as a valuable model in the drug design strategy. Indeed, benzofuran derivatives exhibited promising antiproliferative activity against two human non-small cell lung cancer (NSCLC) cell lines [15]. More recently, benzofuro[3,2-*d*]pyrimidines proved to be interesting agents in antifungal chemotherapy by restoring the susceptibility of resistant strains to azole treatment [16].

Although research on the biological activity of cercosporamide has increased in the last decade, few efforts have been carried out in the design of kinase inhibitor derivatives. The interest of the collaborators in this field of research [17,18,19,20,21,22] and the results of the literature highlighting cercosporamide as a kinase inhibitor and targeting human PKCα/β [4], Mnk1/2, Jak3, GSK3β, ALK4, and Pim-1 [7,8], from nanomolar to low micromolar ranges, prompted us to develop a new series of heterocyclic compounds inspired from cercosporamide (Figure 1).

The choice of polyoxygenated dibenzofurans was consistent with the literature precedent pointing out that such structures are well-established sources of biologically active scaffolds in drug discovery [23,24,25,26]. Otherwise, usnic acid, a secondary metabolite isolated from lichens, with structural similarity with cercosporamide was described as a Pim-1/2 inhibitor and active against myeloid leukemia [27,28]. Consequently, the hydroxy groups installed in positions 1 and 3 of the dibenzo[*b*,*d*]furan core structure will provide the structural requirements for a possible interaction with the ATP-binding site of the targeted kinase. In positions 7 (R_2_) and 8 (R_3_) of the molecule, additional hydroxyl and/or acetyl groups present in cercosporamide and usnic acid could also promote the binding of the designed compounds. Other modulations, including nitro, amino, fluoro, and trifluoromethyl groups, were also considered for the structure–activity relationship (SAR) study.

Taking into account the biological activity of the structural models used in the study, the main goal of the project was to obtain novel Pim-1/Pim-2 kinase inhibitors as anticancer agents. Indeed, the overexpression of serine/threonine Pim kinases (proviral integration site for Moloney murine leukemia virus kinases), in various types of hematological malignancies and solid carcinomas, is characterized in the literature, and these proteins promote cell proliferation and survival [29,30,31]. Consequently, Pim kinases were considered as a valuable target for antitumor therapy [32,33,34,35]. Interestingly, until now, dibenzofurans have not been described in the literature as Pim kinase inhibitors [21,29,30,31,34,36].

Eight new 1,3-dihydroxydibenzo[*b*,*d*]furan-4-carboxamide derivatives were screened against Pim-1 and Pim-2 and a panel of seven additional mammalian protein kinases to check their selectivity profile. The corresponding methoxy precursors were also evaluated for their kinase inhibition properties to complete the SAR study. Antiproliferative activities of the most promising kinase inhibitors were further tested against seven different cell lines. In complement, the determination of in vivo toxicity using *Galleria mellonella* larvae was performed. Finally, information of ligand–protein interactions was provided through docking study on Pim-1.

## 2. Results and Discussion

### 2.1. Synthesis

**Figure 2:**
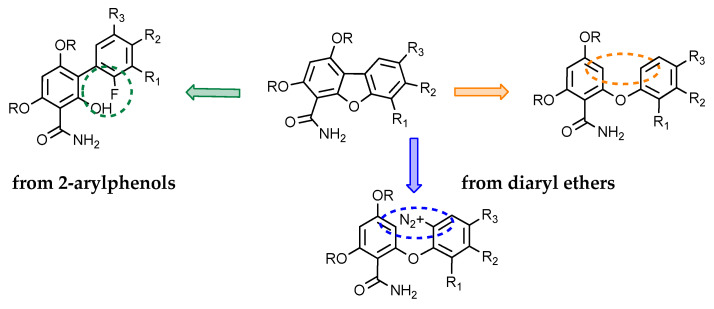
Proposed synthetic strategies to obtain the dibenzo[*b*,*d*]furan derivatives.

The synthetic strategies to prepare dibenzofurans can be classified into two main categories [37,38,39,40,41] (Figure 2). One involves the construction of the dibenzofuran from the ring closure of diaryl ethers through C−C bond formation [37,38]. The second approach refers to the intramolecular *O*-arylation of 2-arylphenols according to a mechanism of etherification [40,41].

#### 2.1.1. Access to Dibenzofuran Derivatives by Intramolecular C-C Bond Formation from Diaryl Ethers

**Scheme 1:**
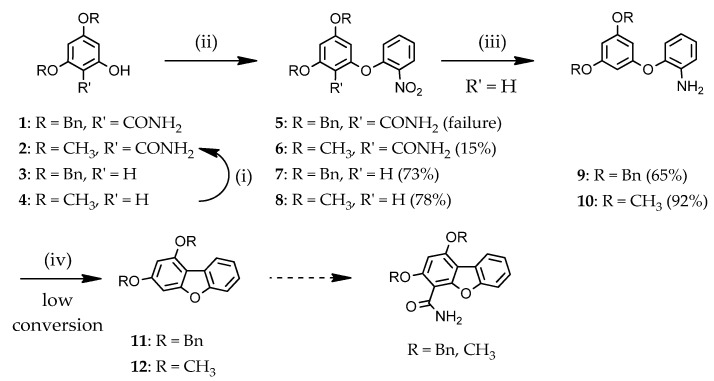
Reagents and conditions: (i) CSI, CH_3_CN, 0 °C, 10 min, then HCl 5M, rt, 10 h, 45% (ii) KOH, DMF, 120 °C, 30 min, then 1-iodo-2-nitrobenzene, Cu(0), 170 °C, 24 h (for **5** and **6**) and 2 h (for **7** and **8**); (iii) Zn dust, NH_4_Cl, CH_3_OH, 80 °C, 2 h; (iv) (1) NaNO_2_, H_2_SO_4_, 0 °C, 45 min, (2) Cu(0), H_2_SO_4_, 60 °C, 24 h, 6% (for **11**) and 11% (for **12**) of conversion rates or Pd(OAc)_2_, EtOH, 60 °C, 24 h, 8% (for **11**), and 28% (for **12**) of conversion rates.

The first method involves *ortho*-(aryloxy)aryldiazonium salts as intermediates to form an intramolecular C-C bond via a free-radical cyclization (Figure 2) [42,43,44]. Before obtaining the diazonium salt, the advantage of this approach relates to the nitro function that promotes the access to diaryl ethers **5**–**8** during the initial SN_Ar_ reaction between 1-iodo-2-nitrobenzene and phenolic derivatives **1**–**4** (Scheme 1).

The standard copper-catalyzed Ullmann diaryl ether synthesis [45,46] was first performed in the presence of the previously synthesized phenol derivative **1** [16] and its dimethyl analogue **2**, obtained in the same conditions from commercially available 3,5-dimethoxyphenol **4**. This route could allow the introduction of the carboxamide function before the heterocyclization step. Unfortunately, no reaction occurred for the dibenzylated phenol **1**, and its dimethyl counterpart **2** gave only a poor yield of 15% after the one-day reaction (Scheme 1). Modification of the combination of the copper source (CuI), base (Cs_2_CO_3_, NaH), and additional ligand (*N*,*N*-dimethylglycine) did not improve the completion of the reaction. Interestingly, the same reaction carried out from phenols **3** [16] and **4**, without carboxamide substitution, afforded the desired diaryl ethers **7** and **8** in good yields (73% and 78%, respectively). This result indicated that the reaction is more sensitive to the electronic-withdrawing effect of the carboxamide and the steric hindrance in the *ortho* position of the phenol than to the bulky benzyl-protecting groups.

Nitro-diaryl ethers **7** and **8** were further reduced into the corresponding anilines **9** and **10** in a moderate to good yield, respectively (Scheme 1). In the presence of zinc dust and ammonium chloride [47] in methanol, for better solubility, it was necessary to warm the medium to 80 °C to achieve a complete conversion of the hydroxylamine intermediate to the amino group. Cyclization of the compounds **9** and **10** proceeded after transformation of the amino function into the corresponding diazonium salts, which were then heated with copper powder [48] or palladium acetate [37] to form the dibenzo[*b*,*d*]furans **11** and **12**. Unfortunately, the conversion rates, measured by UPLC-MS analysis, to the target compounds remained very low (6 to 28%), limiting the application of this route further. The major products of the reaction were the corresponding diaryl ethers with loss of the amino group.

**Scheme 2:**
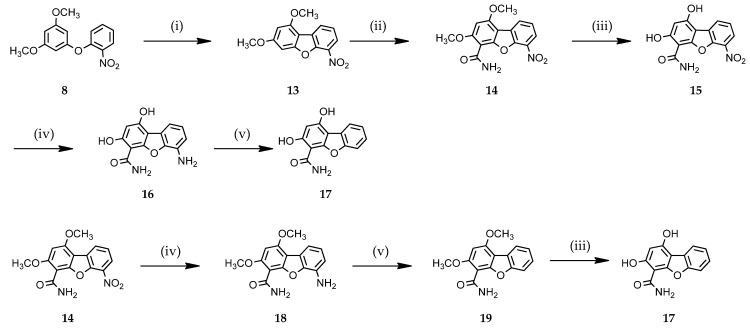
Reagents and conditions: (i) Pd(OAc)_2_, AcOAg, PivOH, 130 °C, 4 h, 72%; (ii) CSI, CH_3_CN, rt, 12 h, then HCl 5M, rt, 6 h, 53%; (iii) Pyridine.HCl, 200 °C, MW, 15 min, 51% (for **15**) and 48% (for **17**); (iv) Zn dust, NH_4_Cl, CH_3_OH, 80 °C, 2 h, 62% (for **16**) and 49% (for **18**); (v) NaNO_2_, H_2_SO_4_, EtOH, 0 °C, 30 min, then 80 °C, 45 min, 52% (for **17**) and 46% (for **19**).

However, this synthetic route enabled us to obtain a very useful intermediate compound **8** to ensure the continuation of the project. Indeed, taking into account our literature survey, an intramolecular palladium(II)-catalyzed oxidative carbon-carbon bond formation, according to Fagnou’s conditions [49,50], would allow us to access a nitrodibenzofuran derivative **13** whose nitro function is easily replaced by a hydrogen via the reduction of the corresponding diazonium salt (Scheme 2). Additionally, this approach has the advantage of functionalizing the dibenzofuran scaffold with a nitro group, providing functional diversity, after reduction to the corresponding amine for instance.

Following Pd-mediated CH activation conditions, the dibenzofuran ring closure was performed, from nitro-diaryl ether **8**, in the presence of silver acetate (AcOAg) in pivalic acid as solvent at 130 °C with a 72% yield. Afterwards, a direct aminocarbonylation at the C-4 position of the dibenzofuran derivative **14**, using chlorosulfonyl isocyanate (CSI) as an electrophilic reagent, led to compound **14** after acidic hydrolysis of the corresponding *N*-chlorosulfonylcarboxamide intermediate [15,16,51]. Demethylation of the methoxy groups was then carried out by heating compound **14** in neat pyridine hydrochloride at 200 °C, under microwave irradiation (MW), to lead in a short time (15 min) to the dihydroxy derivative **15**, available for biological testing [52]. The nitro function was then reduced by using zinc and ammonium chloride, as described above, to afford the final amino compound **16**. Finally, deamination of dibenzofuran derivative **16**, via the thermal decomposition of the corresponding diazonium salt with the release of nitrogen, furnished the unsubstituted target compound **17**. To scope the synthetic approach further, the nitro derivative **14** was reduced to afford **18** as a precursor of the dimethoxy derivative **19,** which could give compound **17** by an alternative route, after demethylation reaction. By applying such a three-step procedure from compound **14**, the global yield decreased by comparison to the initial strategy (11% vs. 16.5%) but the dimethoxy precursors **14**, **18**, and **19** of the dihydroxy-dibenzofurans **15**, **16**, and **17**, respectively, were obtained for complete SAR study.

**Scheme 3:**
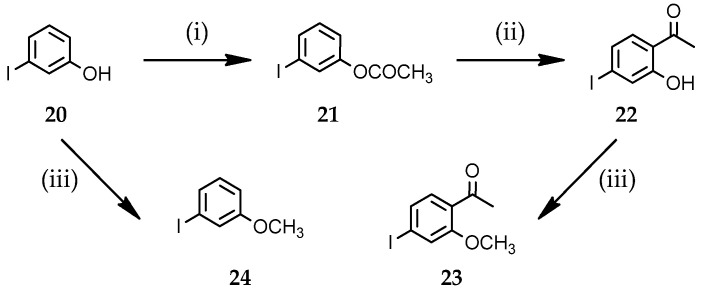
Reagents and conditions: (i) Ac_2_O, Pyridine, 130 °C, 2 h, 90%; (ii) BF_3_.2CH_3_COOH, 180 °C, 12 h, 88%; (iii) CH_3_I, Cs_2_CO_3_, CH_3_CN, 70 °C, 2 h, 91% (for **23**) and 4 h, 89% (for **24**).

**Scheme 4:**
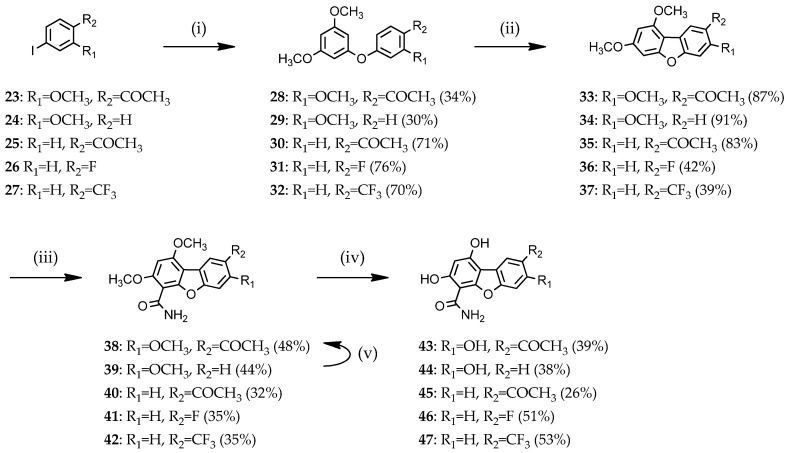
Reagents and conditions: (i) 3,5-Dimethoxyphenol **4**, Cs_2_CO_3_, CuI, DMF, 110 °C, MW, 1 h; (ii) Pd(OAc)_2_, AgOAc, PivOH, 130 °C, 4–24 h; (iii) CSI, CH_3_CN, 0 °C-rt, 6–12 h, then HCl 5M, rt, 6–12 h; (iv) Pyridine.HCl, 200 °C, MW, 15 min; (v) AcCl, AlCl_3_, Cl-CH_2_CH_2_Cl, rt, 1 h, 74%.

According to Liégault et al. [49], the *ortho*-directed CH activation of diaryl ethers, discussed above for accessing dibenzofurans, tolerates a broad substrate scope including both electron-rich and electron-deficient derivatives. Consequently, this approach was envisaged for pharmacomodulation of R_1_, R_2_, and R_3_ of the additional target compounds (Figure 1). The first step consisted of the synthesis of the suitable aryl iodide derivatives (Scheme 3) for the preparation of starting diaryl ethers (Scheme 4). To this end, the acetylation of 3-iodophenol **20** was readily achieved with acetic anhydride in pyridine [15] to offer phenyl acetate derivative **21**, which was engaged in a Fries rearrangement reaction in the presence of boron trifluoride-acetic acid complex (BF_3_.2CH_3_COOH) to give 2-hydroxyacetophenone **22** in a good yield of 88% [53]. The protection of the phenol moiety was then carried out using iodomethane in basic conditions to obtain compound **23** in good yield (Scheme 4). The same protocol was employed to obtain 3-iodoanisole **24** from 3-iodophenol **20**.

Diaryl ethers **28**–**32** were furnished by Ullmann-type coupling between 3,5-dimethoxyphenol **4** and synthesized (**23**, **24**) or commercially available (**25**–**27**) aryl iodides (Scheme 4) [50]. It should be noted that the presence of a methoxy group on aryl halides has a deleterious effect for the reaction yield when comparing compounds **28**, **29** (30–34%), and **30**–**32** (70–76%). Otherwise, the addition of *N*,*N*-dimethylglycine ligand in the mixture, to stabilize the copper complex, or increased reaction time did not improve the yields. Afterwards, the general reaction sequence (Scheme 4) was the same as previously discussed: (1) Pd-catalyzed CH activation for the dibenzofuran ring closure, from moderate to good yields (compounds **33**–**37**); (2) introduction of the carbamoyl group, in low yields, through electrophilic attack of CSI and acidic hydrolysis (compounds **38**–**42**); and (3) demethylation using pyridinium hydrochloride, in poor yields (compounds **43**–**47**) due to difficulties in the purification on column chromatography. Interestingly, Friedel–Crafts acetylation performed on compound **39**, in the presence of acetyl chloride and aluminum trichloride in 1,2-dichloroethane, allowed isolation of compound **38** with a good yield of 74% (Scheme 4) [54]. The proton NMR data validated the regioselective SE_Ar_ at the position 8 of the heterocycle.

#### 2.1.2. Access to Dibenzofuran Derivatives by Intramolecular C-O Bond Formation from 2-Aryl Phenols

**Scheme 5:**
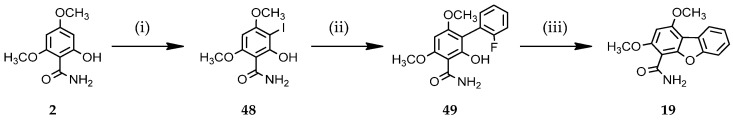
Reagents and conditions: (i) NIS, CH_2_Cl_2_, 0 °C, 10 min, 60%; (ii) 2-Fluorophenylboronic acid, Pd(OAc)_2_, PPh_3_, K_3_PO_4_, CH_3_CN/H_2_O (4/1) sealed tube, 90 °C, MW, 2 h, 26%; (iii) Cs_2_CO_3_, CH_3_CN, 150 °C, MW, 1 h, 61%.

In a final strategy for the access to the dibenzo[*b*,*d*]furan scaffold, we were interested in exploring the carbon-oxygen bond formation of 2-aryl phenols in our series [52]. In addition, it gave us the opportunity to confirm the regioselectivity of the carbamoyl introduction at the C4-position of the dibenzofuran ring by performing the reaction from benzamide derivative **2**, where the CONH_2_ group was already installed (Scheme 5).

From this perspective, mono-iodination of compound **2** was first realized in the presence of *N*-iodosuccinimide to afford *ortho*-iodophenol derivative **48** [16]. In the next step, we followed the strategy described by Jepsen et al. [52] consisting of the introduction of an *ortho*-fluorophenyl for subsequent intramolecular cyclisation. Thus, the palladium-catalyzed Suzuki–Miyaura coupling between 2-fluorophenylboronic acid and compound **48**, in the presence of palladium acetate, triphenylphosphine, and potassium phosphate as a base, provided the expected compound **49** [55]. Unfortunately, the yield remained very low (26%), but a preliminary attempt of C-C bond formation using Jepsen’s conditions under microwave irradiation (PdCl_2_(PPh_3_)_2_, K_2_CO_3_, DME/H_2_O, 80 °C, MW, 2 h) [52] proved to be unsuccessful with degradation of the reaction mixture.

Finally, the reaction of biaryl **49** with cesium carbonate at 150 °C in acetonitrile, under microwave irradiation, furnished compound **19**, involving a fluorine atom as the leaving group for nucleophilic substitution. The NMR data and physicochemical properties of the compound obtained after ring closure reaction exactly matched with the previously described dibenzofuran derivative **19** in Scheme 2, confirming the proposed regioselectivity of the introduction of the carbamoyl function by CSI reagent. Nevertheless, after analyzing the global results of this synthetic route, optimization work will be necessary regarding the Suzuki–Miyaura coupling to consider it as a valuable approach of our series. Furthermore, when comparing the yield to obtain compound **19** in this reaction sequence (4.3% in 4 steps from compound **4**) and the synthesis developed above (6.7% in 5 steps from compound **4**, see Scheme 1 and Scheme 2), the second approach is less favored.

### 2.2. Kinase Assays

The synthesized compounds were tested using a luminescence-based assay [56] against a panel of nine kinases: Pim-1, Pim-2, CDK5/p25, CDK9/CyclinT, Haspin, CK1ε, and GSK3β (from human); DYRK1A (from *Rattus norvegicus*); and CLK1 (from *Mus musculus*). The results are summarized in Table 1 and Table 2. All these compounds were considered inactive on Haspin, DYRK1A, GSK3β, CK1ε, CDK5/p25, and CDK9/CyclinT since they displayed IC_50_ values above 10 µM, except fluoro derivative **41** from series A: Haspin (0.93 µM) and DYRK1A (1.33 µM) and its dihydroxy counterpart **46** belonging to series B Haspin (0.80 µM), DYRK1A (0.69 µM), CK1 (0.68 µM), and CDK9 (0.92 µM), which were highlighted as less selective kinase inhibitors: (Table 1, entries 14 and 15). Globally, compounds bearing 1,3-dihydroxy groups in the benzamide portion (series B) displayed micromolar to submicromolar inhibition of both Pim-1 and CLK1 (with the exception of dihydroxydibenzofuran **47** (Table 1, entry 17) inactive on CLK1 kinase), in contrast with the results obtained for the 1,3-dimethoxy analogues (series A, with the exception of compounds **19** and **41** (Table 1, entries 6 and 14) displaying submicromolar IC_50_ values against CLK1 kinase) and irrespective of the substituents in R_1_, R_2_, or R_3_.

Small variations can be observed depending upon steric constraint or electronic effects. Indeed, the non-substituted compound **17** (Table 1, entry 7) showed significant IC_50_ values of 0.23 and 0.26 µM against Pim-1 and CLK1, 3.5-fold more active and comparable to those observed for the disubstituted compound **43** (Table 1, entry 9, IC_50_ values of 0.82 and 0.29 µM, respectively), which appeared the most structurally related to cercosporamide. Compound **44**, bearing a 7-OH substituent on the dibenzofuran ring (Table 1, entry 11), displayed the most potent inhibitory activities against both enzyme*s* with IC_50_ values of 0.06 (Pim-1) and 0.026 µM (CLK1) while 7-substitutions by a fluorine atom (compound **46**, Table 1, entry 15) or a trifluoromethyl group (compound **47**, Table 1, entry 17) reduced the inhibitory potency against Pim-1 (IC_50_ > 1 µM) and abrogated CLK1 inhibition for **47**. Furthermore, the 8-acetyldibenzofuran derivative **45** (Table 1, entry 13) exhibited an increased IC_50_ value of 0.28 µM against Pim-1 and also remained 5-fold less active than **44** against CLK1 with an IC_50_ value of 0.14 µM. However, by comparison with compound **43** possessing the 7-OH and 8-COCH_3_ substitution patterns of cercosporamide, compound **45** without hydroxyl has been identified as a better kinase inhibitor towards both discussed enzymes. All these promising compounds belonging to series B were also potent submicromolar Pim-2 inhibitors (Table 2), with IC_50_ values ranging from 0.035 (compound **44**) to 0.37 µM (compound **47**).

Taking into account the kinase inhibitory activity of cercosporamide (Table 1 and Table 2, entry 1), the development of dibenzofuran derivatives led us to obtain more potent compounds against Pim-1/2 or as active compounds as the reference molecule. In addition, the novel compounds gave rise to low micromolar and nanomolar CLK1 inhibition compared to cercosporamide, constituting a very interesting result due to the CLK1 implications in human disease including cancer [57].

### 2.3. Docking Studies

**Figure 3:**
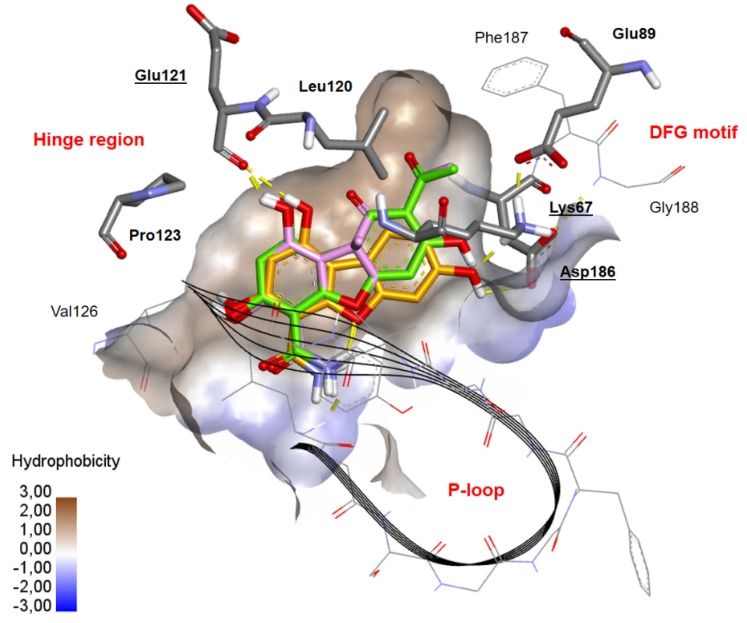
Binding pose found by the docking program GOLD for compound **44** (orange), compound **43** (green), and cercosporamide (pink) within the ATP pocket of Pim-1 (PDB ID 3A99). Hydrogen bonds are indicated as yellow lines.

Compounds **44**, **43**, and cercosporamide exhibit a similar binding mode when docked into the ATP pocket of Pim-1 (Figure 3). The 1-OH position in the benzamide portion forms a H-bond interaction with the carbonyl group of hinge residue Glu121 while the 7-OH position could favor an additional H-bond interaction with the ammonium side chain of conserved residue Lys67 and/or the carboxylate side chain of Asp186 (only visible for the most active compound **44**). Such a binding mode is consistent with the experimental data, where 1,3-dihydroxybenzamide series (series B) are more much active than their 1,3-dimethoxy analogues (series A). All the 3-OH are oriented towards the carbonyl group of hinge residue Pro123, but the distance appears to be too large (>4.0 Å) to enable the formation of an appropriate H-bond interaction. The 4-carboxamide group does not seem to interact with a particular residue in the ATP-pocket but instead performs an intramolecular H-bond with the furan ring. This contrasts with previous docking studies of cercosporamide in the ATP pocket of Mnk2 kinase that showed H-bond interaction between the 4-carboxamide of the phenyl portion and hinge residues Glu160 and Met162 [8]. However, other docking studies of usnic acid, another dibenzofuran analogue with the main structural difference being 4-acetyl and 2-methyl groups, proposed a different binding mode with Pim-1 kinase, which seems consistent with our hypothesis [27]. Of course, we cannot discard other possible binding modes for these dibenzo[*b*,*d*]furan inhibitors since they contain many H-bond donor and acceptor substituents that can interact depending upon the specific environment of each kinase. The information of the role of the amide function in the binding mode of the compounds is under study through the synthesis of the counterparts without amide function. In a rational approach, their binding pose will be compared to their inhibitory potency on Pim-1 to complete the study.

### 2.4. In Vitro Cell-Based Assays and Acute Toxicity Testing

The Pim-1/2 and CLK-1 inhibitors **15**–**17** and **43**–**46,** belonging to series B**,** were tested for their antiproliferative activity against six cancer cell lines, and L929 (mouse fibroblasts) for in vitro toxicity evaluation (Table 3, entries 1 to 7). Cercosporamide as a reference and compound **47**, being less active against Pim-1 and inactive on CLK1, were also considered in the anticancer screening (Table 3, entries 8 and 9). The commercially available compound **SGI-1776**, a small-molecule pan-Pim protein kinase inhibitor with IC_50_ values of 0.05 (Pim-1) and 0.10 µM (Pim-2), was used as a positive control for the in vitro studies. Various types of hematological malignancies were selected for in vitro testing: the human acute myeloid leukemia (AML) cell line MV4-11, and the human chronic myeloid leukemia (CML) cell lines KU812 and K562. Indeed, Pim kinases are overexpressed in a wide range of hematopoietic malignancies [27,28,29,30,31,32,33,34,35,36]. Solid tumors were also included in the panel of tested cell lines: MCF-7 (human breast adenocarcinoma), HT-29 (human colon adenocarcinoma), and HeLa (human cervical cancer) cells. Cytotoxic effects were evaluated using an MTT assay, and living cells were also counted with the trypan blue dye exclusion method for MV4-11, KU812, and K562 cells.

In a general trend, all the dibenzofuran derivatives, except **47** (Table 3, entry 8), displayed moderate to good antiproliferative activity against the AML cell line MV4-11, with IC_50_ values ranging from 2.6 ± 0.4 to 52.2 ± 1.7 µM (Table 3, entries 1-7). Compound **44** was the most active compound but remained nearly 90-fold less active than the reference drug **SGI-1776** on this cell (Table 3, entry 11). Interestingly, compounds **44**, **45**, **16**, and **17**, exhibiting the best in vitro cytotoxic effects (IC_50_ values from 2.6 ± 0.4 to 14.3 ± 1.1 µM), also possessed the best enzymatic activity inhibition against HsPim1/2 (IC_50_ values from 0.06 to 0.28 µM on Pim-1 and IC_50_ values from 0.035 to 0.20 µM on Pim-2) with additional impact on CLK1 kinase (IC_50_ values from 0.026 to 0.45 µM). Notably, compound **46**, slightly less active on MV4-11 cells (Table 3, entry 7), and compounds **15** and **43**, with decreased cell growth inhibition on the same cell line (Table 3, entries 1 and 4), showed increased IC_50_ values against HsPim-1 (IC_50_ of 1.20 µM, for compound **46** and 0.82 µM, for compound **43**) or CLK1 (IC_50_ = 1.22 µM, for compound **15**) (Table 1, entries 15, 9, and 3). Moreover, the inactive compound **47** against the MV4-11 cell lines displayed a higher IC_50_ value of 2.35 µM against Pim-1 and was also inactive towards CLK1 kinase. It must be noticed that the majority of dihydroxydibenzofuran derivatives designed in the frame of that project showed better activity than cercosporamide (IC_50_ = 31.5 ± 4.7 µM) against MV4-11 cells.

Regarding the antileukemia effect of the compounds, it is noteworthy that disappointing pharmacological results against CML cell lines, namely KU812 and K562, suggested a selective effect on AML cell growth (Table 3). Considering the other types of tumors, all the tested compounds remained totally inactive against the MCF-7 cell line, except **44** (IC_50_ = 52.5 ± 1.2 µM) with moderate potency. In the contrary, HeLa cells proved to be very sensitive to part of the synthesized compounds, with IC_50_ values ranging from 9.5 ± 4.1 to 24.7 ± 6.7 µM, for the most active compounds and at the level of cercosporamide (Table 3, entry 9) for analogues **44** and **17** (Table 3, entries 5 and 3).

**Figure 4:**
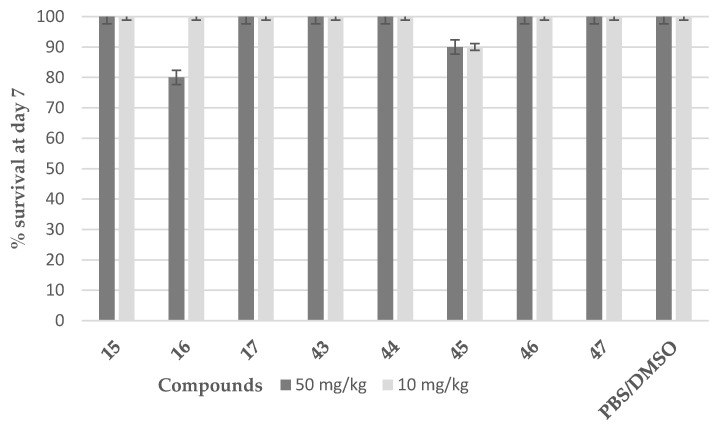
Evaluation of the in vivo cytotoxicity of dibenzofuran derivatives **15**–**17** and **43**–**47** on the *G. mellonella* model. Percentage of survival 7 days after injection of doses of 10 and 50 mg/kg for each compound. Mean of two independent experiments.

Against L929 normal cells, no cytotoxicity (Table 3, entries 2–4 and 6) or poor cytotoxicity (Table 3, entries 1 and 5) was observed, strengthening the interest in the series in terms of the selectivity index. Complementing this study, the determination of in vivo toxicity, using *Galleria mellonella* larvae [22], was performed for all the compounds involved in the antitumor assays. Indeed, *Galleria mellonella* has been demonstrated to be a more robust assessment of the likely toxicity of chemicals in mammals than cell lines [58]. Groups of 10 larvae were injected with 10 µL of compound at a dose varying from 10 to 50 mg/kg. The mortality was recorded daily for 7 days. Percentage survival was plotted for each compound using Graph Pad Prism. No reduction of viability and no sign of cuticular darkening were observed for any compounds, except for **16** and **45**, at a dose of 50 mg/kg after 7 days (Figure 4). Survival analysis was done using the log-rank test and the Kaplan–Meier survival curves for these two compounds. Concerning **16**, the 2 tests showed non-significant p-values (0.1464 and 0.1468, respectively) and allowed the conclusion that the survival curve was not significantly different to the other compounds. Concerning **45**, the same conclusions were obtained with p-values equal to 0.3173 for the two tests. These encouraging results prompted us to conclude to a general nontoxic effect in vivo in this model and at the considered dose.

## 3. Materials and Methods

### 3.1. Chemistry

#### 3.1.1. General Experimental Procedures

All commercial reagents were used without further purification. All solvents were reagent or HPLC grade. Analytical TLC was performed on silica gel 60 F254 plates. Open column chromatography was performed on silica gel 60 (70–230 mesh ASTM, Macherey-Nagel GmbH & Co. KG, Düren, Germany). The flash column chromatography was performed using a Reveleris^®^ X2 Buchi system, with a Buchi cartridge. Yields refer to chromatographically and spectroscopically pure compounds. Melting points were determined on an electrothermal melting point (Thermo Fisher Scientific, Illkirch, France) apparatus. ^1^H NMR and ^13^C NMR spectra were recorded in CDCl_3_ or in DMSO-*d*_6_ on a 400 MHz spectrometer. Chemical shifts are reported as δ values in parts per million (ppm) relative to tetramethylsilane as the internal standard and coupling constants (J) are given in hertz. Multiplicities are reported as follows: s = singlet, d = doublet, dd = doublet of doublets, t = triplet, mt = multiplet, bs = broad singlet, t_app_ = apparent triplet. All the spectra are provided in the “Supplementary Materials”. Low-resolution mass spectra were recorded using an electrospray ionization (ESI) method with a Waters ZQ 2000 spectrometer (Waters, Saint Quentin en Yvelines, France). The UPLC column used was an Acquity UPLC^®^ BEH Phenyl (2.1 mm i.d., 50 mm length, 1.7 µm particle size, Waters, Saint Quentin en Yvelines, France) from Waters. A linear mobile phase gradient was used with mobile phase A as 100% of acetonitrile in water (at 2%) and mobile phase B as 100% acetonitrile. The gradient table was: 0–0.5 min, 0% B; 0.5–4.0 min 0→100% B; 4.0–5.5% 100% B; 5.5–5.7 min 100→0% B; 5.7–7.5 min 0% B at a flow rate of 0.5 mL·min^−1^ and column temperature of 35 °C. Formic acid (0.1%) was added in diluent to improve ionization. For compounds **14**–**19** and **38**–**47**, high-resolution mass spectrometry (HRMS) was recorded on a Waters Vion IMS QTof instrument (SAA055K, Waters, Manchester, UK) coupled with an Acquity H-Class UPLC in ESI+ mode. IR absorption spectra were recorded on an ATR-FTIR equipment, MIRacle Shimadzu spectrometer (Shimadzu Corporation, Kyoto, Japan). Only the most significant bands were reported. Microwave reactions were carried out in a CEM Discover microwave reactor (CEM SAS, Saclay, France) in sealed vessels (monowave, maximum power 300 W, temperature control via IR-sensor, fixed temperature).

Compounds **1**, **3**, and cercosporamide were synthesized or obtained following procedures previously described [16].

#### 3.1.2. Access to Dibenzofuran Derivatives **14**–**19** by Intramolecular C-C Bond Formation from Diaryl Ethers

##### 2-Hydroxy-4,6-dimethoxybenzamide (**2**)

To a stirred suspension of 3,5-dimethoxyphenol **4** (1.00 g, 6.5 mmol) in acetonitrile (20 mL) at 0 °C under argon, chlorosulfonyl isocyanate (1.26 mL, 13 mmol) was added. The mixture was maintained at this temperature for 10 min and then the reaction mixture was quenched with HCl 5 M (20 mL). After 10 h at room temperature, the reaction was diluted with water (100 mL) and extracted with dichloromethane (3 × 50 mL). The combined organic extracts were dried over sodium sulfate and filtered. The solvent was evaporated in vacuo and the residue was purified by silica gel column chromatography using cyclohexane/ethyl acetate (8/2) as eluent to give compound **2** (576 mg, 45% yield) as a white powder. *R*_f_ 0.46 (cyHex/AcOEt 6:4); Mp: 154–155 °C; ^1^H NMR (400 MHz, DMSO-*d*_6_) δ 14.69 (s, 1H, OH), 8.04–7.86 (m, 2H, CONH_2_), 6.08 (d, *J* = 2.5 Hz, 1H, H_5_), 6.05 (d, *J* = 2.4 Hz, 1H, H_3_), 3.87 (s, 3H, CH_3_), 3.77 (s, 3H, CH_3_); ^13^C NMR (100 MHz, DMSO-*d*_6_) δ 171.7 (C=O), 165.9 (C_2_), 163.7 (C_6_), 160.2 (C_4_), 96.6 (C_1_), 94.1 (C_3_), 90.1 (C_5_), 56.0 (CH_3_), 55.3 (CH_3_); IR (cm^−1^): 3429, 3209, 1620, 1587, 1492, 1415, 1355, 1315, 1294, 1217, 1199, 1159, 1109, 1041; MS (ESI) *m*/*z* (%): 198.1 (100) [M + H]^+^; UPLC purity = 97%, *t*_R_ 1.93 min.

##### 2,4-Dimethoxy-6-(2′-nitrophenoxy)benzamide (**6**)

A mixture of 2-hydroxy-4,6-dimethoxybenzamide **2** (0.50 g, 2.5 mmol) and potassium hydroxide (0.13 g, 2.5 mmol) was heated to 120 °C and stirred for 30 min, and then 1-iodo-2-nitrobenzene (0.64 g, 2.5 mmol) and copper powder (0.20 g, 3.13 mmol) were added, respectively. The resulting mixture was stirred at 170 °C for 24 h and then cooled down to room temperature. After the addition of water, the aqueous layer was extracted with ethyl acetate (100 mL) and washed with water (2 × 100 mL). The organic layers were washed with brine, dried over sodium sulfate, filtered, and evaporated to dryness. The resulting oil was purified by silica gel column chromatography using cyclohexane/ethyl acetate (8/2) as an eluent to give compound **6** (125 mg, 15% yield) as a brown powder. *R*_f_ 0.45 (cyHex/AcOEt 8:2); Mp: 209–210 °C; ^1^H NMR (400 MHz, DMSO-*d*_6_) δ 13.51 (s, 1H, NH), 11.76 (s, 1H, NH), 8.60 (dd, *J* = 8.4, 1.5 Hz, 1H, H_3′_), 8.24 (dd, *J* = 8.4, 1.5 Hz, 1H, H_6′_), 7.85 (t, *J* = 7.9 Hz, 1H, H_4′_), 7.46 (t, *J* = 7.9 Hz, 1H, H_5′_), 6.31 (d, *J* = 2.3 Hz, 1H, H_5_), 6.24 (d, *J* = 2.3 Hz, 1H, H_3_), 4.10 (s, 3H, CH_3_), 3.89 (s, 3H, CH_3_); ^13^C NMR (100 MHz, DMSO-*d*_6_) δ 168.5 (C=O), 165.7 (C_4_), 164.6 (C_2_), 159.9 (C_6_), 139.6 (C_1′_), 134.8 (C_4′_), 132.0 (C_2′_), 125.4 (C_5′_), 124.8 (C_3′_), 124.8 (C_6′_), 97.2 (C_1_), 94.7 (C_5_), 91.0 (C_3_), 56.5 (CH_3_), 55.6 (CH_3_); IR (cm^−1^): 3284, 1639, 1575, 1502, 1390, 1346, 1276, 1249, 1157, 1047; MS (ESI) *m*/*z* (%): 319.1 (100) [M + H]^+^; UPLC purity = 97%, *t*_R_ 3.21 min.

##### 1,3-Dibenzyloxy-5-(2′-nitrophenoxy)benzene (**7**)

A mixture of 3,5-dibenzyloxyphenol **3** (1.00 g, 3.26 mmol) and potassium hydroxide (0.18 g, 3.26 mmol) was heated to 120 °C and stirred for 30 min, and then 1-iodo-2-nitrobenzene (0.82 g, 3.26 mmol) and copper powder (0.40 g, 6.25 mmol) were added, respectively. The resulting mixture was stirred at 170 °C for 2 h and then cooled down to room temperature. After the addition of water, the aqueous layer was extracted with ethyl acetate (100 mL) and washed with water (2 × 100 mL). The organic layers were washed with brine, dried over sodium sulfate, filtered, and evaporated to dryness. The resulting oil was purified by silica gel column chromatography using cyclohexane/ethyl acetate (8/2) as the eluent to give compound **7** (1.02 g, 73% yield) as a yellow powder. *R*_f_ 0.76 (cyHex/AcOEt 6:4); Mp: 148–149 °C; ^1^H NMR (400 MHz, CDCl_3_) δ 7.80 (dd, *J* = 8.1, 1.7 Hz, 1H, H_3′_), 7.35 (ddd, *J* = 8.5, 7.4, 1.7 Hz, 1H, H_4′_), 7.34–7.15 (m, 10H, H_Ar_), 7.12 (ddd, *J* = 8.5, 7.4, 1.7 Hz, 1H, H_5′_), 6.93 (dd, *J* = 8.4, 1.3 Hz, 1H, H_6′_), 6.33 (t, *J* = 2.2 Hz, 1H, H_2_), 6.17 (s, 1H, H_4_), 6.17 (s, 1H, H_6_), 4.86 (s, 4H, 2CH_2_); ^13^C NMR (100 MHz, CDCl_3_) δ 160.9 (2C_1,3_), 157.7 (C_5_), 150.0 (C_1′_), 141.5 (C_2′_), 136.4 (2C), 134.2 (C_4′_), 128.6 (2C), 128.6 (2C), 128.1 (1C), 128.1 (1C), 127.6 (2C), 127.6 (2C), 125.7 (C_4′_), 123.5 (C_3′_), 121.3 (C_6′_), 98.7 (2C_4,6_), 98.2 (C_2_), 70.3 (2CH_2_); IR (cm^−1^): 1589, 1529, 1450, 1355, 1236, 1157, 1128; MS (ESI) *m*/*z* (%): 428.2 (100) [M + H]^+^; UPLC purity = 100%, *t*_R_ 3.09 min.

##### 1,3-Dimethoxy-5-(2′-nitrophenoxy)benzene (**8**)

A mixture of 3,5-dimethoxyphenol **4** (1.00 g, 6.5 mmol) and potassium hydroxide (0.18 g, 6.5 mmol) was heated to 120 °C and stirred for 30 min, and then 1-iodo-2-nitrobenzene (0.82 g, 6.5 mmol) and copper powder (1.00 g, 15.6 mmol) were added, respectively. The resulting mixture was stirred at 170 °C for 2 h and then cooled down to room temperature. After the addition of water, the aqueous layer was extracted with ethyl acetate (100 mL). The organic layers were washed with brine, dried over sodium sulfate, filtered, and evaporated to dryness. The resulting oil was purified by silica gel column chromatography using cyclohexane/ethyl acetate (8/2) as eluent to give compound **8** (1.4 g 78% yield) as a yellow oil. *R*_f_ 0.42 (cyHex/AcOEt 8:2); ^1^H NMR (400 MHz, DMSO-*d*_6_) δ 8.07 (dd, *J* = 8.2 Hz, 1.6 Hz, 1H, H_3′_), 7.77–7.63 (m, 1H, H_5′_), 7.38 (t, *J* = 7.8 Hz, 1H, H_4′_), 7.21 (d, *J* = 8.4 Hz, 1H, H_6′_), 6.38 (t, *J* = 2.3 Hz, 1H, H_2_), 6.24 (s, 2H, H_4,6_), 3.74 (s, 6H, 2CH_3_); ^13^C NMR (100 MHz, DMSO-*d*_6_) δ 161.4 (2C_1,3_), 157.2 (C_5_), 148.8 (C_1′_), 141.0 (C_2′_), 134.9 (C_4′_), 125.5 (C_5′_), 124.2 (C_3′_), 120.9 (C_6′_), 97.1 (2C_4,6_), 96.4 (C_2_), 55.4 (2CH_3_); IR (cm^−1^): 1585, 1523, 1471, 1346, 1234, 1203, 1149, 1126, 1049; MS (ESI) *m*/*z* (%): 276.1 (100) [M + H]^+^; UPLC purity = 97%, *t*_R_ 3.02 min.

##### 2-(3′,5′-Dibenzyloxyphenoxy)aniline (**9**)

To a solution of 1,3-dibenzyloxy-5-(2′-nitrophenoxy)benzene **7** (100 mg, 0.23 mmol) in methanol (10 mL) at room temperature, saturated aqueous ammonium chloride (5 mL) and zinc dust (75 mg, 1.15 mmol) were added sequentially. After stirring for 30 min at room temperature, additional zinc was added (75 mg, 1.15 mmol) and the reaction mixture was refluxed for 2 h. After the addition of water, the aqueous layer was extracted with dichloromethane (3 × 50 mL). The combined organic extracts were dried over sodium sulfate and filtered. The solvent was evaporated in vacuo and the residue was purified by silica gel column chromatography using cyclohexane/ethyl acetate (8/2) as the eluent to give compound **9** (59 mg, 65% yield) as a brown powder. *R*_f_ 0.77 (cyHex/AcOEt 6:4); Mp: 217–218 °C; ^1^H NMR (400 MHz, CDCl_3_) δ 7.37–7.12 (m, 10H, H_Ar_), 6.89 (td, *J* = 7.6, 1.5 Hz, 1H, H_5_), 6.80 (dd, *J* = 8.0, 1.5 Hz, 1H, H_6_), 6.68 (dt, *J* = 8.6, 2.6 Hz, 1H, H_3_), 6.65 (td, *J* = 7.6, 1.5 Hz, 1H, H_4_), 6.24 (s, 1H, H_4′_), 6.14 (s, 2H, H_2′,6′_), 4.87 (s, 4H, 2CH_2_); ^13^C NMR (100 MHz, CDCl_3_) δ 160.8 (2C_3′,5′_), 159.5 (C_1′_), 142.4 (C_2_), 138.9 (C_1_), 136.6 (2C), 128.6 (4C), 128.0 (2C), 127.6 (4C), 125.3 (C_5_), 120.9 (C_4_), 118.8 (C_3_), 116.6 (C_6_), 96.7 (2C_2′,6′_), 96.4 (C_4′_), 70.1 (2CH_2_); IR (cm^−1^): 2819, 1597, 1492, 1450, 1379, 1253, 1157, 1126, 1060; MS (ESI) *m*/*z* (%): 398.1 (100) [M + H]^+^; UPLC purity = 100%, *t*_R_ 3.12 min.

##### 2-(3′,5′-Dimethoxyphenoxy)aniline (**10**)

To a solution of 1,3-dimethoxy-5-(2′-nitrophenoxy)benzene **8** (250 mg, 0.91 mmol) in methanol (10 mL) at room temperature, saturated aqueous ammonium chloride (5 mL) and zinc dust (295 mg, 4.54 mmol) were added sequentially. After stirring for 30 min at room temperature, additional zinc was added (295 mg, 4.54 mmol) and the reaction mixture was refluxed for 2 h. After the addition of water, the aqueous layer was extracted with ethyl acetate (100 mL) and washed with water (2 × 50 mL). The combined organic extracts were dried over sodium sulfate and filtered. The solvent was evaporated *in vacuo* and the residue was purified by silica gel column chromatography using cyclohexane/ethyl acetate (6/4) as the eluent to give compound **7** (205 mg, 92% yield) as a brown oil. *R*_f_ 0.80 (cyHex/AcOEt 6:4); ^1^H NMR (400 MHz, CDCl_3_) δ 6.89 (td, *J* = 7.6, 1.5 Hz, 1H, H_4_), 6.82 (dd, *J* = 8.0, 1.4 Hz, 1H, H_3_), 6.71 (dd, *J* = 7.9, 1.6 Hz, 1H, H_6_), 6.62 (td, *J* = 7.6, 1.6 Hz, 1H, H_5_), 6.09 (t, *J* = 2.2 Hz, 1H, H_4′_), 6.06 (d, *J* = 2.3 Hz, 1H, 2H_2′_), 6.05 (d, *J* = 2.3 Hz, 2H, 2H_6′_), 3.64 (s, 3H, CH_3_), 3.60 (s, 3H, CH_3_); ^13^C NMR (100 MHz, DMSO-*d*_6_) δ 161.2 (2C_3′_,_5′_), 159.4 (C_1′_), 141.0 (C_2_), 140.4 (C_1_), 125.1 (C_4_), 120.7 (C_5_), 116.4 (C_3_), 115.8 (C_6_), 95.1 (2C_2′_,_6′_), 94.0 (C_4′_), 55.0 (2CH_3_); IR (cm^−1^): 3373, 2964, 1697, 1595, 1500, 1473, 1458, 1427, 1301, 1269, 1197, 1149, 1130, 1051; MS (ESI) *m*/*z* (%): 246.1 (100) [M + H]^+^; UPLC purity = 100%, *t*_R_ 2.51 min.

##### 1,3-Dimethoxy-6-nitrodibenzo[*b*,*d*]furan (**13**)

1,3-Dimethoxy-5-(2′-nitrophenoxy)benzene **8** (1.00 g, 3.6 mmol) was dissolved in warm pivalic acid (50 °C, 60 g). Silver acetate (1.20 g, 7.2 mmol) and palladium (II) acetate (40 mg, 0.18 mmol) were added, and the reaction mixture was properly stirred at 130 °C for 4 h. After cooling to room temperature and the addition of water (100 mL), the aqueous layer was extracted with ethyl acetate (100 mL). The organic layers were washed with brine, dried over sodium sulfate, filtered, and evaporated to dryness. The resulting oil was purified by silica gel column chromatography using cyclohexane/ethyl acetate (8/2) as the eluent to give compound **13** (0.71 g, 72% yield) as a yellow powder. *R*_f_ 0.64 (cyHex/AcOEt 6:4); Mp: 212–213 °C; ^1^H NMR (400 MHz, DMSO-*d*_6_) δ 8.27 (dd, *J* = 7.6 Hz, 1.1 Hz, 1H, H_9_), 8.18 (dd, *J* = 8.3, 1.1 Hz, 1H, H_7_), 7.55 (t, *J* = 8.0 Hz, 1H, H_8_), 7.13 (d, *J* = 1.8 Hz, 1H, H_4_), 6.66 (d, *J* = 1.8 Hz, 1H, H_2_), 4.03 (s, 3H, CH_3_), 3.90 (s, 3H, CH_3_); ^13^C NMR (100 MHz, DMSO-*d*_6_) δ 162.2 (C_3_), 158.2 (C_1_), 155.5 (C), 147.1 (C), 132.9 (C_6_), 127.6 (C_8_), 126.9 (C), 123.7 (C_9_), 120.5 (C_7_), 104.4 (C), 95.3 (C_2_), 89.3 (C_4_), 56.1 (CH_3_), 56.1 (CH_3_); IR (cm^−1^): 3088, 2933, 1637, 1602, 1531, 1502, 1450, 1417, 1344, 1219, 1145, 1095; MS (ESI) *m*/*z* (%): 274.1 (100) [M + H]^+^; UPLC purity = 99%, *t*_R_ 3.16 min.

##### 1,3-Dimethoxy-6-nitrodibenzo[*b*,*d*]furan-4-carboxamide (**14**)

To a stirred suspension of 1,3-dimethoxy-6-nitrodibenzo[*b*,*d*]furan **13** (1.00 g, 3.7 mmol) in acetonitrile (20 mL) at 0 °C under argon, chlorosulfonyl isocyanate (0.64 mL, 7.4 mmol) was added. The mixture was maintained at room temperature for 12 h and then the reaction mixture was quenched with HCl 5 M (20 mL). After 6 h at room temperature, the reaction was diluted with water (100 mL) and extracted with ethyl acetate (100 mL). The combined organic extracts were dried over sodium sulfate and filtered. The solvent was evaporated *in vacuo* and the residue was purified by silica gel column chromatography using cyclohexane/ethyl acetate (6/4) as the eluent to give compound **14** (0.61 g, 53% yield) as a yellow powder. *R*_f_ 0.63 (CH_2_Cl_2_/MeOH 9:1); Mp: 298–299 °C; ^1^H NMR (400 MHz, DMSO-*d*_6_) δ 8.32 (d, *J* = 7.6 Hz, 1H, H_9_), 8.20 (d, *J* = 8.3 Hz, 1H, H_7_), 7.77 (s, 1H, NH), 7.67–7.54 (m, 2H, NH, H_8_), 6.82 (s, 1H, H_2_), 4.13 (s, 3H, CH_3_), 3.98 (s, 3H, CH_3_); ^13^C NMR (100 MHz, DMSO-*d*_6_) δ 163.7 (C=O), 158.7 (C_1_), 155.8 (C_3_), 154.8 (C), 147.1 (C), 133.0 (C_6_), 127.7 (C_8_), 126.6 (C), 123.8 (C_9_), 120.9 (C_7_), 105.1 (C), 104.3 (C_4_), 92.2 (C_2_), 56.8 (CH_3_), 56.4 (CH_3_); IR (cm^−1^): 3136, 3091, 2846, 2140, 1977, 1643, 1606, 1521, 1427, 1355, 1323, 1217, 108; MS (ESI) *m*/*z* (%): 317.2 (100) [M + H]^+^; UPLC purity = 99%, *t*_R_ 2.24 min; HRMS (TOF MS ES+): calcd. for C_15_H_13_N_2_O_6_ [M + H]^+^ 317.076813, found: 317.07664.

##### 1,3-Dihydroxy-6-nitrodibenzo[*b*,*d*]furan-4-carboxamide (**15**)

A mixture of 1,3-dimethoxy-6-nitrodibenzo[*b*,*d*]furan-4-carboxamide **14** (50 mg, 0.16 mmol) in pyridine hydrochloride (3g) was irradiated using microwave heating at 200 °C for 15 min. After the addition of water, the aqueous layer was extracted with ethyl acetate (50 mL) and the resulting organic layer was washed with water (2 × 50 mL). The combined organic extracts were dried over sodium sulfate and filtered. The solvent was evaporated *in vacuo* and the residue was purified by silica gel column chromatography using cyclohexane/ethyl acetate (6/4) as the eluent to give compound **15** (23 mg, 51% yield) as a yellow powder. *R*_f_ 0.67 (CH_2_Cl_2_/MeOH 9:1); Mp > 300 °C; ^1^H NMR (400 MHz, DMSO-*d*_6_) δ 13.80 (s, 1H, OH), 11.77 (s, 1H, OH), 8.54 (s, 1H, NH), 8.42–8.07 (m, 2H, NH, H_9_), 7.75–7.36 (m, 2H, H_8_,_7_), 6.39 (s, 1H, H_2_); ^13^C NMR (100 MHz, DMSO-*d*_6_) δ 169.6 (C=O), 165.1 (C_1_), 157.9 (C_3_), 155.7 (C), 146.3 (C), 133.0 (C_6_), 127.7 (C_8_), 126.5 (C), 124.5 (C_9_), 120.3 (C_7_), 103.1 (C_4_), 98.9 (C_2_), 92.7 (C); IR (cm^−1^): 3458, 3196, 1654, 1629, 1608, 1517, 1477, 1440, 1404, 1359, 1323, 1251, 1220, 1195, 1120, 1082; MS (ESI) *m*/*z* (%): 289.1 (100) [M + H]^+^; UPLC purity = 99%, *t*_R_ 2.37 min; HRMS (TOF MS ES+): calcd. for C_13_H_9_N_2_O_6_ [M + H]^+^ 289.045512, found: 289.04558.

##### 6-Amino-1,3-dihydroxydibenzo[*b*,*d*]furan-4-carboxamide (**16**)

To a solution of 1,3-dihydroxy-6-nitrodibenzo[*b*,*d*]furan-4-carboxamide **15** (50 mg, 0.17 mmol) in methanol (10 mL) at room temperature, saturated aqueous ammonium chloride (5 mL) and zinc dust (55 mg, 0.85 mmol) were added sequentially. After stirring for 30 min at room temperature, additional zinc was added (55 mg, 0.85 mmol) and the reaction mixture was refluxed for 2 h. After the addition of water, the aqueous layer was extracted with ethyl acetate (100 mL) and the resulting organic layer was washed with water (2 × 50 mL). The combined organic extracts were dried over sodium sulfate and filtered. The solvent was evaporated *in vacuo* and the residue was purified by silica gel column chromatography using cyclohexane/ethyl acetate (6/4) as the eluent to give compound **16** (28 mg, 62% yield) as a brown powder. *R*_f_ 0.34 (CH_2_Cl_2_/MeOH 9:1); Mp: 292-293 °C; ^1^H NMR (400 MHz, DMSO-*d*_6_) δ 14.19 (s, 1H, OH), 11.12 (s, 1H, OH), 8.19 (s, 1H, CONH_2_), 8.11 (s, 1H, CONH_2_), 7.12 (d, *J* = 7.5 Hz, 1H, H_9_), 7.03 (t, *J* = 7.6 Hz, 1H, H_8_), 6.63 (d, *J* = 7.8 Hz, 1H, H_7_), 6.25 (s, 1H, H_2_); ^13^C NMR (100 MHz, DMSO) δ 170.2 (C=O), 164.2 (C_1_), 157.7 (C_3_), 154.8 (C), 142.2 (C), 133.3 (C_6_), 124.4 (C_8_), 123.0 (C), 110.2 (C_9_), 108.1 (C_7_), 105.3 (C_4_), 97.8 (C_2_), 92.5 (C); IR (cm^−1^): 3294, 3192, 1492, 1427, 1261, 1190, 1085, 1053; MS (ESI) *m*/*z* (%): 259.2 (100) [M + H]^+^; UPLC purity = 99%, *t*_R_ 1.67 min; HRMS (TOF MS ES+): calcd. for C_13_H_11_N_2_O_4_ [M + H]^+^ 259.071333, found: 259.07147.

##### 1,3-Dihydroxydibenzo[*b*,*d*]furan-4-carboxamide (**17**)

Method A: To a solution of 6-amino-1,3-dihydroxydibenzo[*b*,*d*]furan-4-carboxamide **16** (100 mg, 0.38 mmol) in ethanol (10 mL) at room temperature, sulfuric acid (25%, 40 mL) was added under warming. The mixture was cooled to 0 °C, and a solution of sodium nitrite (54 mg, 0.76 mmol) in 5 mL of water was added dropwise with stirring for 30 min. Afterwards, the reaction mixture was stirred for 45 min at 80 °C and then the reaction was quenched with water. The aqueous layer was extracted with ethyl acetate (100 mL) and the organic layer was washed with brine, dried over sodium sulfate, and filtered. The solvent was evaporated in vacuo and the residue was purified by silica gel column chromatography using cyclohexane/ethyl acetate (6/4) as the eluent to give compound **17** (49 mg, 52% yield) as a brown powder.

Method B: A mixture of 1,3-dimethoxydibenzo[*b*,*d*]furan-4-carboxamide **19** (see below) (100 mg, 0.37 mmol) in pyridine hydrochloride (3 g) was irradiated using microwave heating at 200 °C for 15 min. After the addition of water, the aqueous layer was extracted with ethyl acetate (50 mL) and the resulting organic layer was washed with water (2 × 50 mL). The combined organic extracts were dried over sodium sulfate and filtered. The solvent was evaporated in vacuo and the residue was purified by silica gel column chromatography using cyclohexane/ethyl acetate (6/4) as the eluent to give compound **17** (43 mg, 48% yield) as a brown powder.

*R*_f_ 0.20 (CH_2_Cl_2_); Mp: 219–220 °C; ^1^H NMR (400 MHz, DMSO-*d*_6_) δ 13.93 (s, 1H, OH), 11.34 (s, 1H, OH), 8.28 (s, 1H, CONH_2_), 8.03–7.88 (m, 1H, H_9_), 7.85 (s, 1H, CONH_2_), 7.77–7.65 (m, 1H, H_6_), 7.57–7.25 (m, 2H, H_7,8_), 6.31 (s, 1H, H_2_); ^13^C NMR (100 MHz, DMSO-*d*_6_) δ 170.0 (C=O), 164.3 (C_1_), 157.7 (C_3_), 155.2 (C), 154.2 (C), 124.8 (C_7_), 123.7 (C_8_), 122.7 (C), 121.0 (C_9_), 111.3 (C_6_), 104.4 (C_4_), 98.0 (C_2_), 92.6 (C); IR (cm^−1^): 3456, 3132, 1604, 1446, 1409, 1348, 1315, 1286, 1230, 1195, 1103, 1053; MS (ESI) *m*/*z* (%): 244.1 (100) [M + H]^+^; UPLC purity = 98%, *t*_R_ 2.14 min; HRMS (TOF MS ES+): calcd. for C_13_H_10_NO_4_ [M + H]^+^ 244.060434, found: 244.06010.

##### 6-Amino-1,3-dimethoxydibenzo[*b*,*d*]furan-4-carboxamide (**18**)

To a solution of 1,3-dimethoxy-6-nitrodibenzo[*b*,*d*]furan-4-carboxamide **14** (50 mg, 0.16 mmol) in methanol (10 mL) at room temperature, saturated aqueous ammonium chloride (5 mL) and zinc dust (55 mg, 0.85 mmol) were added sequentially. After stirring for 30 min at room temperature, additional zinc was added (55 mg, 0.85 mmol) and the reaction mixture was refluxed for 2 h. After the addition of water, the aqueous layer was extracted with ethyl acetate (100 mL) and the resulting organic layer was washed with water (2 × 50 mL). The combined organic extracts were dried over sodium sulfate and filtered. The solvent was evaporated *in vacuo* and the residue was purified by silica gel column chromatography using cyclohexane/ethyl acetate (6/4) as eluent to give compound **18** (22 mg, 49% yield) as a brown powder. *R*_f_ 0.52 (CH_2_Cl_2_/MeOH 9:1); Mp: 293–294 °C; ^1^H NMR (400 MHz, DMSO-*d*_6_) δ 7.67 (s, 1H, NH), 7.44 (s, 1H, NH), 7.17 (d, *J* = 7.5 Hz, 1H, H_9_), 7.03 (t, *J* = 7.7 Hz, 1H, H_8_), 6.70 (d, *J* = 5.9 Hz, 1H, H_7_), 6.68 (s, 1H, H_2_), 5.25 (s, 2H, NH_2_), 4.05 (s, 3H, CH_3_), 3.93 (s, 3H, CH_3_); ^13^C NMR (100 MHz, DMSO-*d*_6_) δ 164.6 (C=O), 157.2 (C_1_), 155.5 (C_3_), 153.9 (C), 142.9 (C), 133.1 (C_6_), 123.7 (C_8_), 122.7 (C), 111.2 (C_9_), 109.1 (C_7_), 107.0 (C_4_), 104.8 (C), 90.9 (C_2_), 56.6 (CH_3_), 56.0 (CH_3_); IR (cm^−1^): 3180, 1643, 1604, 1492, 1413, 1319, 1211, 1101; MS (ESI) *m*/*z* (%): 287.2 (100) [M + H]^+^; UPLC purity = 99%, *t*_R_ 1.83 min; HRMS (TOF MS ES+): calcd. for C_15_H_15_N_2_O_4_ [M + H]^+^ 287.102633, found: 287.10220.

##### 1,3-Dimethoxydibenzo[*b*,*d*]furan-4-carboxamide (**19**)

Method A: To a solution of 6-amino-1,3-dimethoxydibenzo[*b*,*d*]furan-4-carboxamide **18** (50 mg, 0.17 mmol) in ethanol (10 mL) at room temperature, sulfuric acid (25%, 20 mL) was added under warming. The mixture was cooled to 0 °C, and a solution of sodium nitrite (24 mg, 0.34 mmol) in 5 mL of water was added dropwise with stirring for 30 min. The reaction mixture was stirred for 45 min at 80 °C and then the reaction was quenched with water. The aqueous layer was extracted with ethyl acetate (100 mL) and the organic layer was washed with brine, dried over sodium sulfate, and filtered. The solvent was evaporated *in vacuo* and the residue was purified by silica gel column chromatography using cyclohexane/ethyl acetate (6/4) as the eluent to give compound **19** (22 mg, 46% yield) as a pale brown powder.

Method B: A mixture of 2’-fluoro-2-hydroxy-4,6-dimethoxybiphenyl-3-carboxamide **49** (see below) (50 mg, 0.17 mmol) and cesium carbonate (166 mg, 0.51 mmol) in acetonitrile was irradiated using microwave (80 W) heating at 150 °C for 1 h. Ethyl acetate (50 mL) was added to the mixture and the organic layer was washed with water (2 × 50 mL), dried over sodium sulfate, and filtered. The solvent was evaporated in vacuo and the residue was crystallized from diisopropyl ether to furnish the title compound **19** as a pale brown solid (28 mg, 61%)

*R*_f_ 0.41 (CH_2_Cl_2_/MeOH 9:1); Mp: 296–297 °C; ^1^H NMR (400 MHz, DMSO-*d*_6_) δ 7.95 (dd, *J* = 7.5, 1.5 Hz, 1H, H_9_), 7.69 (s, 1H, NH), 7.65 (d, *J* = 8.0 Hz, 1H, H_6_), 7.51 (s, 1H, NH), 7.38 (m, 2H, H_7,8_), 6.72 (s, 1H, H_2_), 4.09 (s, 3H, CH_3_), 3.95 (s, 3H, CH_3_); ^13^C NMR (100 MHz, DMSO-*d*_6_) δ 164.5 (C=O), 157.6 (C_1_), 155.7 (C_3_), 154.8 (C), 154.4 (C), 125.5 (C_7_), 123.2 (C_8_), 122.5 (C), 121.3 (C_9_), 110.9 (C_6_), 105.9 (C_4_), 104.7 (C), 91.1 (C_2_), 56.7 (CH_3_), 56.1 (CH_3_); IR (cm^−1^): 3381, 3201, 2968, 1641, 1602, 1452, 1421, 1321, 1213; MS (ESI) *m*/*z* (%): 272.2 (100) [M + H]^+^; UPLC purity = 99%, *t*_R_ 2.21 min; HRMS (TOF MS ES+): calcd. for C_15_H_14_NO_4_ [M + H]^+^ 272.091734, found: 272.09149.

Access to dibenzofuran derivatives **38**–**47** was achieved by intramolecular C-C bond formation from diaryl ethers.

#### 3.1.3. Preparation of Starting Iodo-Aryl Derivatives **21**–**24**

##### 3-Iodophenyl acetate (**21**)

Acetic anhydride (4.3 mL, 45.47 mmol) was added to a solution of 3-iodophenol **20** (1.00 g, 4.55 mmol) in pyridine (5 mL). After stirring at 130 °C for 2 h, the reaction was concentrated in vacuo and the residue was purified by silica gel column chromatography using cyclohexane/ethyl acetate (8/2) to give compound **21** (1.06 g, 90% yield) as a white solid. *R*_f_ 0.65 (cyHex/AcOEt 8:2); Mp: 33–34 °C (lit. 32.4–34.6 °C) [59]; ^1^H NMR (400 MHz, CDCl_3_) δ 7.49 (dt, *J* = 7.2, 1.7 Hz, 1H), 7.45–7.28 (m, 1H), 7.11–6.88 (m, 2H), 2.21 (s, 3H, CH_3_); ^13^C NMR (100 MHz, CDCl_3_) δ 168.9 (C=O), 150.9 (C_1_), 134.9 (C_4_), 130.8 (C_5_), 130.6 (C_2_), 121.1 (C_6_), 93.5 (C_3_), 21.0 (CH_3_); IR (cm^−1^): 1747, 1735, 1573, 1365, 1201, 1192, 1072, 1031, 1001, 981; MS (ESI) *m*/*z* (%): 263.1 (100) [M + H]^+^; UPLC purity = 100%, *t*_R_ 2.60 min. Literature spectroscopic data Reference [59].

##### 1-(2-Hydroxy-4-iodophenyl)ethanone (**22**)

3-Iodophenyl acetate **21** (250 mg, 0.95 mmol) was dissolved in boron trifluoride acetic acid complex (4 mL). The mixture was stirred at 180 °C for 12 h. The reaction medium was allowed to cool and then was diluted in dichloromethane (20 mL). Then, 5M Sodium hydroxide aqueous solution was added (20 mL). The aqueous layer was extracted with dichloromethane (100 mL) and washed with distilled water (100 mL). The aqueous layers were acidified with 5M hydrochloric acid solution (20 mL) and extracted with dichloromethane. The combined organic layers were dried over sodium sulfate and filtered. The solvent was evaporated *in vacuo* and the residue was purified by silica gel column chromatography using cyclohexane/ethyl acetate (8/2) as the eluent to give compound **22** (219 mg, 88% yield) as a beige powder. *R*_f_ 0.68 (cyHex/AcOEt 8:2); Mp: 52–53 °C (lit. 51.5–52 °C) [59]; ^1^H NMR (400 MHz, CDCl_3_) δ 12.18 (s, 1H, OH), 7.50–7.24 (m, 2H), 7.27–7.07 (m, 1H), 2.53 (s, 3H, CH_3_); ^13^C NMR (100 MHz, CDCl_3_) δ 204.1 (C=O), 162.2 (C_2_), 131.2 (C_5_), 128.3 (C_6_), 127.8 (C_3_), 119.0 (C_1_), 103.7 (C_4_), 26.5 (CH_3_); IR (cm^−1^): 1620, 1600, 1543, 1475, 1365, 1317, 1284, 1211; MS (ESI) *m*/*z* (%): 263.0 (100) [M + H]^+^; UPLC purity = 100%, *t*_R_ 2.68 min. Literature spectroscopic data [59].

##### 1-(4-Iodo-2-methoxyphenyl)ethanone (**23**)

1-(2-Hydroxy-4-iodophenyl)ethanone **22** (1.00 g, 3.8 mmol) and cesium carbonate (3.71 g, 11.4 mmol) were introduced in a round-bottom flask under argon. Dry acetonitrile (30 mL) was added, followed by iodomethane (490 µL, 7.6 mmol). The reaction mixture was stirred at 70 °C for 2 h and then the reaction was diluted with water (50 mL) and extracted with dichloromethane (2 × 50 mL). The combined organic extracts were dried over sodium sulfate and filtered. The solvent was evaporated *in vacuo* and the residue was purified by silica gel column chromatography using cyclohexane/ethyl acetate (8/2) to give compound **23** as a white powder (0.95 g, 91% yield). *R*_f_ 0.53 (cyHex/AcOEt 8:2); Mp: 70–71 °C (lit. 69–70 °C [60]); ^1^H NMR (400 MHz, DMSO-*d*_6_) δ 7.53 (d, *J* = 1.4 Hz, 1H, H_3_), 7.42 (dd, *J* = 8.1, 1.5 Hz, 1H, H_5_), 7.32 (d, *J* = 8.1 Hz, 1H, H_6_), 3.91 (s, 3H), 3.33 (s, 3H, CH_3_); ^13^C NMR (100 MHz, DMSO-*d*_6_) δ 198.1 (C=O), 158.5 (C_2_), 130.8 (C_5_), 129.4 (C_6_), 127.3 (C_1_), 121.4 (C_3_), 101.0 (C_4_), 56.2 (CH_3_), 31.4 (CH_3_); MS (ESI) *m*/*z* (%): 277.0 (100) [M + H]^+^; UPLC purity = 97%, *t*_R_ 2.62 min.

##### 1-Iodo-3-methoxybenzene (**24**)

3-Iodophenol **20** (1.00 g, 4.5 mmol) and cesium carbonate (2.40 g, 6.8 mmol) were introduced in a round-bottom flask under argon. Dry acetonitrile (30 mL) was added, followed by iodomethane (420 µL, 6.8 mmol). The reaction mixture was stirred at 70 °C for 4 h and then the reaction was diluted with water (50 mL) and extracted with dichloromethane (3 × 50 mL). The combined organic extracts were dried over sodium sulfate and filtered. The solvent was evaporated *in vacuo* and the residue was purified by silica gel column chromatography using cyclohexane/ethyl acetate (9/1) as the eluent to give compound **24** (940 mg, 89% yield) as a yellow oil. *R*_f_ 0.57 (cyHex/AcOEt 8:2); ^1^H NMR (400 MHz, CDCl_3_) δ 7.24–7.14 (m, 2H, H_2,6_), 6.91 (t, *J* = 8.0 Hz, 1H, H_5_), 6.78 (ddd, *J* = 8.4, 2.5, 0.9 Hz, 1H, H_4_), 3.69 (s, 3H, OCH_3_); ^13^C NMR (100 MHz, CDCl_3_) δ 160.1 (C_3_), 130.7 (C_6_), 129.8 (C_5_), 123.0 (C_2_), 113.7 (C_4_), 94.3 (C_1_), 55.3 (CH_3_); MS (ESI) *m*/*z* (%): 235.1 (100) [M + H]^+^; UPLC purity = 97%, *t*_R_ 2.87 min. Literature spectroscopic data [61].

#### 3.1.4. General Procedure for the Preparation of Diaryl Ethers **28**–**32**

To a solution of 3,5-dimethoxyphenol **4** (1 equiv.) in *N*,*N-*dimethylformamide, cesium carbonate (2 equiv.), copper(1) iodide (0.1 equiv.), and iodo-aryl derivatives **23**–**27** (1.5 equiv.) were added. The reaction mixture was irradiated using microwave heating at 110 °C for 1 h. After cooling to room temperature, the mixture was diluted with ethyl acetate (50 mL) and water (100 mL). The suspension was filtered through a pad of Celite^®^. The two layers were separated, and the organic layer was washed with water (3 × 50 mL), dried over sodium sulfate, filtered, and evaporated to dryness. The crude product was then purified by chromatography to provide **28**–**32**.

##### 1-[4-(3′,5′-Dimethoxyphenoxy)-2-methoxyphenyl]ethanone (**28**)

The reaction was carried out using 3,5-dimethoxyphenol **4** (400 mg, 2.6 mmol), Cs_2_CO_3_ (1.69 g, 5.2 mmol), CuI (50 mg, 0.26 mmol), and 1-(4-iodo-2-methoxyphenyl)ethanone **23** (1.08 g, 3.9 mmol) in DMF (3 mL). Purification by flash column chromatography using cyclohexane/ethyl acetate (8/2) as the eluent afforded compound **28** (266 mg, 34% yield) as a colorless oil. *R*_f_ 0.54 (cyHex/AcOEt 8:2); ^1^H NMR (400 MHz, CDCl_3_) δ 7.78 (d, *J* = 8.8 Hz, 1H, H_6_), 6.61 (d, *J* = 2.2 Hz, 1H, H_3_), 6.56 (dd, *J* = 8.7, 1.4 Hz, 1H, H_5_), 6.29 (t, *J* = 2.2 Hz, 1H, H_4′_), 6.22–6.21 (m, 2H, H_2′,6′_), 3.86 (s, 3H, CH_3_), 3.76 (s, 6H, 2CH_3_), 2.59 (s, 3H, COCH_3_); ^13^C NMR (100 MHz, CDCl_3_) δ 198.2 (C=O), 162.3 (C_2_), 161.9 (2C_3′,5′_), 161.0 (C_4_), 157.5 (C_1′_), 132.6 (C_6_), 123.0 (C_1_), 109.9 (C_5_), 101.9 (C_3_), 98.5 (2C_2′,6′_), 96.9 (C_4′_), 55.8 (OCH_3_), 55.6 (2OCH_3_), 31.9 (COCH_3_); IR (cm^−1^): 2839, 1644, 1579, 1462, 1419, 1357, 1257, 1195, 1149, 1128, 1051, 1028; MS (ESI) *m*/*z* (%): 303.2 (100) [M + H]^+^; UPLC purity = 98%, *t*_R_ 2.98 min.

##### 1,3-Dimethoxy-5-(3′-methoxyphenoxy)benzene (**29**)

The reaction was carried out using 3,5-dimethoxyphenol **4** (200 mg, 1.30 mmol), Cs_2_CO_3_ (847 mg, 2.60 mmol), CuI (25 mg, 0.13 mmol), and 1-iodo-3-methoxybenzene **24** (455 mg, 1.95 mmol) in DMF (3 mL). Purification by flash column chromatography using cyclohexane/ethyl acetate (9/1) as the eluent afforded compound **29** (103 mg, 30% yield) as a colorless oil. *R*_f_ 0.55 (cyHex/AcOEt 8:2); ^1^H NMR (400 MHz, CDCl_3_) δ 7.15 (t, *J* = 8.1 Hz, 1H, H_5′_), 6.64–6.49 (m, 3H, H_2′,4′,6′_), 6.15 (t, *J* = 2.2 Hz, 1H, H_2_), 6.11 (d, *J* = 2.4 Hz, 1H), 6.11 (d, *J* = 2.4 Hz, 1H), 3.71 (s, 3H, CH_3_), 3.67 (s, 6H, 2CH_3_).; ^13^C NMR (100 MHz, CDCl_3_) δ 161.5 (2C_1,3_), 160.9 (C_3′_), 158.9 (C_1′_), 157.9 (C_5_), 130.0 (C_5_), 111.3 (C_6′_), 109.1 (C_2′_), 105.1 (C_4′_), 97.4 (2C_4,6_), 95.6 (C_2_), 55.4 (2CH_3_), 55.3 (CH_3_); IR (cm^−1^): 2835, 1583, 1261, 1193, 1143, 1126, 1047; MS (ESI) *m*/*z* (%): 261.1 (100) [M + H]^+^; UPLC purity = 99%, *t*_R_ 3.20 min.

##### 1-[4-(3′,5′-Dimethoxyphenoxy)phenyl]ethanone (**30**)

The reaction was carried out using 3,5-dimethoxyphenol **4** (1.00 g, 6.5 mmol), Cs_2_CO_3_ (4.23g, 13 mmol), CuI (124 mg, 0.65 mmol), and 4-iodoacetophenone **25** (2.40 g, 9.7 mmol) in DMF (5 mL). Purification by flash column chromatography using cyclohexane/ethyl acetate (8/2) as the eluent afforded compound **30** (1.20 g, 71% yield) as a brown powder. *R*_f_ 0.60 (cyHex/AcOEt 8:2); Mp: 85–86 °C; ^1^H NMR (400 MHz, CDCl_3_) δ 7.96 (mt, 2H, H_2,6_), 7.05 (mt, 2H, H_3,5_), 6.32 (t, *J* = 2.2 Hz, 1H, H_4′_), 6.24 (d, *J* = 2.3 Hz, 2H, H_2′,6′_), 3.79 (s, 6H, 2CH_3_), 2.60 (s, 3H, COCH_3_); ^13^C NMR (100 MHz, CDCl_3_) δ 196.7 (C=O), 161.7 (2C_3′,5′_), 161.5 (C_4_), 157.3 (C_1′_), 132.1 (C_1_), 130.5 (2C_2,6_), 117.6 (2C_3,5_), 98.5 (2C_2′,6′_), 96.7 (C_4′_), 55.4 (2CH_3_), 26.4 (COCH_3_); IR (cm^−1^): 1674, 1585, 1425, 1361, 1267, 1230, 1136, 1053; MS (ESI) *m*/*z* (%): 273.2 (100) [M + H]^+^; UPLC purity = 99%, *t*_R_ 2.92 min.

##### 1-(4′-Fluorophenoxy)-3,5-dimethoxybenzene (**31**)

The reaction was carried out using 3,5-dimethoxyphenol **4** (1.00 g, 6.5 mmol), Cs_2_CO_3_ (4.23 g, 13 mmol), CuI (124 mg, 0.65 mmol), and 1-fluoro-4-iodobenzene **26** (1.1 mL, 9.75 mmol) in DMF (5 mL). Purification by flash column chromatography using cyclohexane/ethyl acetate (8/2) as the eluent afforded compound **31** (1.23 g, 76% yield) as a yellow oil. *R*_f_ 0.30 (cyHex/CH_2_Cl_2_ 8:2); ^1^H NMR (400 MHz, DMSO-*d*_6_) δ 7.22 (t, *J* = 8.4 Hz, 2H, H_3′,5′_), 7.08 (dd, *J* = 8.4, 4.6 Hz 2H, H_2′,6′_), 6.28 (t, *J* = 2.4Hz, 1H, H_4_), 6.11 (d, *J* = 2.4Hz, 2H, H_2,6_), 3.70 (s, 6H, 2CH_3_); ^13^C NMR (100 MHz, DMSO-*d*_6_) δ 161.3 (2C_3,5_), 158.9 (C_1_), 158.3 (d, *J_CF_* = 238 Hz, C_4′_), 152.1 (d, *J_CF_* = 2 Hz, C_1′_), 120.8 (d, *J_CF_* = 8 Hz, 2C_2′,6′_), 116.4 (d, *J_CF_* = 24 Hz, 2C_3′,5′_), 96.6 (2C_2,6_), 95.3 (C_4_), 55.2 (2CH_3_); IR (cm^−1^): 2943, 2839, 1591, 1499, 1462, 1238, 1150, 824; MS (ESI) *m*/*z* (%): 249.1 (100) [M + H]^+^; UPLC purity = 100%, *t*_R_ 3.55 min.

##### 1,3-Dimethoxy-5-[4′-(trifluoromethyl)phenoxy]benzene (**32**)

The reaction was carried out using 3,5-dimethoxyphenol **4** (500 mg, 3.25 mmol), Cs_2_CO_3_ (2.12 g, 6.5 mmol), CuI (62 mg, 325 µmol), and 1-iodo-4-(trifluoromethyl)benzene **27** (720 µL, 4.87 mmol) in DMF (3 mL). Purification by flash column chromatography using cyclohexane/ethyl acetate (8/2) as the eluent afforded compound **32** (678 mg, 70% yield) as a colorless oil. *R*_f_ 0.29 (cyHex/CH_2_Cl_2_ 8:2); ^1^H NMR (400 MHz, CDCl_3_) δ 7.58 (d, *J* = 8.5 Hz, 2H, H_3′,5′_), 7.08 (d, *J* = 8.5 Hz, 2H, H_2′,6′_), 6.30 (t, *J* = 2.2 Hz, 1H, H_2_), 6.21 (d, *J* = 2.2 Hz, 2H, H_4,6_), 3.77 (s, 6H, 2CH_3_); ^13^C NMR (100 MHz, CDCl_3_) δ 161.9 (2C_1,3_), 160.1 (C_5_), 157.6 (C_1′_), 127.1 (q, *J_CF_* = 4 Hz, 2C_3′,5′_), 125.2 (q, *J_CF_* = 310 Hz, CF_3_), 122.8 (C_4′_), 118.2 (2C_2′,6′_), 98.3 (2C_4,6_), 96.6 (C_2_), 55.5 (2CH_3_); IR (cm^−1^): 2943, 1589, 1321, 1105, 835; MS (ESI) *m*/*z* (%): 299.1 (100) [M + H]^+^; UPLC purity = 99%, *t*_R_ 3.42 min.

#### 3.1.5. General Procedure for the Preparation of 1,3-dimethoxydibenzofurans **33**–**37**

Diaryl ethers **28**–**32** (1 equiv.) were dissolved in warm pivalic acid (50 °C, excess). Silver acetate (5 equiv.) and palladium (II) acetate (0.1 equiv.) were added, and the reaction mixture was properly stirred at 130 °C for 4–24 h. After cooling to room temperature, the mixture was diluted with methanol (50 mL) and NaOH (1 M) was added until the pH reached 9. Elemental silver was removed by filtration through a pad of Celite^®^. The filtrate was extracted with ethyl acetate (100 mL). The organic layers were washed with brine (2 × 100 mL), dried over sodium sulfate, filtered, and evaporated to dryness. The crude product was then purified by chromatography to provide **33**–**37**.

##### 1-(3,7,9-Trimethoxydibenzo[*b*,*d*]furan-2-yl)ethanone (**33**)

The reaction was carried out using 1-[4-(3,5-dimethoxyphenoxy)-2-methoxyphenyl]ethanone **28** (100 mg, 0.33 mmol) and AgOAc (275 mg, 1.65 mmol), Pd(OAc)_2_ (9 mg, 0.03 mmol) in PivOH (5 g) for 4 h. Purification by flash column chromatography using cyclohexane/ethyl acetate (8/2) as the eluent afforded compound **33** (86 mg, 87% yield) as a brown powder. *R*_f_ 0.48 (cyHex/AcOEt 8:2); Mp: 155–156 °C; ^1^H NMR (400 MHz, DMSO-*d*_6_) δ 8.10 (s, 1H, H_1_), 7.46 (s, 1H, H_4_), 6.92 (d, *J* = 1.9 Hz, 1H, H_6_), 6.57 (d, *J* = 1.9 Hz, 1H, H_8_), 3.99 (s, 3H, CH_3_), 3.98 (s, 3H, CH_3_), 3.86 (s, 3H, CH_3_), 2.58 (s, 3H, COCH_3_); ^13^C NMR (100 MHz, DMSO-*d*_6_) δ 198.0 (C=O), 160.6 (C_7_), 158.5 (C_3_), 158.0 (C), 157.9 (C), 154.9 (C_9_), 124.1 (C_2_), 121.9 (C_1_), 115.9 (C), 105.4 (C), 95.8 (C_8_), 94.5 (C_6_), 89.2 (C_4_), 56.3 (CH_3_), 55.9 (CH_3_), 55.8 (CH_3_), 31.6 (CH_3_); IR (cm^−1^): 1664, 1600, 1462, 1408, 1354, 1269, 1236, 1139, 1095; MS (ESI) *m*/*z* (%): 301.2 (100) [M + H]^+^; UPLC purity = 99%, *t*_R_ 3.00 min.

##### 1,3,7-Trimethoxydibenzo[*b*,*d*]furan (**34**)

The reaction was carried out using 1,3-dimethoxy-5-(3-methoxyphenoxy)benzene **29** (100 mg, 0.38 mmol), AgOAc (317 mg, 1.90 mmol), and Pd(OAc)_2_ (9 mg, 0.04 mmol) in PivOH (5 g) for 12 h. Purification by flash column chromatography using cyclohexane/ethyl acetate (8/2) as the eluent afforded compound **34** (90 mg, 91% yield) as a brown powder. *R*_f_ 0.51 (cyHex/AcOEt 8:2); Mp: 153–154 °C; ^1^H NMR (400 MHz, CDCl_3_) δ 7.78 (d, *J* = 8.4 Hz, 1H, H_9_), 6.97 (d, *J* = 2.2 Hz, 1H, H_6_), 6.82 (dd, *J* = 8.4, 2.3 Hz, 1H, H_8_), 6.61 (d, *J* = 1.9 Hz, 1H, H_4_), 6.32 (d, *J* = 2.0 Hz, 1H, H_2_), 3.91 (s, 3H, CH_3_), 3.80 (s, 3H, CH_3_), 3.80 (s, 3H, CH_3_); ^13^C NMR (100 MHz, CDCl_3_) δ 160.0 (C_3_), 158.2 (C_7_), 158.1 (C), 156.6 (C), 155.2 (C_1_), 121.8 (C_9_), 117.0 (C), 110.4 (C_8_), 107.2 (C), 96.4 (C_2_), 93.8 (C_4_), 88.7 (C_6_), 55.8 (CH_3_), 55.7 (CH_3_), 55.6 (CH_3_); IR (cm^−1^): 2940, 2832, 1594, 1435, 1365, 1320, 1264, 1206, 1125, 1036; MS (ESI) *m*/*z* (%): 259.1 (100) [M + H]^+^; UPLC purity = 99%, *t*_R_ 3.24 min.

##### 1-(7,9-Dimethoxydibenzo[*b*,*d*]furan-2-yl)ethanone (**35**)

The reaction was carried out using 1-[4-(3,5-dimethoxyphenoxy)phenyl]ethanone **30** (1.00 g, 3.7 mmol), AgOAc (3.06 g, 18.5 mmol), and Pd(OAc)_2_ (80 mg, 0.37 mmol) in PivOH (50 g) for 24 h. Purification by flash column chromatography using cyclohexane/ethyl acetate (8/2) as the eluent afforded compound **35** (0.83 g, 83% yield) as a brown powder. *R*_f_ 0.34 (cyHex/AcOEt 8:2); Mp: 162–163 °C; ^1^H NMR (400 MHz, CDCl_3_) δ 8.60 (d, *J* = 1.9 Hz, 1H, H_1_), 8.02 (dd, *J* = 8.6, 2.0 Hz, 1H, H_3_), 7.52 (d, *J* = 8.6 Hz, 1H, H_4_), 6.74 (d, *J* = 1.9 Hz, 1H, H_6_), 6.46 (d, *J* = 1.9 Hz, 1H, H_8_), 4.06 (s, 3H, CH_3_), 3.92 (s, 3H, CH_3_), 2.73 (s, 3H, COCH_3_); ^13^C NMR (100 MHz, CDCl_3_) δ 197.6 (C=O), 161.6 (C_7_), 158.8 (C), 158.3 (C_9_), 156.1 (C), 132.7 (C_2_), 125.8 (C_3_), 124.1 (C), 122.6 (C_1_), 110.6 (C_4_), 106.7 (C), 94.4 (C_8_), 88.6 (C_6_), 55.8 (CH_3_), 55.7 (CH_3_), 26.8 (COCH_3_); IR (cm^−1^): 1674, 1637, 1618, 1581, 1510, 1425, 1408, 1361, 1315, 1290, 1253, 1215, 1197, 1151, 1093, 1045, 1010; MS (ESI) *m*/*z* (%): 271.2 (100) [M + H]^+^; UPLC purity = 98%, *t*_R_ 2.94 min.

##### 8-Fluoro-1,3-dimethoxydibenzo[*b*,*d*]furan (**36**)

The reaction was carried out using 1-(4-fluorophenoxy)-3,5-dimethoxybenzene **31** (1.00 g, 4.03 mmol), AgOAc (3.36 g, 20.15 mmol), and Pd(OAc)_2_ (90 mg, 0.40 mmol) in PivOH (50 g) for 24 h. Purification by flash column chromatography using cyclohexane/dichloromethane (8/2) as the eluent afforded compound **36** (414 mg, 42% yield) as a white powder. *R*_f_ 0.23 (cyHex/CH_2_Cl_2_ 8:2); Mp: 137–138 °C; ^1^H NMR (400 MHz, CDCl_3_) δ 7.66 (dd, *J* = 8.5, 2.7 Hz, 1H, H_9_), 7.39 (dd, *J* = 8.9, 4.1 Hz, 1H, H_6_), 7.02 (dt, *J* = 8.9, 2.7 Hz, 1H, H_7_), 6.68 (d, *J* = 2.0 Hz, 1H, H_2_), 6.39 (d, *J* = 2.0 Hz, 1H, H_4_), 4.00 (s, 3H, CH_3_), 3.89 (s, 3H, CH_3_); ^13^C NMR (100 MHz, CDCl_3_) δ 161.6 (C_3_), 159.2 (C_4a_), 159.1 (d, *J_CF_* = 238 Hz, C_8_), 156.0 (C_1_), 151.5 (C_9b_), 124.7 (d, *J_CF_* = 11 Hz, C_9a_), 111.7 (d, *J_CF_* = 26 Hz, C_7_), 111.0 (d, *J_CF_* = 9 Hz, C_6_), 107.9 (d, *J_CF_* = 25 Hz, C_9_), 107.2 (d, *J_CF_* = 3 Hz, C_5a_), 93.9 (C_4_), 88.5 (C_2_), 55.8 (CH_3_), 55.7 (CH_3_); IR (cm^−1^): 1674, 1637, 1618, 1581, 1510, 1425, 1408, 1361, 1315, 1290, 1253, 1215, 1197, 1151, 1093, 1045, 1010; MS (ESI) *m*/*z* (%): 247.1 (100) [M + H]^+^; UPLC purity = 99%, *t*_R_ 3.23 min.

##### 1,3-Dimethoxy-8-(trifluoromethyl)dibenzo[*b*,*d*]furan (**37**)

The reaction was carried out using 1,3-dimethoxy-5-(4-(trifluoromethyl)phenoxy)benzene **32** (500 mg, 1.65 mmol), AgOAc (560 mg, 8.38 mmol), and Pd(OAc)_2_ (37 mg, 0.165 mmol) in PivOH (25 g) for 24 h. Purification by flash column chromatography using cyclohexane/dichloromethane (95/5) as the eluent afforded compound **37** (190 mg, 39% yield) as a white powder. *R*_f_ 0.21 (cyHex/CH_2_Cl_2_ 99:1); Mp: 131–132 °C; ^1^H NMR (400 MHz, CDCl_3_) δ 8.25 (s, 1H, H_9_), 7.58 (dd, *J* = 8.4, 1.9 Hz, 1H, H_7_), 7.53 (d, *J* = 8.7 Hz, 1H, H_6_), 6.72 (d, *J* = 2.0 Hz, 1H, H_2_), 6.43 (d, *J* = 2.0 Hz, 1H, H_4_), 4.03 (s, 3H, CH_3_), 3.90 (s, 3H, CH_3_); ^13^C NMR (100 MHz, CDCl_3_) δ 161.9 (C_3_), 158.9 (C_4a_), 157.0 (C_9b_), 156.1 (C_1_), 126.1 (C_8_), 125.3 (q, *J_CF_* = 310 Hz, CF_3_), 124.2 (C_9a_), 121.9 (q, *J_CF_* = 4 Hz, C_7_), 119.2 (q, *J_CF_* = 4 Hz, C_9_), 110.8 (C_6_), 106.5 (C_5a_), 94.4 (C_4_), 88.6 (C_2_), 55.9 (CH_3_), 55.7 (CH_3_); IR (cm^−1^): 2947, 1620, 1321, 1151, 1094, 808; MS (ESI) *m*/*z* (%): 297.1 (100) [M + H]^+^; UPLC purity = 100%, *t*_R_ 3.47 min.

#### 3.1.6. General Procedure for the Preparation of 1,3-dimethoxydibenzofuran-4-carboxamides **38**–**42**

To a stirred suspension of 1,3-dimethoxydibenzofurans **33**–**37** (1 equiv.) in acetonitrile at 0 °C under argon, chlorosulfonyl isocyanate (2 equiv.) was added. The mixture was maintained at room temperature for 6-12 h and then the reaction mixture was quenched with HCl 5 M (20 mL). After 6–12 h at room temperature, the reaction was diluted with water (100 mL) and extracted with ethyl acetate (100 mL). The combined organic extracts were washed with brine (2 × 100 mL), dried over sodium sulfate, filtered, and evaporated to dryness. The crude product was then purified by chromatography to provide **38**–**42**.

##### 8-Acetyl-1,3,7-trimethoxydibenzo[*b*,*d*]furan-4-carboxamide (**38**)

Method A: The reaction was carried out using 1-(3,7,9-trimethoxydibenzo[*b*,*d*]furan-2-yl)ethanone **33** (100 mg, 0.33 mmol), CSI (58 µL, 0.66 mmol) in CH_3_CN (10 mL) for 12 h and then 12 h for the hydrolysis step at 60 °C, as an exception. Purification by flash column chromatography using cyclohexane/ethyl acetate (6/4) as the eluent afforded compound **38** (54 mg, 48% yield) as a white powder.

Method B: Anhydrous AlCl_3_ (111 mg, 0.83 mmol) was added to acetyl chloride (24 µL, 0.33 mmol) in 1,2-dichloroethane (10 mL). Then, 1,3,7-trimethoxydibenzo[*b*,*d*]furan-4-carboxamide **39** (see below) in 1,2-dichloroethane (20 mL) was added slowly and the mixture was left at room temperature for 1 h. After the reaction (TLC control) was completed, the reaction mixture was quenched with 1 N HCl and extracted with dichloromethane (2 × 25 mL). The combined organic extracts were washed with water, dried over anhydrous sodium sulfate, filtered, and concentrated *in vacuum*. The crude product obtained was purified over silica gel column chromatography using cyclohexane/ethyl acetate (6/4) as the eluent to obtain the pure product **38** (42 mg, 74% yield).

*R*_f_ 0.59 (CH_2_Cl_2_/MeOH 9:1); Mp: 228–229 °C; ^1^H NMR (400 MHz, DMSO-*d*_6_) δ 8.13 (s, 1H, H_9_), 7.70 (s, 1H, CONH_2_), 7.54 (m, 2H, CONH_2_, H_6_), 6.72 (s, 1H, H_2_), 4.08 (s, 3H, CH_3_), 3.97 (s, 3H, CH_3_), 3.93 (s, 3H, CH_3_), 2.58 (s, 3H, COCH_3_); ^13^C NMR (100 MHz, DMSO-*d*_6_) δ 197.9 (COCH_3_), 164.2 (CONH_2_), 158.6 (C), 158.3 (C_7_), 157.1 (C), 155.1 (C_1_), 154.9 (C_3_), 124.2 (C_8_), 122.1 (C_9_), 115.4 (C), 105.5 (C), 105.0 (C_4_), 96.0 (C_6_), 91.6 (C_2_), 56.7 (CH_3_), 56.4 (CH_3_), 56.2 (CH_3_), 31.6 (COCH_3_); IR (cm^−1^): 3387, 3197, 1606, 1402, 1317, 1273, 1089; MS (ESI) *m*/*z* (%): 344.2 (100) [M + H]^+^; UPLC purity = 98%, *t*_R_ 2.15 min; HRMS (TOF MS ES+): calcd. for C_18_H_18_NO_6_ [M + H]^+^ 344.112864, found: 344.11210.

##### 1,3,7-Trimethoxydibenzo[*b*,*d*]furan-4-carboxamide (**39**)

The reaction was carried out using 1,3,7-trimethoxydibenzo[*b*,*d*]furan **34** (100 mg, 0.38 mmol) and CSI (67 µL, 0.77 mmol) in CH_3_CN (10 mL) for 6 h and then 6 h for the hydrolysis step. Purification by flash column chromatography using cyclohexane/ethyl acetate (6/4) as the eluent afforded compound **39** (50 mg, 44% yield) as a white powder. *R*_f_ 0.67 (CH_2_Cl_2_/MeOH 9:1); Mp: 332–333 °C; ^1^H NMR (400 MHz, DMSO-*d*_6_) δ 7.79 (d, *J* = 8.5 Hz, 1H, H_9_), 7.67 (s, 1H, CONH_2_), 7.48 (s, 1H, CONH_2_), 7.30 (d, *J* = 2.3 Hz, 1H, H_6_), 6.95 (dd, *J* = 8.5, 2.3 Hz, 1H, H_8_), 6.69 (s, 1H, H_2_), 4.07 (s, 3H, CH_3_), 3.92 (s, 3H, CH_3_), 3.84 (s, 3H, CH_3_); ^13^C NMR (100 MHz, DMSO-*d*_6_) δ 164.5 (C=O), 158.2 (C_7_), 156.5 (C), 156.1 (C), 154.8 (C_1_), 154.4 (C_3_), 121.4 (C_9_), 115.5 (C), 110.8 (C_8_), 106.1 (C_4_), 104.9 (C), 96.7 (C_6_), 91.2 (C_2_), 56.7 (CH_3_), 56.0 (CH_3_), 55.6 (CH_3_); IR (cm^−1^): 3377, 3192, 2970, 2839, 1643, 1610, 1454, 1431, 1375, 1321, 1267, 1209, 1107, 1026; MS (ESI) *m*/*z* (%): 302.1 (100) [M + H]^+^; UPLC purity = 99%, *t*_R_ 2.26 min; HRMS (TOF MS ES+): calcd. for C_16_H_16_NO_5_ [M + H]^+^ 302.102299, found: 302.10192.

##### 8-Acetyl-1,3-dimethoxydibenzo[*b*,*d*]furan-4-carboxamide (**40**)

The reaction was carried out using 1-(7,9-dimethoxydibenzo[*b*,*d*]furan-2-yl)ethanone **35** (200 mg, 0.74 mmol) and CSI (128 µL, 1.48 mmol) in CH_3_CN (20 mL) for 12 h and then 12 h for the hydrolysis step. Purification by flash column chromatography using cyclohexane/ethyl acetate (6/4) as the eluent afforded compound **40** (74 mg, 32% yield) as a yellow powder. *R*_f_ 0.61 (CH_2_Cl_2_/MeOH 9:1); Mp: 237-238 °C; ^1^H NMR (400 MHz, DMSO-*d*_6_) δ 8.48 (s, 1H, H_9_), 8.07 (d, *J* = 8.8 Hz, 1H, H_7_), 7.90–7.43 (m, 3H, H_6_, CONH_2_), 6.78 (s, 1H, H_2_), 4.14 (s, 3H, CH_3_), 3.96 (s, 3H, CH_3_), 2.68 (s, 3H, CH_3_); ^13^C NMR (100 MHz, DMSO-*d*_6_) δ 197.0 (COCH_3_), 164.1 (CONH_2_), 158.2 (C_3_), 157.5 (C_1_), 155.9 (C), 155.1 (C), 132.6 (C_8_), 126.6 (C_7_), 122.9 (C), 121.3 (C_9_), 110.9 (C_6_), 105.3 (C_4_), 104.8 (C), 91.6 (C_2_), 56.7 (CH_3_), 56.3 (CH_3_), 26.8 (COCH_3_); IR (cm^−1^): 3381, 1649, 1612, 1415, 1365, 1321, 1286, 1215, 1097; MS (ESI) *m*/*z* (%): 314.2 (100) [M + H]^+^; UPLC purity = 100%, *t*_R_ 1.99 min; HRMS (TOF MS ES+): calcd. for C_17_H_16_NO_5_ [M + H]^+^ 314.102299, found: 314.10187.

##### 8-Fluoro-1,3-dimethoxydibenzo[*b*,*d*]furan-4-carboxamide (**41**)

The reaction was carried out using 8-fluoro-1,3-dimethoxydibenzo[*b*,*d*]furan **36** (185 mg, 0.75 mmol) and CSI (130 µL, 1.5 mmol) in CH_3_CN (20 mL) for 12 h and then 12 h for the hydrolysis step. Purification by reverse phase column chromatography (C_18_) using water/acetonitrile (7/3) as the eluent afforded, after lyophilization, compound **41** (76 mg, 35% yield) as a white powder. *R*_f_ 0.26 (AcOEt); Mp: 230–231 °C; ^1^H NMR (400 MHz, DMSO-*d*_6_) δ 7.71–7.67 (s, 1H, NH), 7.68 (dd, *J* = 8.9, 4.1 Hz, 1H, H_6_), 7.64 (dd, *J* = 8.4, 2.7 Hz, 1H, H_9_), 7.51 (s, 1H, NH), 7.24 (dt, *J* = 8.9, 2.7 Hz, 1H, H_7_), 6.72 (s, 1H, H_2_), 4.09 (s, 3H, CH_3_), 3.95 (s, 3H, CH_3_); ^13^C NMR (100 MHz, DMSO-*d*_6_) δ 164.3 (C=O), 158.5 (d, *J_CF_* = 235 Hz, C_8_), 158.2 (C_3_), 155.8 (C_1_), 155.5 (C_4a_), 151.1 (C_9b_), 123.6 (d, *J_CF_* = 11 Hz, C_9a_), 112.4 (d, *J_CF_* = 26 Hz, C_7_), 112.1 (d, *J_CF_* = 9 Hz, C_6_), 107.1 (d, *J_CF_* = 26 Hz, C_9_), 105.8 (C_5a_), 104.7 (C_4_), 91.2 (C_2_), 56.7 (CH_3_), 56.2 (CH_3_); IR (cm^−1^): 3381, 1649, 1612, 1415, 1365, 1321, 1286, 1215, 1097; MS (ESI) *m*/*z* (%): 290.1 (100) [M + H]^+^; UPLC purity = 99%, *t*_R_ 2.23 min; HRMS (TOF MS ES+): calcd. for C_15_H_13_FNO_4_ [M + H]^+^ 290.075037, found: 290.07422.

##### 1,3-Dimethoxy-8-(trifluoromethyl)dibenzo[*b*,*d*]furan-4-carboxamide (**42**)

The reaction was carried out using 1,3-dimethoxy-8-(trifluoromethyl)dibenzo[*b*,*d*]furan **37** (145 mg, 0.50 mmol) and CSI (85 µL, 1.0 mmol) in CH_3_CN (15 mL) for 12 h then 12 h for the hydrolysis step. Purification by flash column chromatography using ethyl acetate/methanol (95/5) as the eluent afforded compound **42** (58 mg, 35% yield) as a white powder. *R*_f_ 0.30 (AcOEt); Mp: 250–251 °C; ^1^H NMR (400 MHz, DMSO-*d*_6_) δ 8.22 (d, *J* = 1.8 Hz, 1H, H_9_), 7.90 (d, *J* = 8.7 Hz, 1H, H_6_), 7.78 (dd, *J* = 8.7, 1.8 Hz, 1H, H_7_), 7.74 (s, 1H, NH), 7.59 (s, 1H, NH), 6.81 (s, 1H, H_2_), 4.17 (s, 3H, CH_3_), 4.02 (s, 3H, CH_3_); ^13^C NMR (100 MHz, DMSO-*d*_6_) δ 164.1 (C=O), 158.6 (C_3_), 156.6 (C_4a_), 156.1 (C_1_), 155.3 (C_9b_), 125.9 (C_8_), 124.2 (q, *J_CF_* = 310 Hz, CF_3_), 123.2 (C_9a_), 122.5 (q, *J_CF_* = 4 Hz, C_7_), 118.1 (q, *J_CF_* = 4 Hz, C_9_), 111.8 (C_6_), 105.0 (C_5a_), 104.6 (C_4_), 91.6 (C_2_), 56.7 (CH_3_), 56.3 (CH_3_); IR (cm^−1^): IR: 3379, 3179, 2974, 1649, 1618, 1319, 1115, 814; MS (ESI) *m*/*z* (%): 340.1 (100) [M + H]^+^; UPLC purity = 98%, *t*_R_ 2.64 min; HRMS (TOF MS ES+): calcd. for C_16_H_13_F_3_NO_4_ [M + H]^+^ 340.071843, found: 340.07112.

#### 3.1.7. General Procedure for the Preparation of 1,3-hydroxydibenzofuran-4-carboxamide **43**–**47**

A mixture of 1,3-dimethoxydibenzofuran-4-carboxamides **38**-**42** (50 mg) in warm pyridine hydrochloride (3g) was irradiated using microwave (100 W) heating at 200 °C for 15 min. After the addition of water, the mixture was extracted with ethyl acetate (100 mL). The organic layers were washed with brine, dried over sodium sulfate, filtered, and evaporated to dryness. The crude product was then purified by chromatography to provide **43**–**47**.

##### 8-Acetyl-1,3,7-trihydroxydibenzo[*b*,*d*]furan-4-carboxamide (**43**)

The reaction was carried out using 8-acetyl-1,3,7-trimethoxydibenzo[*b*,*d*]furan-4-carboxamide **38** (50 mg, 0.15 mmol) in warm pyridine hydrochloride (3g). Purification by flash column chromatography using dichloromethane as the eluent afforded compound **43** (17 mg, 39% yield) as a brown powder. *R*_f_ 0.53 (CH_2_Cl_2_/MeOH 9:1); Mp: 335–336 °C; ^1^H NMR (400 MHz, DMSO-*d*_6_) δ 13.82 (s, 1H, OH), 12.44 (s, 1H, OH), 11.38 (s, 1H, OH), 8.30 (m, 2H, H_9_, CONH_2_), 7.81 (s, 1H, CONH_2_), 7.25 (s, 1H, H_6_), 6.32 (s, 1H, H_2_), 2.74 (s, 3H, COCH_3_); ^13^C NMR (100 MHz, DMSO-*d*_6_) δ 203.7 (COCH_3_), 169.7 (CONH_2_), 163.7 (C_7_), 160.1 (C_3_), 158.8 (C), 157.0 (C_1_), 155.7 (C), 123.3 (C_9_), 117.4 (C), 115.5 (C_8_), 103.5 (C_4_), 99.8 (C_2_), 98.5 (C_6_), 93.0 (C), 27.8 (COCH_3_); IR (cm^−1^): 3462, 3334, 3170, 1614, 1469, 1396, 1246, 1103, 1041; MS (ESI) *m*/*z* (%): 302.1 (100) [M + H]^+^; UPLC purity = 99%, *t*_R_ 2.17 min; HRMS (TOF MS ES+): calcd. for C_15_H_12_NO_6_ [M + H]^+^ 302.065914, found: 302.06557.

##### 1,3,7-Trihydroxydibenzo[*b*,*d*]furan-4-carboxamide (**44**)

The reaction was carried out using 1,3,7-trimethoxydibenzo[*b*,*d*]furan-4-carboxamide **39** (50 mg, 0.17 mmol) in warm pyridine hydrochloride (3g). Purification by flash column chromatography using cyclohexane/ethyl acetate (6/4) as the eluent afforded compound **44** (16 mg, 38% yield) as a brown powder. *R*_f_ 0.67 (CH_2_Cl_2_/MeOH 9:1); Mp: 360–361 °C; ^1^H NMR (400 MHz, DMSO-*d*_6_) δ 13.73 (s, 1H, OH), 11.09 (s, 1H, OH), 9.75 (s, 1H, OH), 8.20 (s, 1H, CONH_2_), 7.80 (s, 1H, CONH_2_), 7.68 (d, *J* = 8.5 Hz, 1H, H_9_), 7.10 (s, 1H, H_6_), 6.81 (d, *J* = 8.3 Hz, 1H, H_8_), 6.25 (s, 1H, H_2_); ^13^C NMR (100 MHz, DMSO-*d*_6_) δ 170.1 (C=O), 162.8 (C_7_), 156.7 (C_3_), 155.7 (C_1_), 155.5 (C), 154.9 (C), 121.1 (C_9_), 114.4 (C), 112.0 (C_8_), 104.7 (C_4_), 98.5 (C_2_), 97.8 (C_6_), 92.7 (C); IR (cm^−1^): 3423, 3321, 3242, 3219, 1664, 1556, 1502, 1446, 1288, 1257, 1190, 1107, 1029; MS (ESI) *m*/*z* (%): 260.1 (100) [M + H]^+^; UPLC purity = 100%, *t*_R_ 1.64 min; HRMS (TOF MS ES+): calcd. for C_13_H_10_NO_5_ [M + H]^+^ 260.055349, found: 260.05591.

##### 8-Acetyl-1,3-dihydroxydibenzo[*b*,*d*]furan-4-carboxamide (**45**)

The reaction was carried out using 8-acetyl-1,3-dimethoxydibenzo[*b*,*d*]furan-4-carboxamide **40** (50 mg, 0.16 mmol) in warm pyridine hydrochloride (3g). Purification by flash column chromatography using cyclohexane/ethyl acetate (6/4) as the eluent afforded compound **45** (12 mg, 26% yield) as a yellow powder. *R*_f_ 0.56 (CH_2_Cl_2_/MeOH 9:1); Mp: 355–356 °C; ^1^H NMR (400 MHz, DMSO-*d*_6_) δ 13.98 (s, 1H, OH), 11.54 (s, 1H, OH), 8.47 (d, *J* = 1.9 Hz, 1H, H_9_), 8.32 (s, 1H, CONH_2_), 8.06 (dd, *J* = 8.6, 2.0 Hz, 1H, H_7_), 7.88 (s, 1H, CONH_2_), 7.81 (d, *J* = 8.6 Hz, 1H, H_6_), 6.36 (s, 1H, H_2_), 2.68 (s, 3H, COCH_3_); ^13^C NMR (100 MHz, DMSO-*d*_6_) δ 197.0 (COCH_3_), 169.8 (CONH_2_), 164.8 (C_3_), 157.8 (C_1_), 156.8 (C), 156.0 (C), 133.0 (C_8_), 125.9 (C_7_), 123.2 (C), 121.0 (C_9_), 111.3 (C_6_), 103.9 (C_4_), 98.4 (C_2_), 92.8 (C), 26.8 (COCH_3_); IR (cm^−1^): 3479, 3190, 1600, 1415, 1359, 1284, 1249, 1190, 1045; MS (ESI) *m*/*z* (%): 286.1 (100) [M + H]^+^; UPLC purity = 99%, *t*_R_ 2.04 min; HRMS (TOF MS ES+): calcd. for C_15_H_12_NO_5_ [M + H]^+^ 286.070999, found: 286.07079.

##### 8-Fluoro-1,3-dihydroxydibenzo[*b*,*d*]furan-4-carboxamide (**46**)

The reaction was carried out using 8-fluoro-1,3-dimethoxydibenzo[*b*,*d*]furan-4-carboxamide **41** (50 mg, 0.17 mmol) in warm pyridine hydrochloride (3g). Purification by flash column chromatography using cyclohexane/ethyl acetate (6/4) as the eluent afforded compound **46** (23 mg, 51% yield) as a beige powder. *R*_f_ 0.39 (cyHex/AcOEt 6:4); Mp: 342–343 °C; ^1^H NMR (400 MHz, DMSO-*d*_6_) δ 13.99 (s, 1H, OH), 11.54 (s, 1H, OH), 8.29 (s, 1H, NH), 7.82 (s, 1H, NH), 7.71 (dd, *J* = 8.8, 4.0 Hz, 1H, H_6_), 7.60 (dd, *J* = 8.4, 2.8 Hz, 1H, H_9_), 7.21 (dt, *J* = 8.8, 2.8 Hz, 1H, H_7_), 6.33 (s, 1H, H_2_); ^13^C NMR (100 MHz, DMSO-*d*_6_) δ 170.0 (C=O), 165.0 (C_3_), 158.8 (d, *J_CF_* = 236 Hz, C_8_), 158.1 (C_1_), 156.4 (C_4a_), 150.4 (C_9b_), 124.0 (d, *J_CF_* = 11 Hz, C_9a_), 112.4 (d, *J_CF_* = 9 Hz, C_6_), 111.7 (d, *J_CF_* = 26 Hz, C_7_), 106.7 (d, *J_CF_* = 26 Hz, C_9_), 104.3 (d, *J_CF_* = 3 Hz, C_5a_), 98.1 (C_4_), 92.6 (C_2_); IR (cm^−1^): 3481, 3188, 3098, 1628, 1474, 1410, 1161, 802; MS (ESI) *m*/*z* (%): 262.1 (100) [M + H]^+^; UPLC purity = 99%, *t*_R_ 2.28 min; HRMS (TOF MS ES+): calcd. for C_13_H_9_FNO_4_ [M + H]^+^ 262.043737, found: 262.04354.

##### 1,3-Dihydroxy-8-(trifluoromethyl)dibenzo[*b*,*d*]furan-4-carboxamide (**47**)

The reaction was carried out using 1,3-dimethoxy-8-(trifluoromethyl)dibenzo[*b*,*d*]furan-4-carboxamide **42** (50 mg, 0.15 mmol) in warm pyridine hydrochloride (3g). Purification by flash column chromatography using cyclohexane/ethyl acetate (6/4) as the eluent afforded compound **47** (24 mg, 53% yield) as a beige powder. *R*_f_ 0.40 (cyHex/AcOEt 6:4); Mp: 347–348 °C; ^1^H NMR (400 MHz, DMSO-*d*_6_) δ 13.99 (s, 1H, OH), 11.98 (s, 1H, OH), 8.33 (s, 1H, NH), 8.14 (d, *J* = 1.6 Hz, 1H, H_9_), 7.90 (d, *J* = 8.8 Hz, 1H, H_6_), 7.87 (s, 1H, NH), 7.74 (dd, *J* = 8.8, 1.6 Hz, 1H, H_7_), 6.53 (s, 1H, H_2_); ^13^C NMR (100 MHz, DMSO-*d*_6_) δ 169.8 (C=O), 165.1 (C_3_), 158.3 (C_1_), 156.1 (C_4a_), 155.9 (C_9b_), 125.9 (C_8_), 124.6 (q, *J_CF_* = 310 Hz, CF_3_), 123.6 (C_9a_), 121.9 (q, *J_CF_* = 4 Hz, C_7_), 117.6 (q, *J_CF_* = 4 Hz, C_9_), 112.3 (C_6_), 103.6 (C_5a_), 98.7 (C_4_), 92.6 (C_2_); IR (cm^−1^): 3474, 3217, 1632, 1308, 1109, 887, 818; MS (ESI) *m*/*z* (%): 312.1 (100) [M + H]^+^; UPLC purity = 99%, *t*_R_ 2.58 min; HRMS (TOF MS ES+): calcd. for C_14_H_9_F_3_NO_4_ [M + H]^+^ 312.040543, found: 312.04098.

#### 3.1.8. Access to Dibenzofuran Derivative 19 by Intramolecular C-O Bond Formation from 2-Aryl Phenol **49**

##### 2-Hydroxy-3-iodo-4,6-dimethoxybenzamide (**48**)

A solution of 2-hydroxy-4,6-dimethoxybenzamide **2** (200 mg, 1.01 mmol) in dichloromethane (10 mL) at 0 °C under argon was added *N*-iodosuccinimide (230 mg, 1.01 mmol) and the reaction mixture was maintained at this temperature for 10 min. The reaction was diluted with water (30 mL) and extracted with dichloromethane (3 × 50 mL). The combined organic extracts were dried over sodium sulfate and filtered. The solvent was evaporated *in vacuo* and the residue was purified by silica gel column chromatography using cyclohexane/ethyl acetate (6/4) as the eluent to give compound **48** as a white powder (196 mg, 60% yield). *R*_f_ 0.21 (cyHex/AcOEt 6:4); Mp: 270−271 °C; ^1^H NMR (400 MHz, DMSO-*d*_6_) δ 15.74 (s, 1H, OH), 8.13 (s, 1H, CONH_2_), 8.13 (s, 1H, CONH_2_), 6.30 (s, 1H, H_5_), 3.97 (s, 3H, CH_3_), 3.92 (s, 3H, CH_3_); ^13^C NMR (100 MHz, DMSO-*d*_6_) δ 171.3 (C=O), 163.9 (C_2_), 162.1 (C_6_), 161.2 (C_4_), 97.1 (C_1_), 87.5 (C_5_), 67.1 (C_3_), 56.5 (CH_3_), 56.4 (CH_3_); IR (cm^−1^): 3439, 1602, 1579, 1415, 1396, 1336, 1286, 1215, 1120, 1078; MS (ESI) *m*/*z* (%): 324.0 (100) [M + H]^+^; UPLC purity = 98%, *t*_R_ 2.25 min.

##### 2’-Fluoro-2-hydroxy-4,6-dimethoxybiphenyl-3-carboxamide (**49**)

2-Hydroxy-3-iodo-4,6-dimethoxybenzamide **48** (1.00 g, 3.1 mmol), 2-fluorophenylboronic acid (650 mg, 4.7 mmol) and potassium phosphate (1.33 g, 6.2 mmol) were added to degassed acetonitrile (16 mL) and water (4 mL). After stirring for 10 min, palladium(II) acetate (35 mg, 0.05 equiv.) and triphenylphosphine (40 mg, 0.05 equiv.) were added to the mixture. The tube was then sealed, and the reaction mixture was irradiated using microwave heating at 90 °C for 2 h. Acetonitrile was removed, the crude product was extracted with ethyl acetate (100 mL) and washed with water (2 × 100 mL), dried over sodium sulfate, filtered, and concentrated under reduced pressure. The crude product was purified by flash silica gel column chromatography using cyclohexane/ethyl acetate (6/4) as the eluent to afford compound **48** (234 mg, 26%) as a brown powder. *R*_f_ 0.41 (CH_2_Cl_2_/MeOH 9:1); Mp: 223–224 °C; ^1^H NMR (400 MHz, DMSO-*d*_6_) δ 14.92 (s, 1H, OH), 8.13 (s, 1H, CONH_2_), 8.03 (s, 1H, CONH_2_), 7.34 (d, *J* = 7.0 Hz, 1H), 7.26-6.95 (m, 3H), 6.32 (s, 1H, H_5_), 4.00 (s, 3H, CH_3_), 3.78 (s, 3H, CH_3_).; ^13^C NMR (100 MHz, DMSO-*d*_6_) δ 171.9 (C=O), 162.5 (C_2_), 160.9 (C_6_), 160.5 (C_4_), 158.8 (d, *J* = 243 Hz, C_2′_-F), 133.3 (d, *J* = 4 Hz, C_6′_), 128.8 (d, *J* = 9 Hz, C_4′_), 123.60 (d, *J* = 4 Hz, C_5′_), 121.18 (d, *J* = 17 Hz, C_1′_), 115.0 (d, *J* = 22 Hz, C_3′_), 104.4 (C_1_), 96.5 (C_3_), 86.8 (C_5_), 56.2 (CH_3_), 55.7 (CH_3_); IR (cm^−1^): 3435, 1649, 1604, 1591, 1415, 1317, 1209, 1138, 1114; MS (ESI) *m*/*z* (%): 292.2 (100) [M + H]^+^; UPLC purity = 98%, *t*_R_ 2.52 min.

##### 1,3-Dimethoxydibenzo[*b*,*d*]furan-4-carboxamide (**19**)

For the cyclisation step, see above, Method B.

### 3.2. Molecular Modeling

Molecular modeling studies were performed using SYBYL-X 1.3 software [62] running on a Dell precision T3400 workstation. The three-dimensional structures of compound **44** (most active compound), compound **43** (strict analogue of cercosporamide), and cercosporamide were built from a standard fragments library and optimized using the Tripos force field [63] including the electrostatic term calculated from Gasteiger and Hückel atomic charges. Powell’s method available in the Maximin2 procedure was used for energy minimization until the gradient value was smaller than 0.001 kcal/(mol*Å). The crystal structure of Pim-1 in complex with AMP-PNP at 1.6 Å resolution (PDB ID 3A99) [64] was used as a template for docking. Water molecules were removed from the coordinates set since no information about conserved water molecules is known for this chemical series in Pim-1. Flexible docking of compounds **44**, **43**, and cercosporamide into the ATP-binding site was performed using GOLD software [65]. The most stable docking models were selected according to the best scored conformation predicted by the ChemScore scoring function implemented in GOLD. Finally, the complexes were energy-minimized using Powell’s method available in the Maximin2 procedure with the Tripos force field and a dielectric constant of 4.0, until the gradient value reached 0.1 kcal/mol.Å. Biovia Discovery Studio Visualizer [66] was used for graphical display.

### 3.3. Biology

#### 3.3.1. Mammalian Protein Kinase Assays

Kinase enzymatic activities were assayed with 10 µM ATP in 384-well plates using the luminescent ADP-Glo^TM^ assay (Promega, Madison, WI, USA) according to the recommendations of the manufacturer (see [56] for details on this method). The transmitted signal was measured using the Envision (PerkinElmer, Waltham, MA, USA) microplate luminometer and expressed in relative light unit (RLU). In order to determine the half maximal inhibitory concentration (IC_50_), the assays were performed in duplicate in the absence or presence of increasing doses of the tested compounds. GraphPad Prism6 software (GraphPad Software, San Diego, CA, USA) was used to fit dose–response curves and to determine the IC_50_ values. Kinase activities are expressed in % of maximal activity, i.e., measured in the absence of inhibitor. Peptide substrates were obtained from Proteogenix (Schiltigheim, France).

The following kinases were analyzed during this study: *Hs*Pim-1 and *Hs*Pim-2 (human proto-oncogene, recombinant, expressed in bacteria) were assayed with 0.20 µg/µL of consensus peptide substrate: ARKRRRHPSGPPTA; *Rn*DYRK1A-kd (*Rattus norvegicus*, amino acids 1 to 499 including the kinase domain, recombinant, expressed in bacteria, DNA vector kindly provided by Dr. W. Becker, Aachen, Germany) was assayed with 0.033 µg/µL of the following peptide: KKISGRLSPIMTEQ as substrate; *Hs*CDK5/p25 (human cyclin-dependent kinase-5, recombinant, expressed in bacteria) was assayed with 0.8 µg/µL of histone H1 as substrate; *Hs*CDK9/CyclinT (human cyclin-dependent kinase-9, recombinant, expressed by baculovirus in Sf9 insect cells) was assayed with 0.20 µg/µL of the following peptide: YSPTSPSYSPTSPSYSPTSPSKKKK, as substrate; *Hs*Haspin-kd (human, kinase domain, amino acids 470 to 798, recombinant, expressed in bacteria) was assayed with 0.007 µg/µL of Histone H3 (1–21) peptide (ARTKQTARKSTGGKAPRKQLA) as substrate; *Mm*CLK1 (from *Mus musculus*, recombinant, expressed in bacteria) was assayed with 0.027 µg/µL of the following peptide: GRSRSRSRSRSR as substrate; *Hs*CK1ε (human casein kinase 1ε, recombinant, expressed by baculovirus in Sf9 insect cells) was assayed with 0.02 µg/µL of the following peptide: RRKHAAIGSpAYSITA (“Sp” stands for phosphorylated serine) as the CK1-specific substrate; *Hs*GSK-3β (human glycogen synthase kinase-3) was assayed with 0.010 µg/µL of GS-1 peptide, a GSK-3-selective substrate (YRRAAVPPSPSLSRHSSPHQSpEDEEE). For kinases expressed in bacteria, the *Escherichia coli* BL21 DE3 pLysS strain (Invitrogen, ThermoFisher Scientific, Waltham, MA, USA) was transformed with pGEX-2T-1 (Sigma-Aldrich, St. Louis, MO, USA) containing the coding region of the corresponding kinase. For kinases expressed in Sf9 insect cells, the coding region of the corresponding kinase was cloned in pFastBac^TM^ vector. Purification of the recombinant kinases was performed following the protocol “Bac-to-Bac^®^ Baculovirus Expression System” provided by the manufacturer (Invitrogen, ThermoFisher Scientific, Waltham, MA, USA).

To validate the kinase assay, model inhibitors were used for each tested enzyme: Staurosporine from *Streptomyces* sp. (#S5921, purity ≥ 95%, Sigma-Aldrich) for *Hs*CK1ε; Indirubin-30-oxime (#I0404, purity ≥ 98%, Sigma-Aldrich) for *Hs*GSK-3β, *Hs*Pim-1 and 2, human cyclin-dependent kinases, *Rn*DYRK1A and *Mm*CLK1; CHR-6494 (#SML0648, purity ≥ 98%, Sigma-Aldrich) for Haspin.

#### 3.3.2. Cell Cultures and Reagents

MV4-11, K562, and KU812 cell lines were obtained from the *Deutshe Sammlung von Mikroorganismens und Zellkulturen* (DSMZ) and maintained according to the supplier’s recommendations. All cell lines were cultured in RPMI, with 10% fetal bovine serum, 1% glutamine, and 1% penicillin/ streptomycin at 37 °C and 5% CO_2_.

The cancer cell lines MCF-7, HT-29, and HeLa were obtained from Cell Bank of Rio de Janeiro, Brazil, and were grown in RPMI-1640 medium and L929 cells were grown in Dulbecco’s Modified Eagle’s Medium (DMEM). Both media were supplemented with fetal bovine serum (FBS) (10% of the final concentration) followed by treatment with penicillin and streptomycin as antibiotics (1% of the final concentration). The crude was incubated in the presence of CO_2_ atmosphere (5%) for 24 h with the temperature monitored and maintained at 37 °C.

#### 3.3.3. In Vitro Cell-Based Assays

MV4-11, K562, and KU812 cells’ viability was studied using a MTT cell proliferation assay. To determine the concentration effect of the molecules, 0.2 × 10^6^ leukemic cells were incubated in 100 μL of RPMI red phenol-free medium (Gibco), in 96-well plates and treated with compounds **15**–**17** and **43**–**47** (stock solution at 50 mM in DMSO) or the positive control **SGI-1776** from SAB Signalway antibody with concentrations ranging from 100 nM to 100 μM for 48 h.

MV4-11, K562, and KU812 cells were incubated with 10 μL of MTT working solution (5 g/L of methylthiazolyldiphenyl-tetrazolium bromide from Sigma Aldrich, Lyon, France) for 4 h. Cells were then lysed overnight at 37 °C with 100 μL of 10% SDS and 0.003% HCl. Optical density (OD) at 570 nm was measured using a spectrophotometer CLARIOstar^®^ (BMG Labtech, Offenburg, Germany). Living cells were also counted with the trypan blue dye exclusion method. When a dose-dependent activity was observed, IC_50_ values were calculated using Graphpad PRISM 7 software (*n* = 3 in triplicate). Data were collected from at least three independent experiments and the values reported are means ± standard errors of the mean (SEM).

MCF-7, HT-29, HeLa, and L929 cells (3×10^5^ cells/mL) were plated in 96-well plates and incubated in the presence of CO_2_ atmosphere (5%) for 24 h at 37 °C. Then, compounds **15**–**17** and **43**–**47** with concentrations ranging from 100 nM to 100 μM were added and incubated at 37 °C for 72 h. Then, 25 μL of MTT (5 mg/mL in PBS) were added to each well and the plates were left for another 3 h in the incubator at 37 °C and at the end of this period, the supernatant was aspirated. To perform the reading, 100 μL of dimethylsulfoxide (DMSO) were added in each well for the dissolution of the formazan crystals. The amount of formazan was measured by reading the plate at 560 nm absorbance. The concentration leading to 50% inhibition of viability (IC_50_) was calculated by regression analysis using GraphPad Prism 5.0. Each sample was tested in triplicate in two independent experiments. Doxorubicin was used as a positive control.

#### 3.3.4. Evaluation of Compounds’ Cytotoxicity Using the In Vivo *Galleria mellonella* Model

*G. mellonella* larvae were bred in the IICiMed laboratory at 30-32 °C in a mixture of flour, honey, oatmeal, glycerol, and pollen until 260 to 320 mg (6^th^ developmental stage). Groups of 10 larvae were randomly selected for the experiments. Each larva was injected in the 4^th^ left pro-leg with 10 µL of compounds or an equivalent volume of PBS/DMSO for the control group at a dose varying between 10 and 50 mg/kg using a 0.3 mL–30G-Insulin syringe. Larvae were incubated in the dark at 37 °C and survival was evaluated daily for 7 days. Death was assessed by the lack of movement in response to stimulus, with or without discoloration. Percentage survival was plotted using GraphPad Prism and survival analyses were determined using the log-rank test and the Kaplan–Meier survival curves. Two independent experiments were performed.

## 4. Conclusions

In summary, our research efforts in the synthesis of a natural product-inspired chemotype, endowed with kinase inhibition properties, produced attractive and safe 1,3-dihydroxydibenzo[*b*,*d*]furan derivatives. This study confirmed cercosporamide as a valuable starting point for medicinal chemistry investigations and the removal of chirality afforded a flat tricyclic core with very promising enzyme and cellular potencies. Indeed, it was well established that the dual Pim1/2-CLK kinases inhibition promoted very interesting antiproliferative properties against the AML cell line as confirmed by the growth inhibitory activity at a low micromolar concentration, coupled with the nanomolar IC_50_ values obtained against the enzymes, particularly for the most active compound **44**.

Docking studies demonstrated that the 4-carboxamide group of the dibenzofurans was not essential for binding within the ATP pocket of Pim-1, in contrast to the hydroxy groups. In future, the replacement of the carboxamide group will be investigated to validate the hypothesis, and taking into account the level of activity of usnic acid towards Pim1/2, its substitution pattern could also serve as model for further design. In parallel, the very encouraging results obtained for this synthetic compound library deserve further optimization for complete SAR studies.

## Figures and Tables

**Table 1:** Kinase selectivity profile of dibenzo[*b*,*d*]furan derivatives (ADP-Glo method at 10 µM ATP).

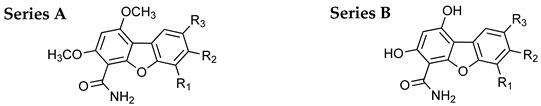									
						Kinase Enzymatic IC_50_ (µM) ^1,2^			
Entry	Compound	Series	R_1_	R_2_	R_3_	CDK5/ p25	CDK9/ CyclinT	Pim-1	CLK1
1	**Cerco ^3^**					5.60	**0.22**	**0.73**	>10
2	**14**	A	NO_2_	H	H	>10	>10	3.21	>10
3	**15**	B	NO_2_	H	H	>10	>10	**0.18**	**1.22**
4	**18**	A	NH_2_	H	H	>10	>10	>10	>10
5	**16**	B	NH_2_	H	H	>10	>10	**0.13**	**0.45**
6	**19**	A	H	H	H	>10	>10	>10	**0.75**
7	**17**	B	H	H	H	>10	>10	**0.23**	**0.26**
8	**38**	A	H	OCH_3_	COCH_3_	>10	>10	>10	>10
9	**43**	B	H	OH	COCH_3_	>10	>10	**0.82**	**0.29**
10	**39**	A	H	OCH_3_	H	>10	>10	>10	>10
11	**44**	B	H	OH	H	>10	>10	**0.06**	**0.026**
12	**40**	A	H	H	COCH_3_	>10	>10	>10	>10
13	**45**	B	H	H	COCH_3_	>10	>10	**0.28**	**0.14**
14	**41**	A	H	H	F	>10	>10	>10	**0.85**
15	**46**	B	H	H	F	>10	0.92	**1.20**	**0.62**
16	**42**	A	H	H	CF_3_	>10	>10	>10	>10
17	**47**	B	H	H	CF_3_	>10	>10	**2.35**	>10

^1^ IC_50_ values were calculated from dose–response curves. Each inhibitor concentration was tested in duplicate. All protein kinases used here are human with the exception of DYRK1A (*Rattus norvegicus*) and CLK1 (*Mus musculus*). DYRK1A: dual specificity tyrosine phosphorylation regulated kinase 1A, CDK: cyclin-dependent kinase, Haspin: haploid germ cell-specific nuclear protein kinase, CLK1: cdc2-like kinase 1, CK1: casein kinase 1, GSK3β: glycogen synthase kinase 3, Pim: Proviral integration site for Moloney murine leukemia virus. ^2^ All the compounds remained inactive against Haspin, DYRK1A, GSK3 and CK1. Except compound **41**: Haspin (0.93 µM) and DYRK1A (1.33 µM) and compound **46**: Haspin (0.80 µM), DYRK1A (0.69 µM), and CK1 (0.68 µM). ^3^ Cercosporamide (**Cerco**) was used as the reference compound.

**Table 2:** Enzymatic assays on human Pim-1 and Pim-2 kinases (ADP-Glo method at 10 µM ATP).

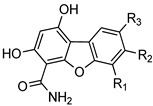						
					IC_50_ (µM) ^1^	
Entry	Compound	R_1_	R_2_	R_3_	Pim-1	Pim-2
1	**Cerco ^2^**				0.73	1.43
2	**15**	NO_2_	H	H	0.18	0.33
3	**16**	NH_2_	H	H	0.13	0.085
4	**17**	H	H	H	0.23	0.20
5	**43**	H	OH	COCH_3_	0.82	0.14
6	**44**	H	OH	H	0.06	0.035
7	**45**	H	H	COCH_3_	0.28	0.12
8	**46**	H	H	F	1.2	0.25
9	**47**	H	H	CF_3_	2.35	0.37

^1^ IC_50_ on Pim-1/2 kinase activity was calculated from dose–response curves. Each inhibitor concentration was tested in duplicate. Values are a mean of *n* ≥ 3 independent experiments. ^2^ Cercosporamide (**Cerco**) was used reference compound.

**Table 3:** Cell-based assays of representative dibenzo[*b*,*d*]furane derivatives.

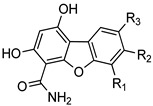										
					IC_50_ (µM) ^1,2^					
Entry	Compound	R_1_	R_2_	R_3_	MV4-11	KU812	K562	MCF-7	HeLa	L929
1	**15**	NO_2_	H	H	30.7 ± 1.1	78.4 ± 2.1	>100	>100	24.7 ± 6.7	49.2 ± 1.0
2	**16**	NH_2_	H	H	10.1 ± 0.8	>100	>100	>100	14.6 ± 7.2	>100
3	**17**	H	H	H	14.3 ± 1.1	>100	>100	>100	9.5 ± 4.1	>100
4	**43**	H	OH	COCH_3_	52.2 ± 1.7	>100	>100	>100	57.1 ± 7.6	>100
5	**44**	H	OH	H	2.6 ± 0.4	42.1 ± 1.3	75.7 ± 11.8	52.5 ± 1.2	10.2 ± 1.2	28.4 ± 1.1
6	**45**	H	H	COCH_3_	8.8 ± 0.9	69.4 ± 12.9	>100	>100	59.6 ± 7.6	>100
7	**46**	H	H	F	16.1 ± 1.6	>100	>100	-	-	-
8	**47**	H	H	CF_3_	>100	>100	>100	-	-	-
9	**Cerco ^3^**				31.5 ± 4.7	>100	>100	44.3 ± 2.1	7.5 ± 3.1	-
10	**Doxo ^3^**				-	-	-	0.50 ± 0.02	2.7 ± 0.2	2.4 ± 0.4
11	**SGI-1776 ^3^**				0.030 ± 0.003	3.5 ± 0.6	3.7 ± 1.5	-	-	-

^1^ IC_50_ HT-29 >100 µM for all the compounds and **Cerco** IC_50_ = 10.4 ± 2.5 µM; **Doxo** IC_50_ = 0.72 ± 0.5 µM. ^2^ Values are a mean of n ≥ 3 independent experiments. Cells were treated with concentrations ranging from 100 nM to 100 µM for 48 h or 72 h (MCF-7, Hela and L929). Cell viability was then determined by MTT assays, and EC_50_ values were calculated using Graphpad PRISM 7 software (*n* = 3 in triplicate; data are in the mean ± SEM). -: Not determined. ^3^ Cercosporamide (**Cerco**) was used as the reference compound. Doxorubicin (**Doxo**) and SGI-1776 were used as positive controls.

## Data Availability

Not applicable.

## Supplementary Materials

Spectroscopic data (^1^H and ^13^C NMR spectra) for all the compounds.

## Sample Availability

Samples of the compounds are available from the authors.
