# Supplementary material for: Dibenzofuran Derivatives Inspired from Cercosporamide as Dual Inhibitors of Pim and CLK1 Kinases
Source: Molecules. 2021 Oct 30;26(21):6572. doi: 10.3390/molecules26216572 (PMC8587151; doi:10.3390/molecules26216572)

molecules

Supplementary Materials

Dibenzofuran derivatives inspired from cercosporamide as dual inhibitors of Pim and CLK1 kinases

Viet Hung Dao [1,†], Isabelle Ourliac-Garnier [1], Cédric Logé [1], Florence O. McCarthy [2], Stéphane Bach [3,4,5], Teresinha Gonçalves da Silva [6], Caroline Denevault-Sabourin [7], Jérôme Thiéfaine [1], Blandine Baratte [3,4], Thomas Robert [3,4], Fabrice Gouilleux [8], Marie Brachet-Botineau [8], Marc-Antoine Bazin [1] and Pascal Marchand [1,*]

[1] Cibles et Médicaments des Infections et du Cancer, IICiMed, EA 1155, Université de Nantes, 44000 Nantes, France; daoviethung@live.com (V.H.D.); isabelle.ourliac@univ-nantes.fr (I.O.-G.); cedric.loge@univ-nantes.fr (C.L.); jerome.thiefaine@univ-nantes.fr (J.T.); marc-antoine.bazin@univ-nantes.fr (M.-A.B.)
[2] School of Chemistry, Analytical and Biological Chemistry Research Facility, University College Cork, Western Road, T12 K8AF Cork, Ireland; f.mccarthy@ucc.ie (F.O.M.)
[3] Sorbonne Université, CNRS, UMR8227, Integrative Biology of Marine Models Laboratory (LBI2M), Station Biologique de Roscoff, 29680, Roscoff, France; bach@sb-roscoff.fr (S.B.); baratte@sb-roscoff.fr (B.B.); thomas.robert@sb-roscoff.fr (T.R.)
[4] Sorbonne Université, CNRS, FR2424, Plateforme de Criblage KISSf (Kinase Inhibitor Specialized Screening Facility), Station Biologique de Roscoff, 29680, Roscoff, France
[5] Centre of Excellence for Pharmaceutical Sciences, North-West University, Private Bag X6001, 2520 Potchefstroom, South Africa
[6] Departamento de Antibióticos, Universidade Federal de Pernambuco, Recife, PE 50670-901, Brazil; teresinha.goncalves@ufpe.br (T.G.d.S.)
[7] EA GICC-ERL 7001 CNRS, Team IMT, University of Tours, 37200 Tours, France; caroline.denevault@univ-tours.fr (C.D.-S.)
[8] CNRS ERL7001 LNOx « Leukemic Niche and redOx Metabolism », EA GICC, University of Tours, 37000 Tours, France; fabrice.gouilleux@univ-tours.fr (F.G.); marie.brachet.botineau@gmail.com (M.B.-B.)
* Correspondence: pascal.marchand@univ-nantes.fr; Tel.: +33-253-009-155
† Current address: Phu Tho College of Medicine and Pharmacy, Viet Tri, Phu Tho 290000, Vietnam.

Spectroscopic data (^{1}H and ^{13}C NMR spectra) for all the compounds

2-Hydroxy-4,6-dimethoxybenzamide (2)

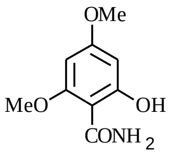

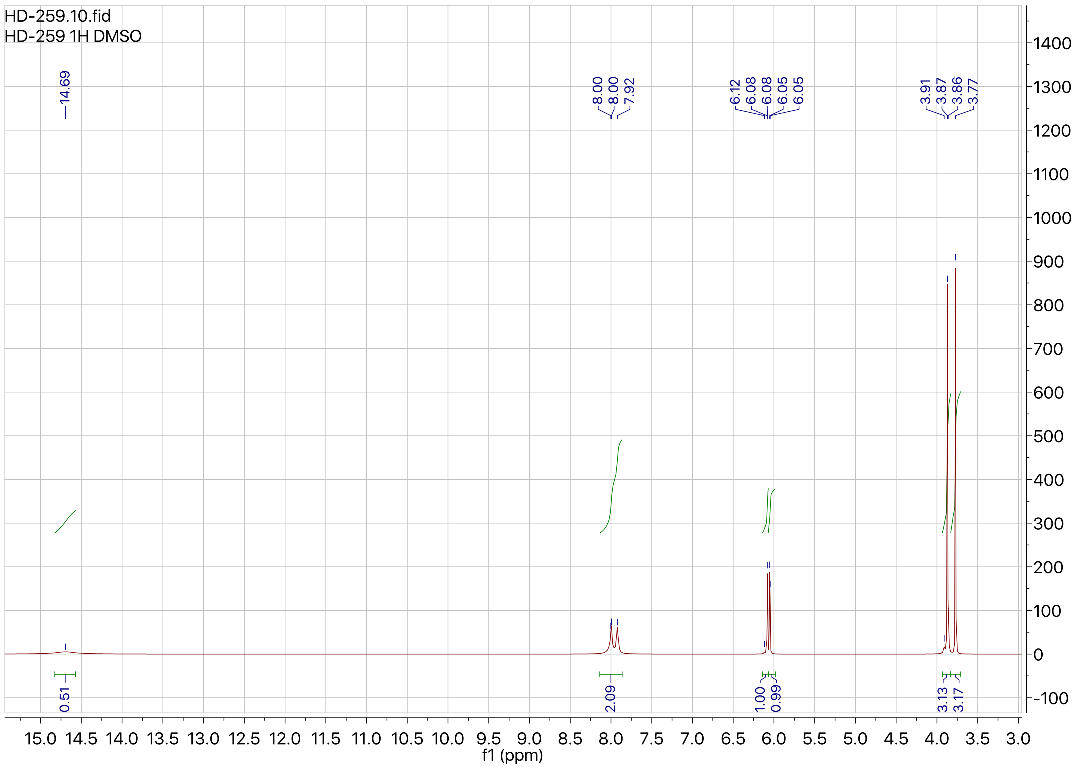

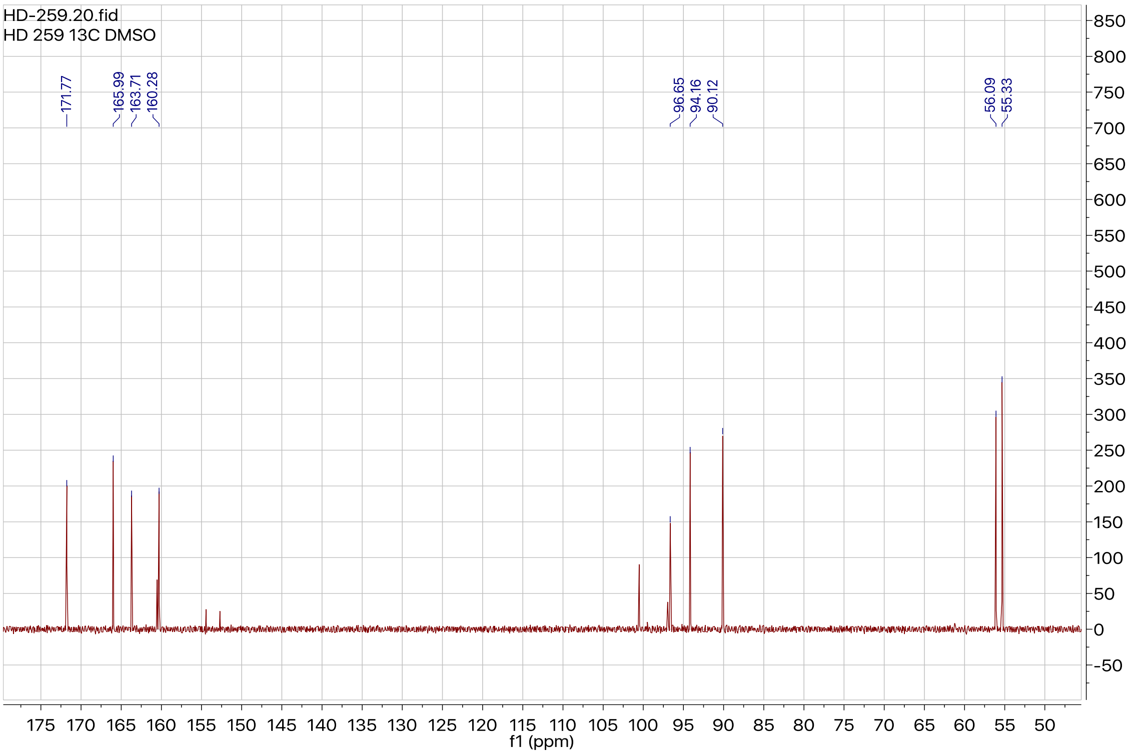

2,4-Dimethoxy-6-(2′-nitrophenoxy)benzamide (6)

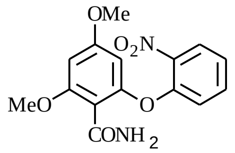

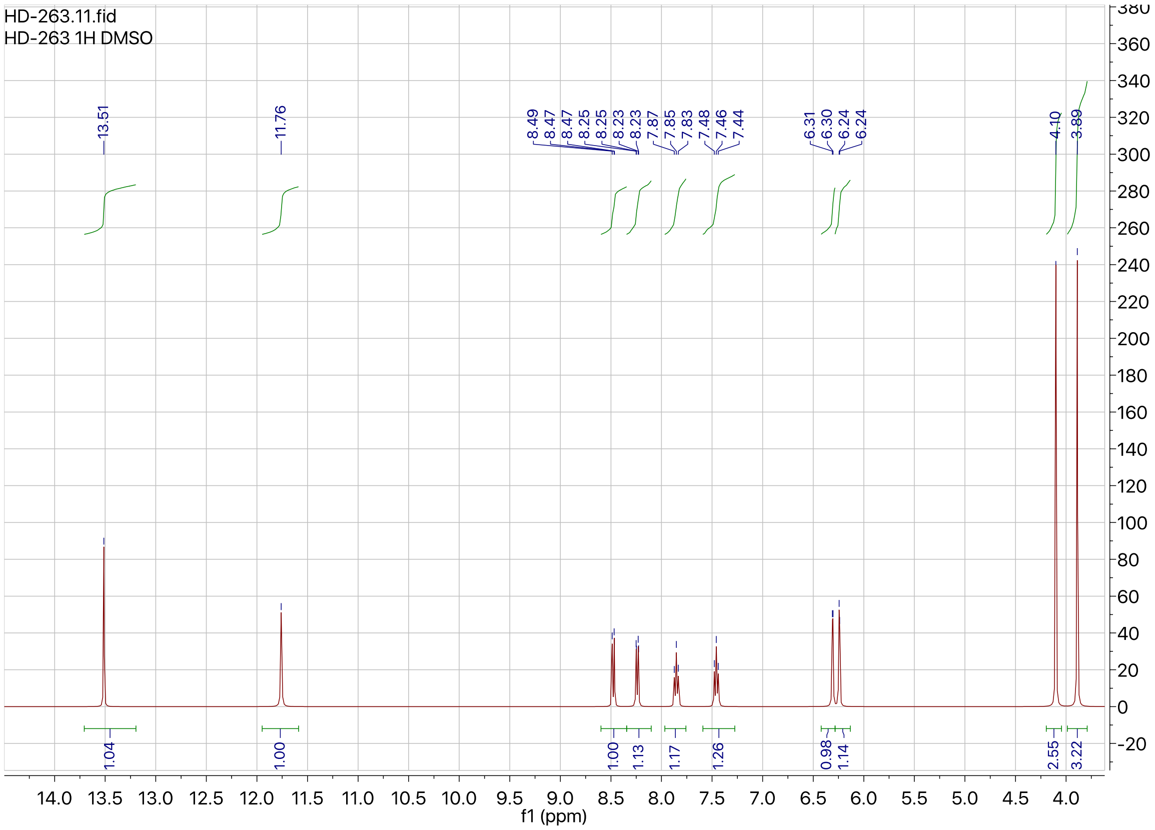

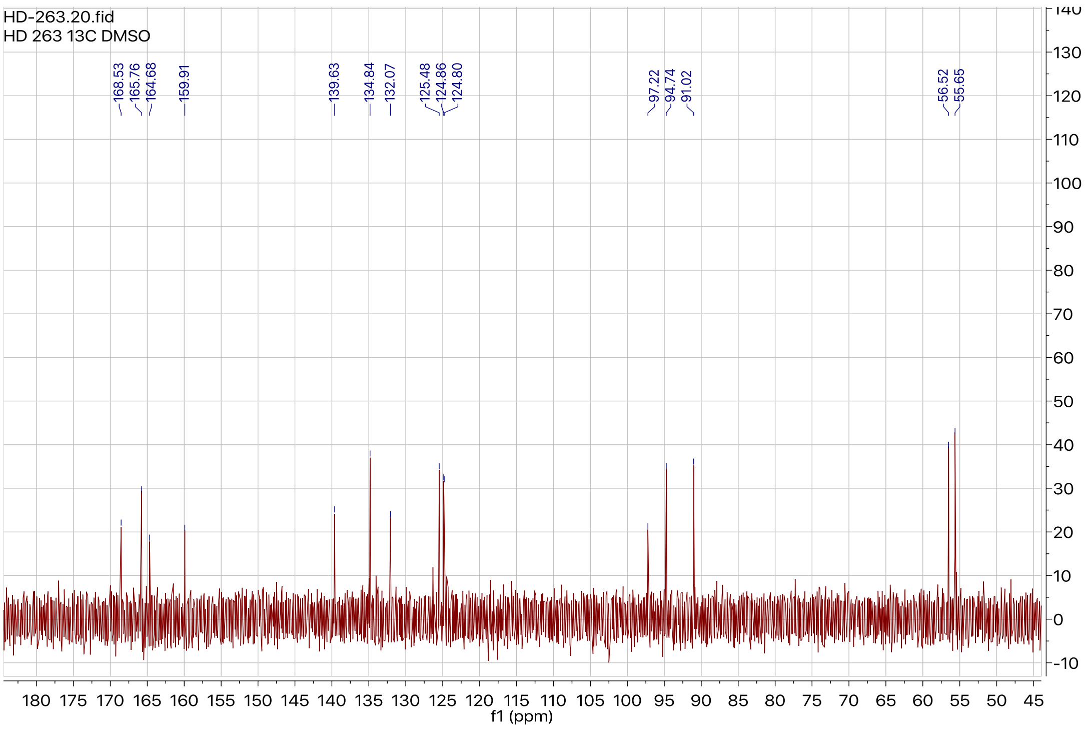

1,3-Dibenzyloxy-5-(2′-nitrophenoxy)benzene (7)

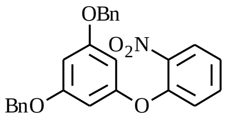

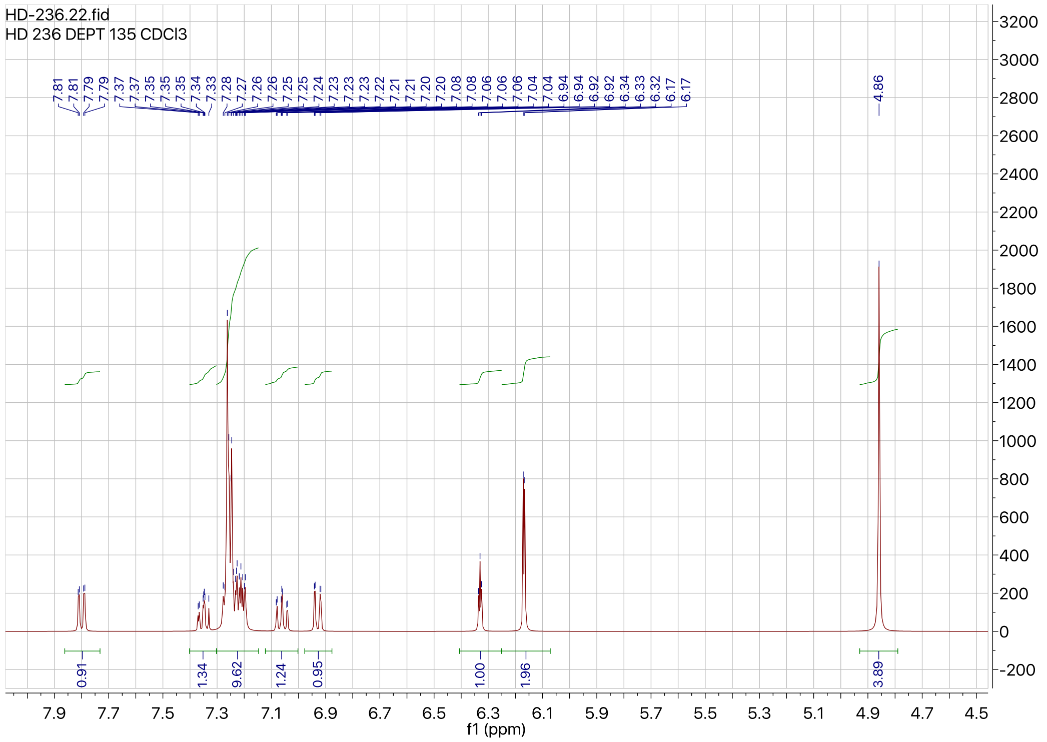

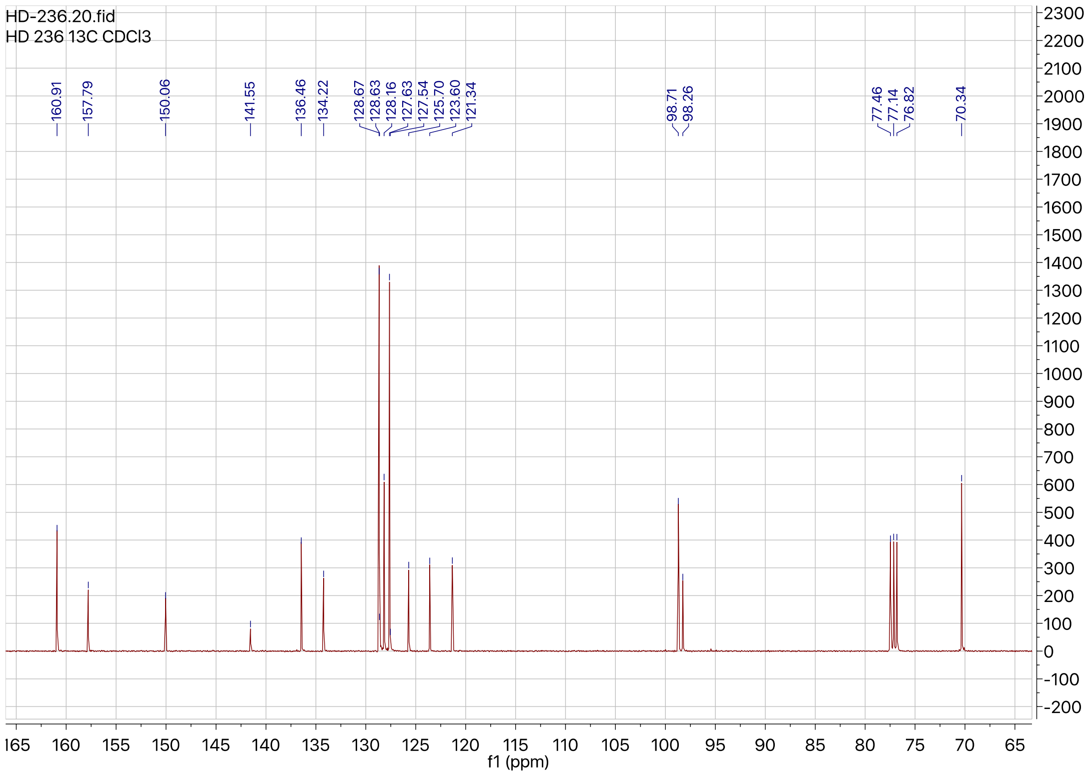

1,3-Dimethoxy-5-(2′-nitrophenoxy)benzene (8)

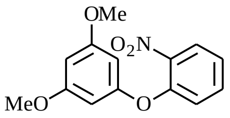

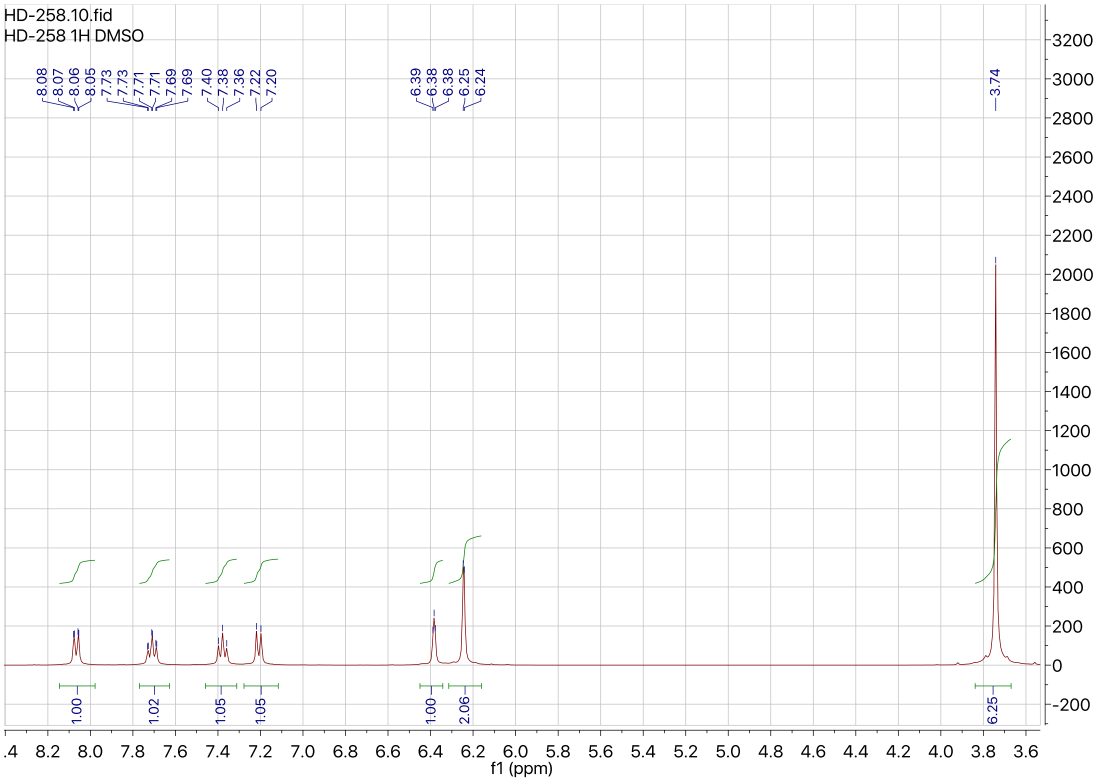

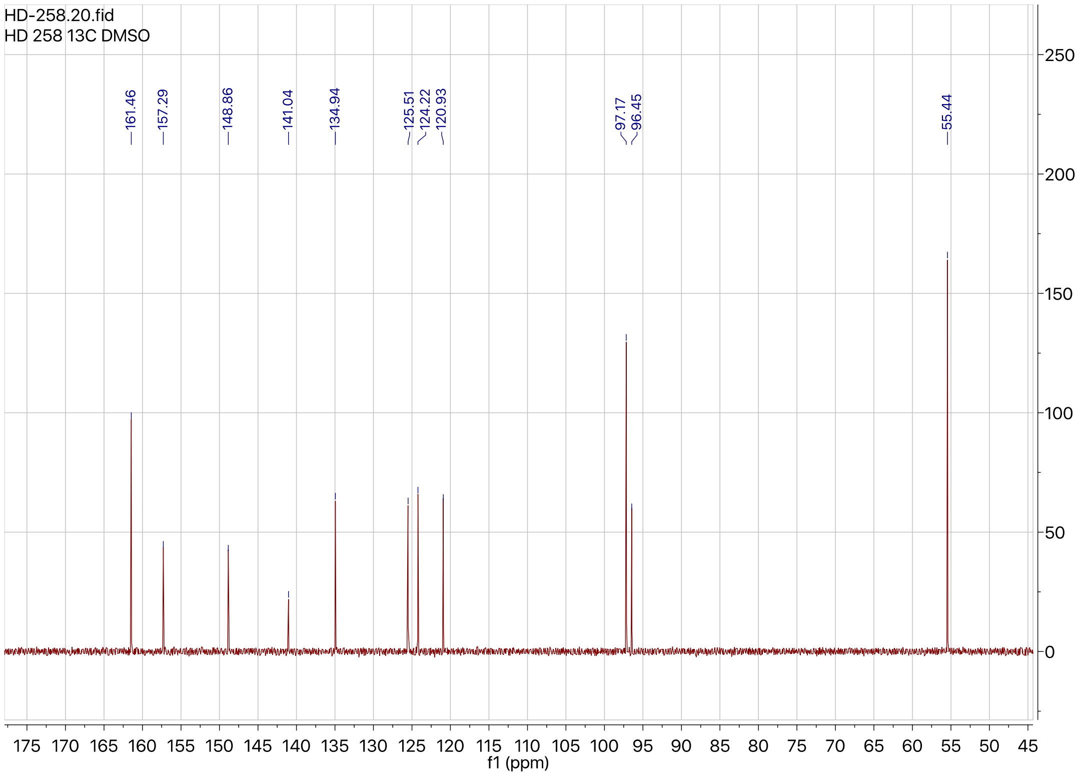

2-(3′,5′-Dibenzyloxyphenoxy)aniline (9)

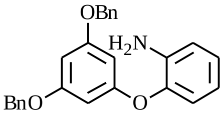

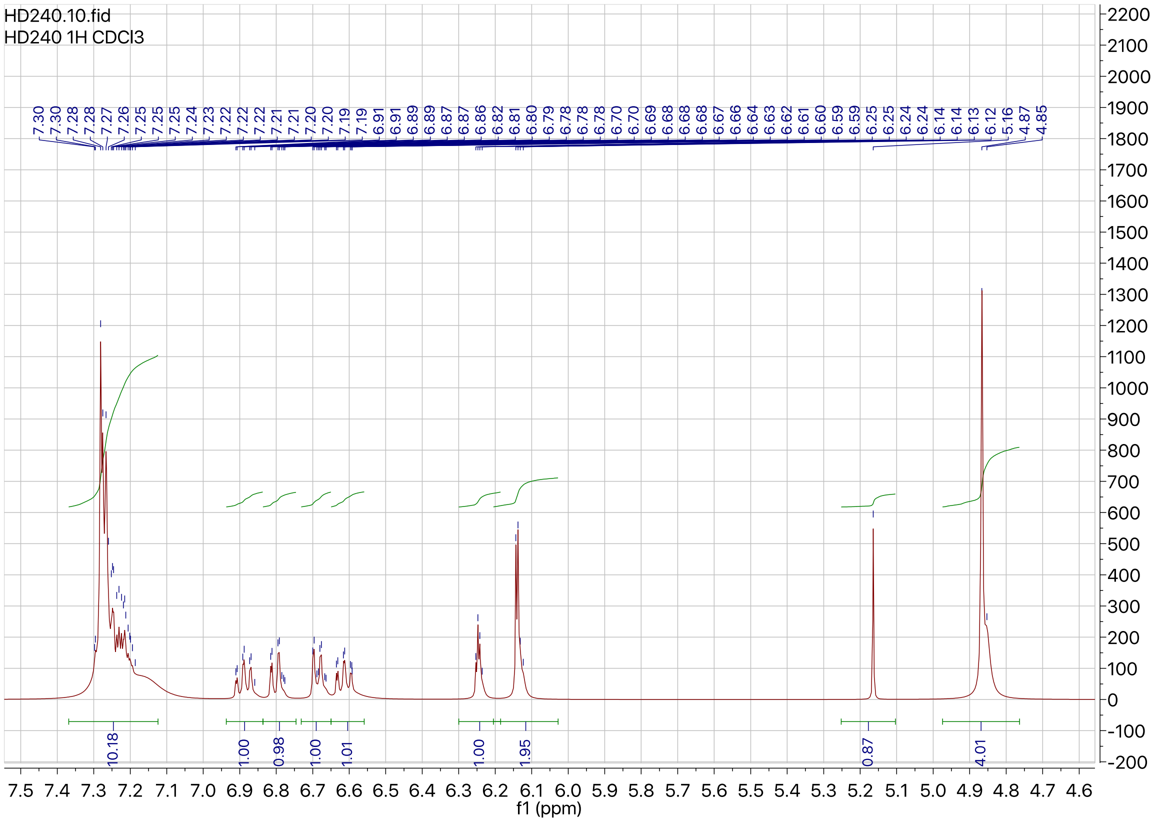

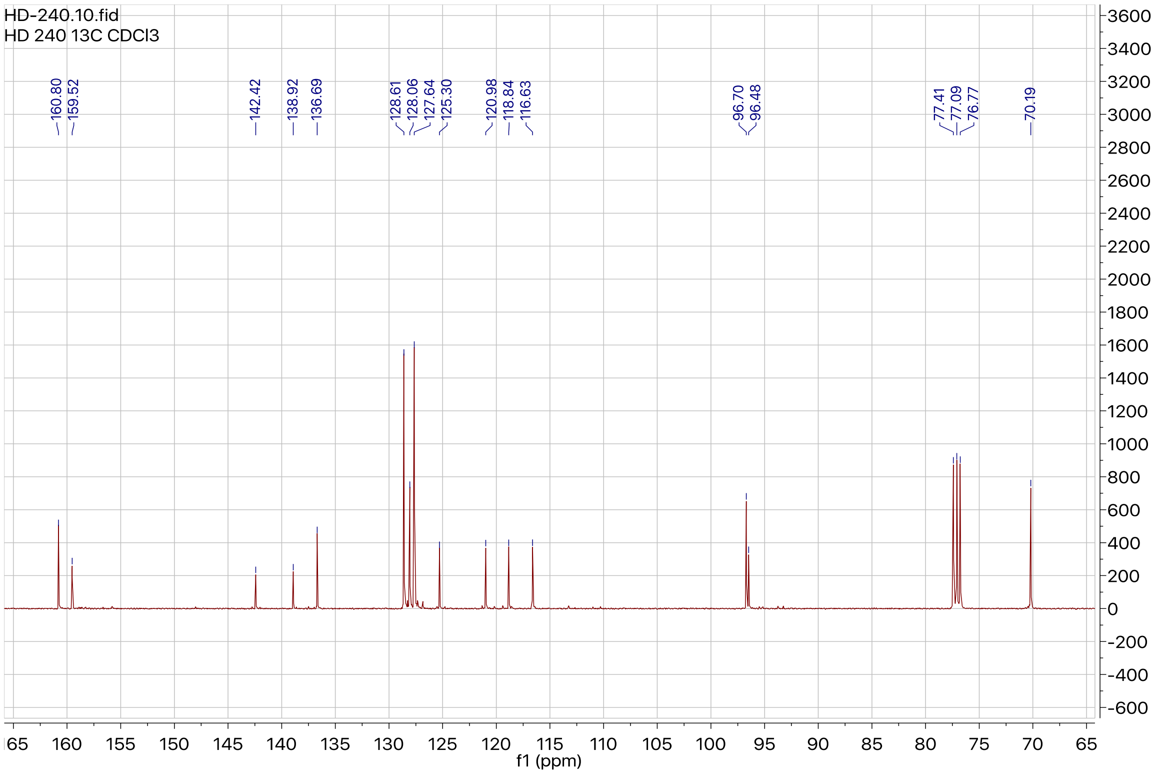

2-(3′,5′-Dimethoxyphenoxy)aniline (10)

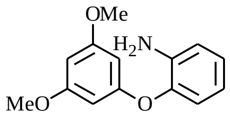

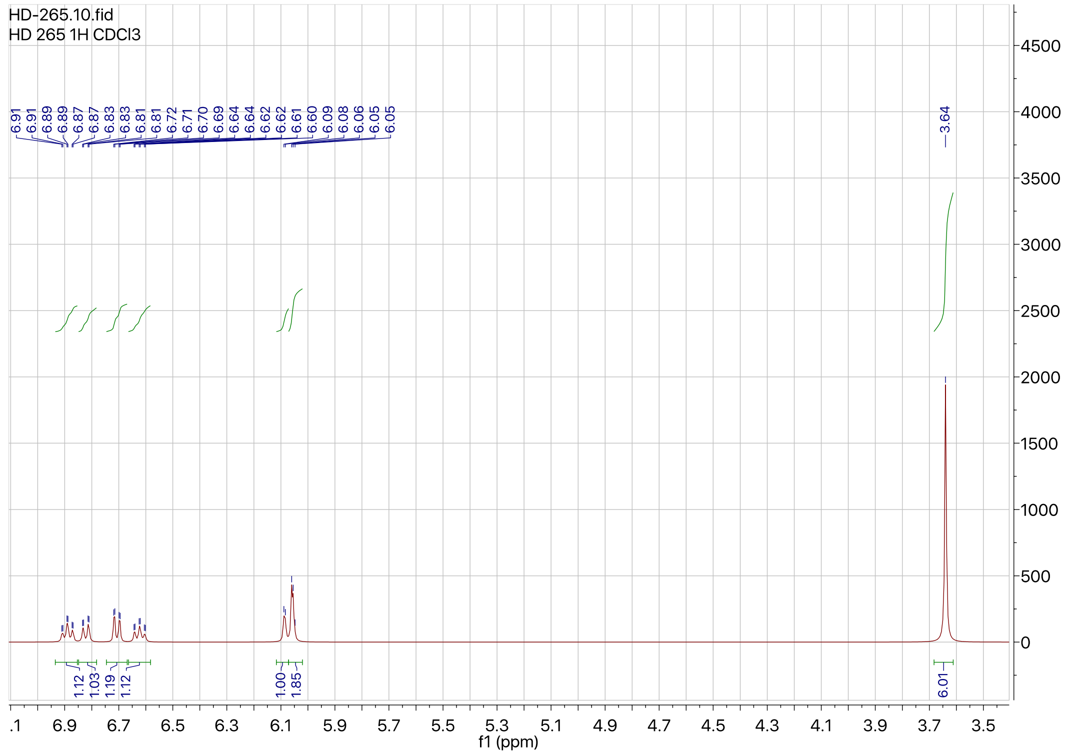

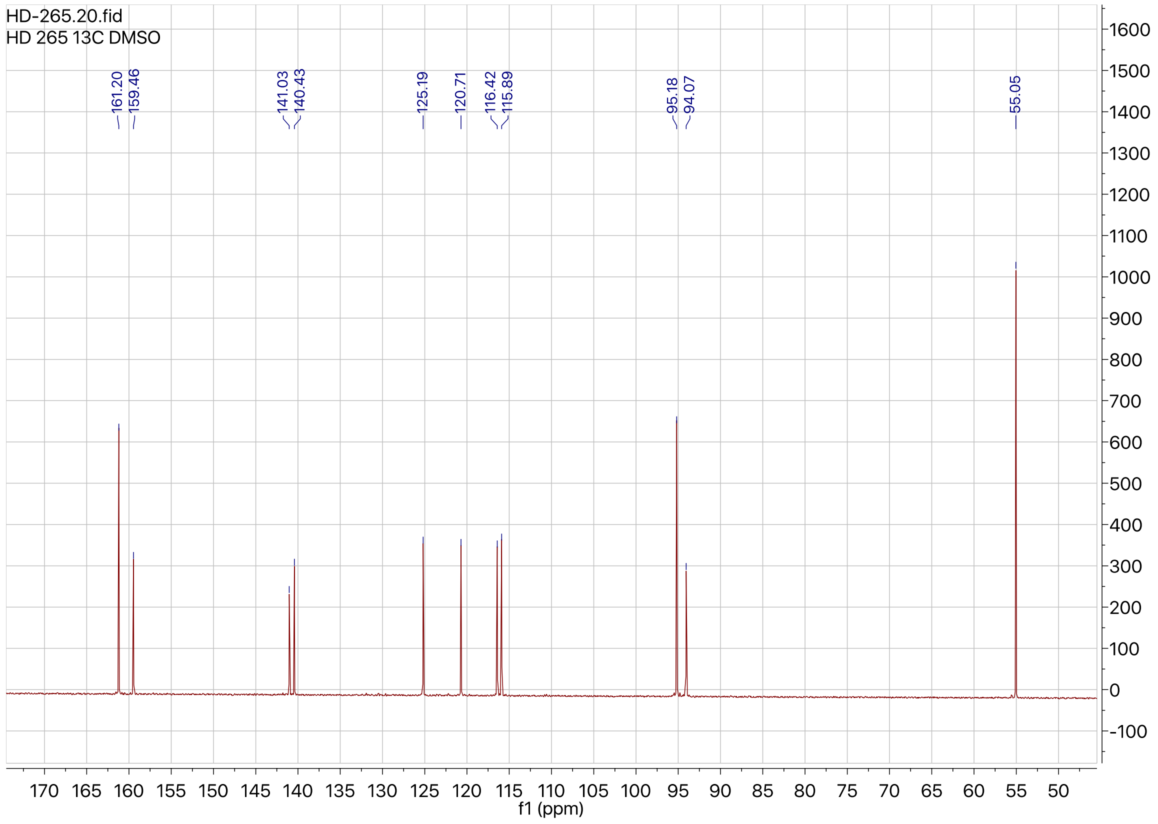

1,3-Dimethoxy-6-nitrodibenzo[*b*,*d*]furan (13)

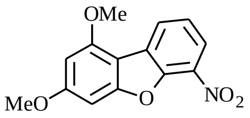

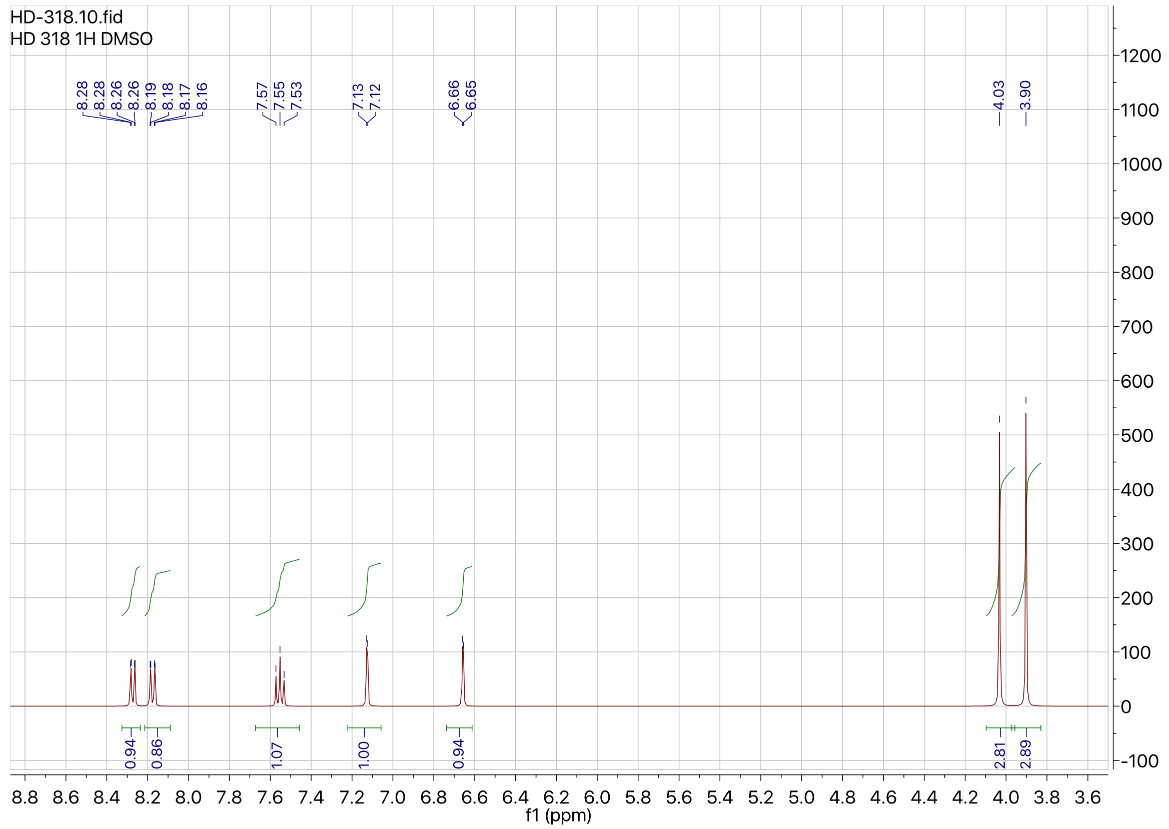

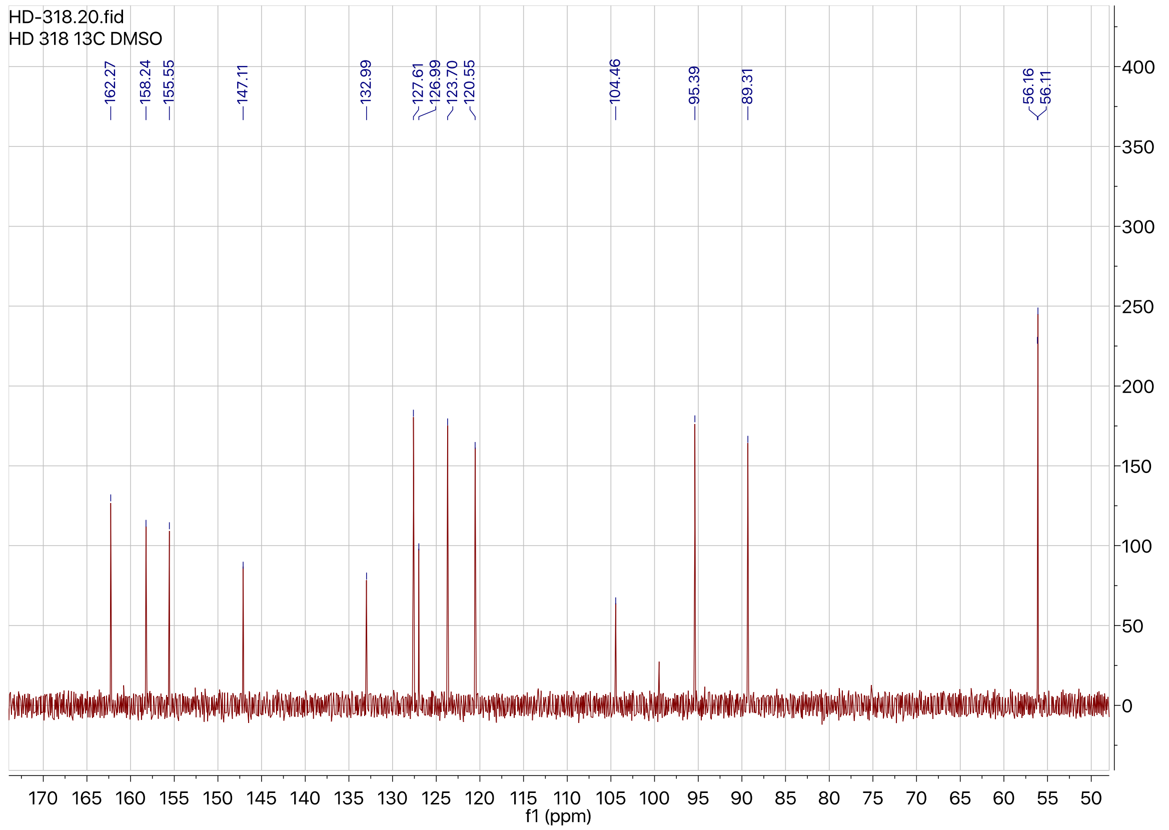

1,3-Dimethoxy-6-nitrodibenzo[*b*,*d*]furan-4-carboxamide (14)

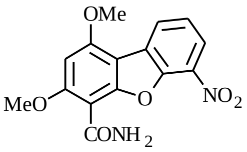

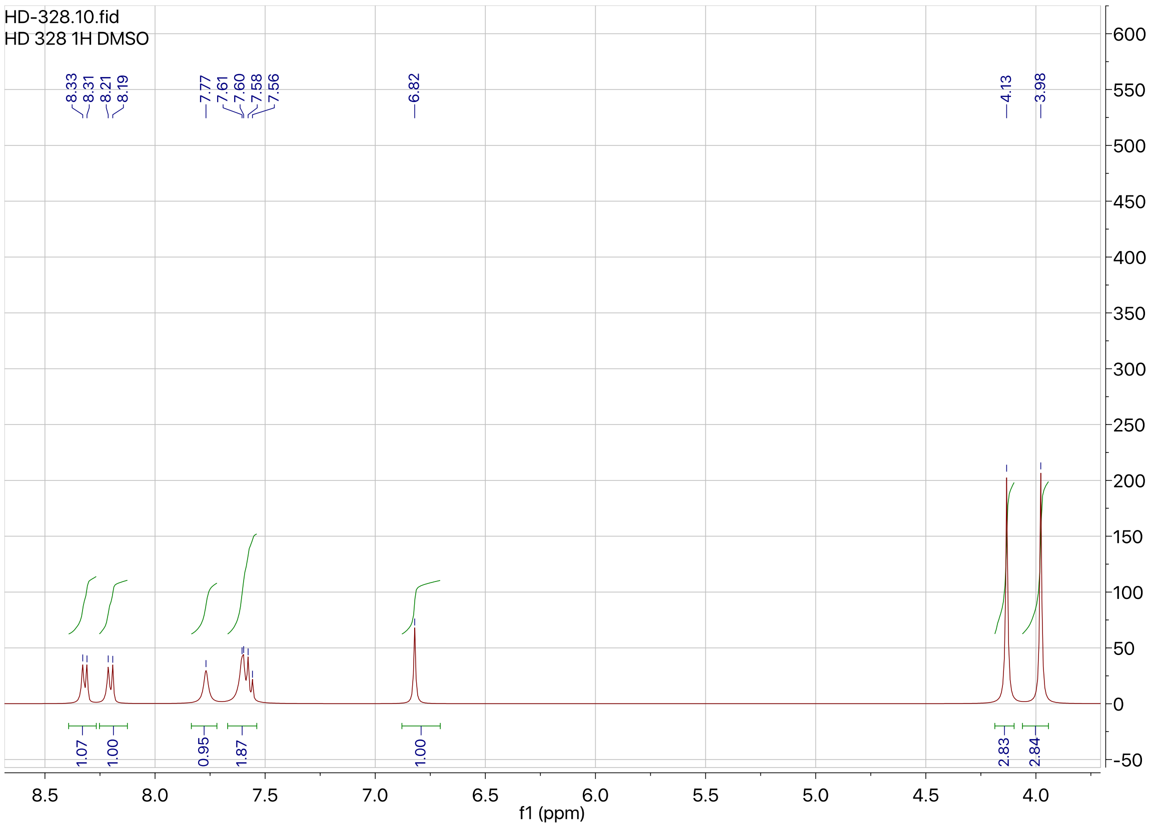

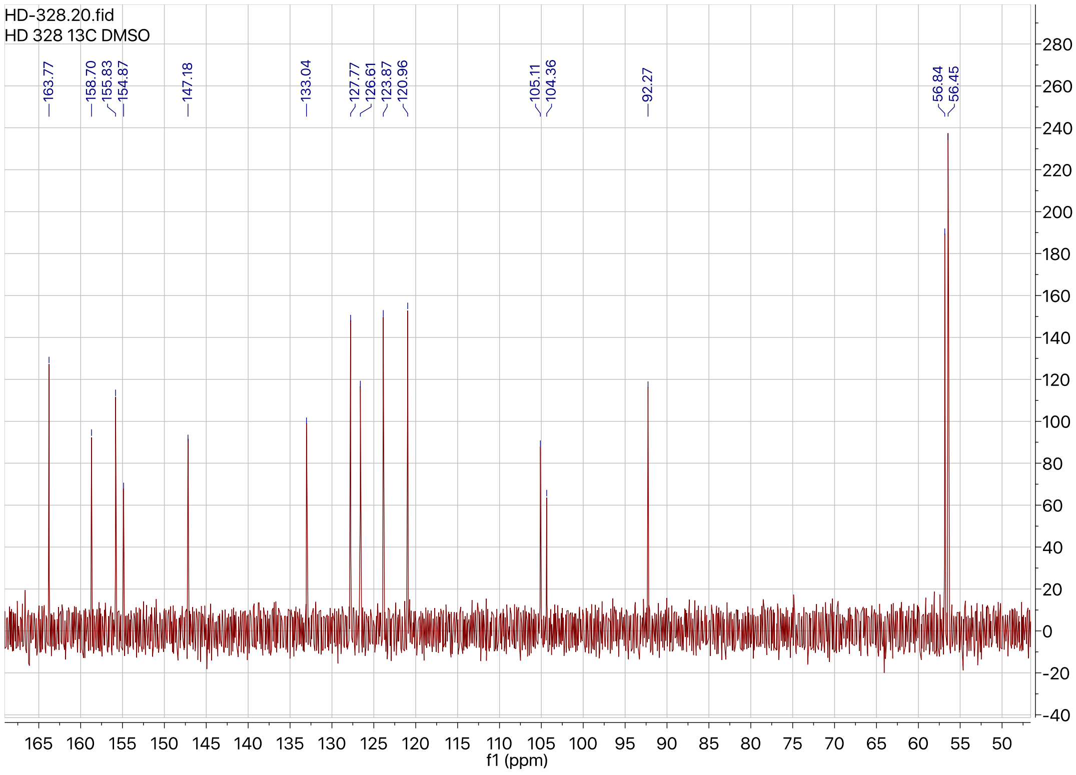

1,3-Dihydroxy-6-nitrodibenzo[*b*,*d*]furan-4-carboxamide (15)

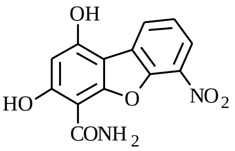

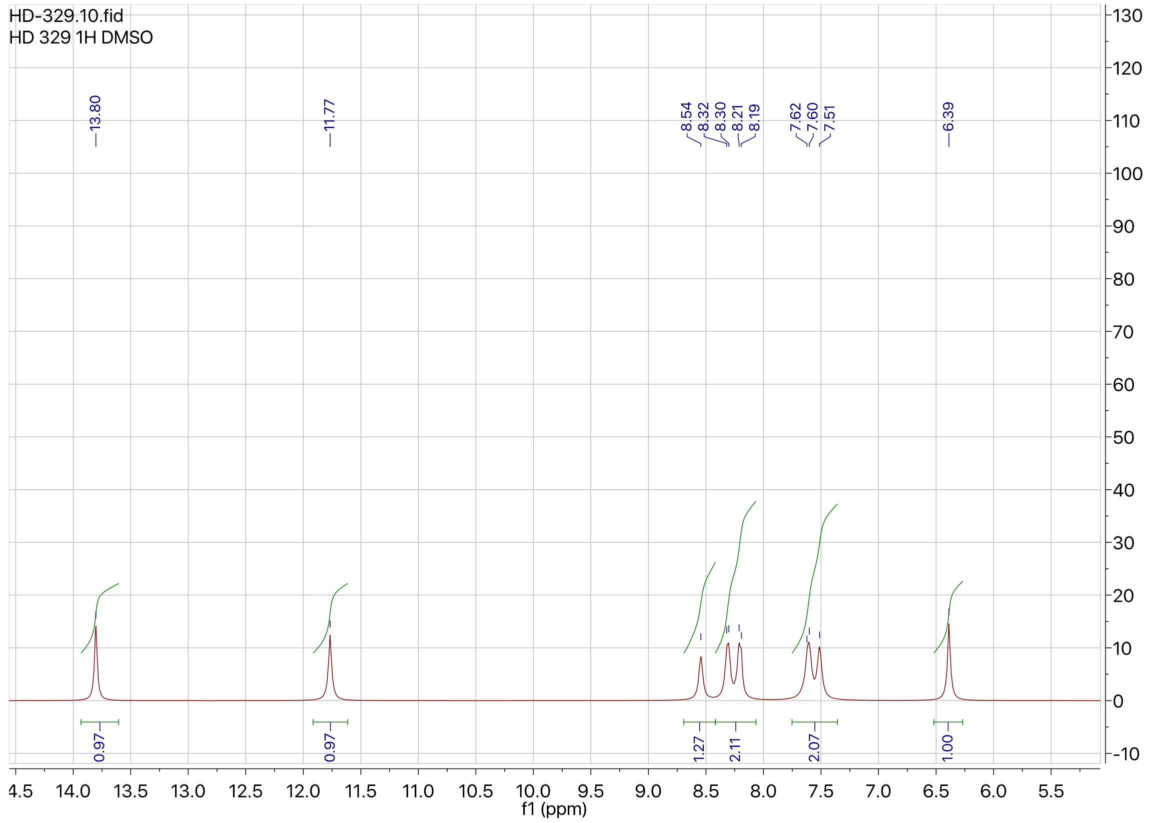

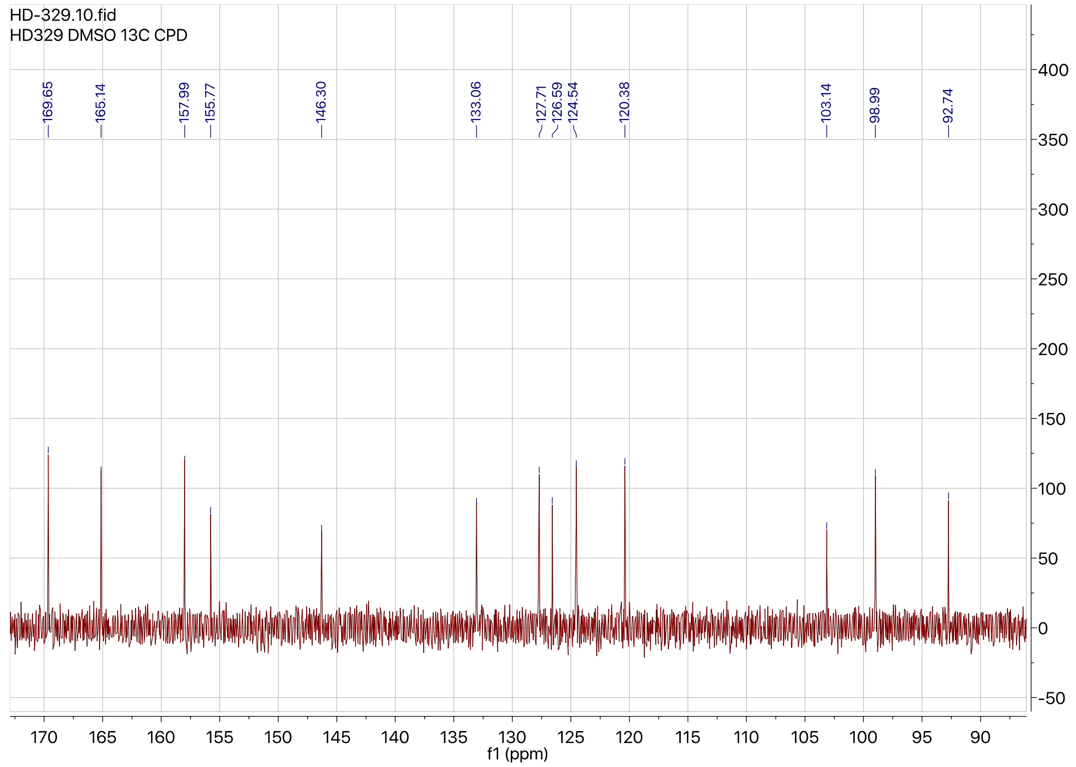

6-Amino-1,3-dihydroxydibenzo[*b,d*]furan-4-carboxamide (16)

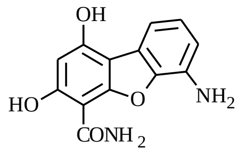

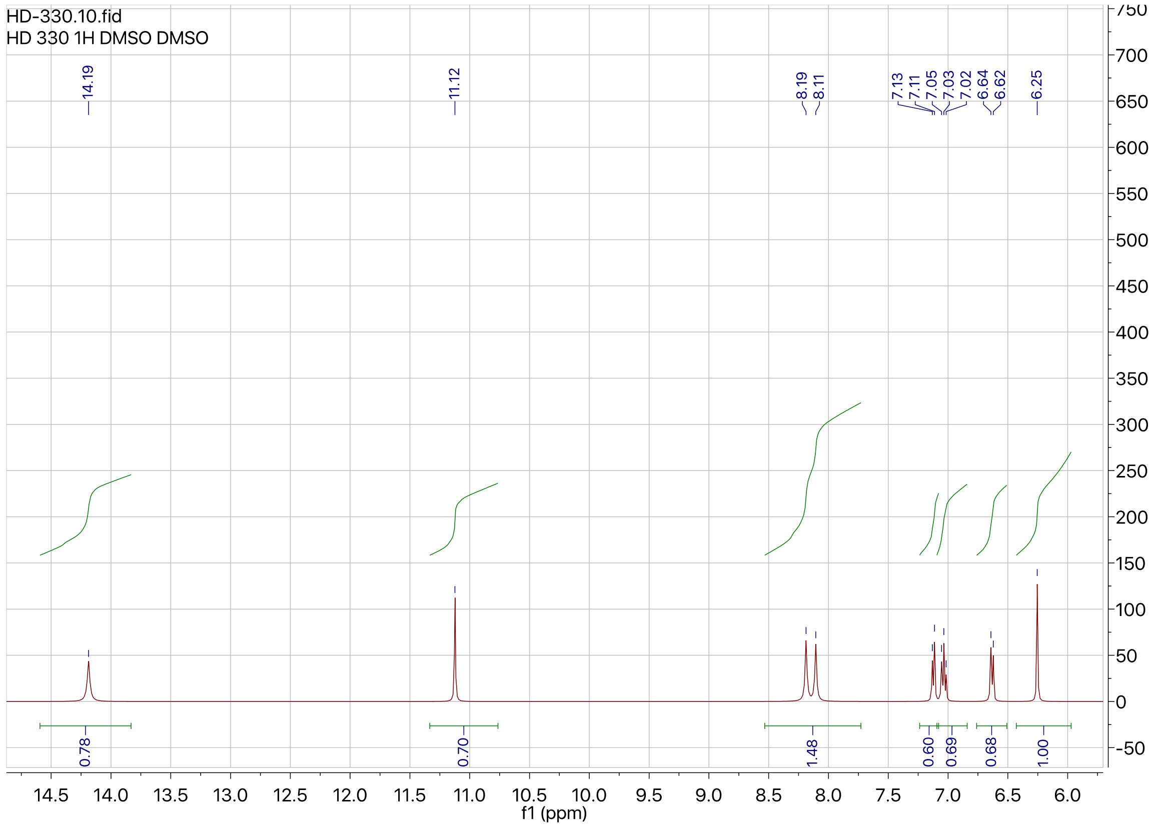

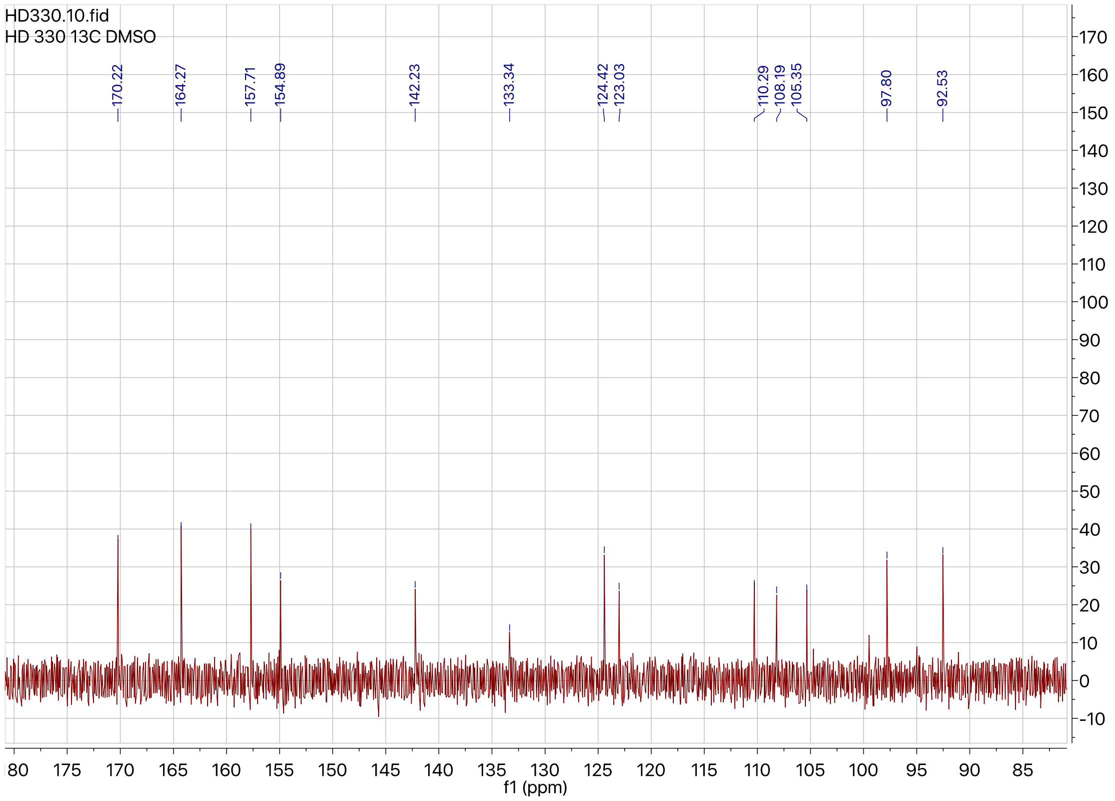

1,3-Dihydroxydibenzo[*b*,*d*]furan-4-carboxamide (17)

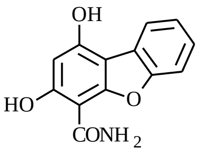

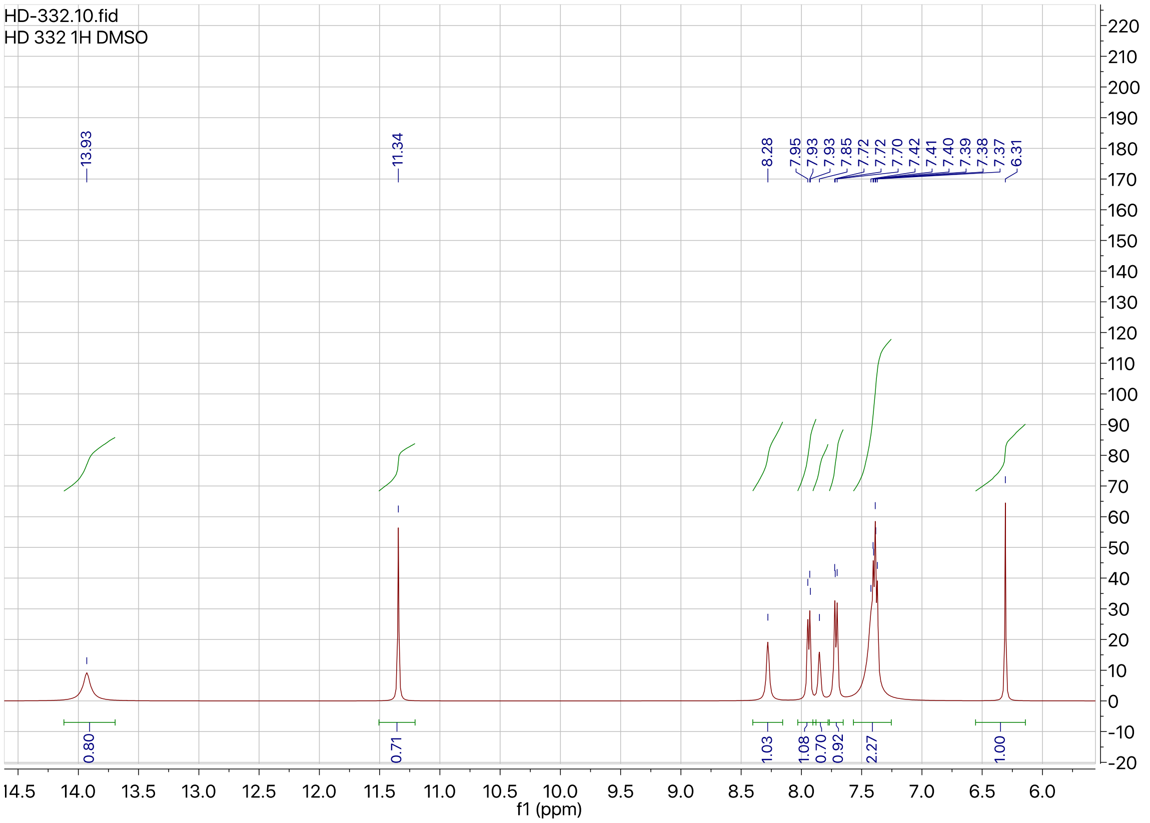

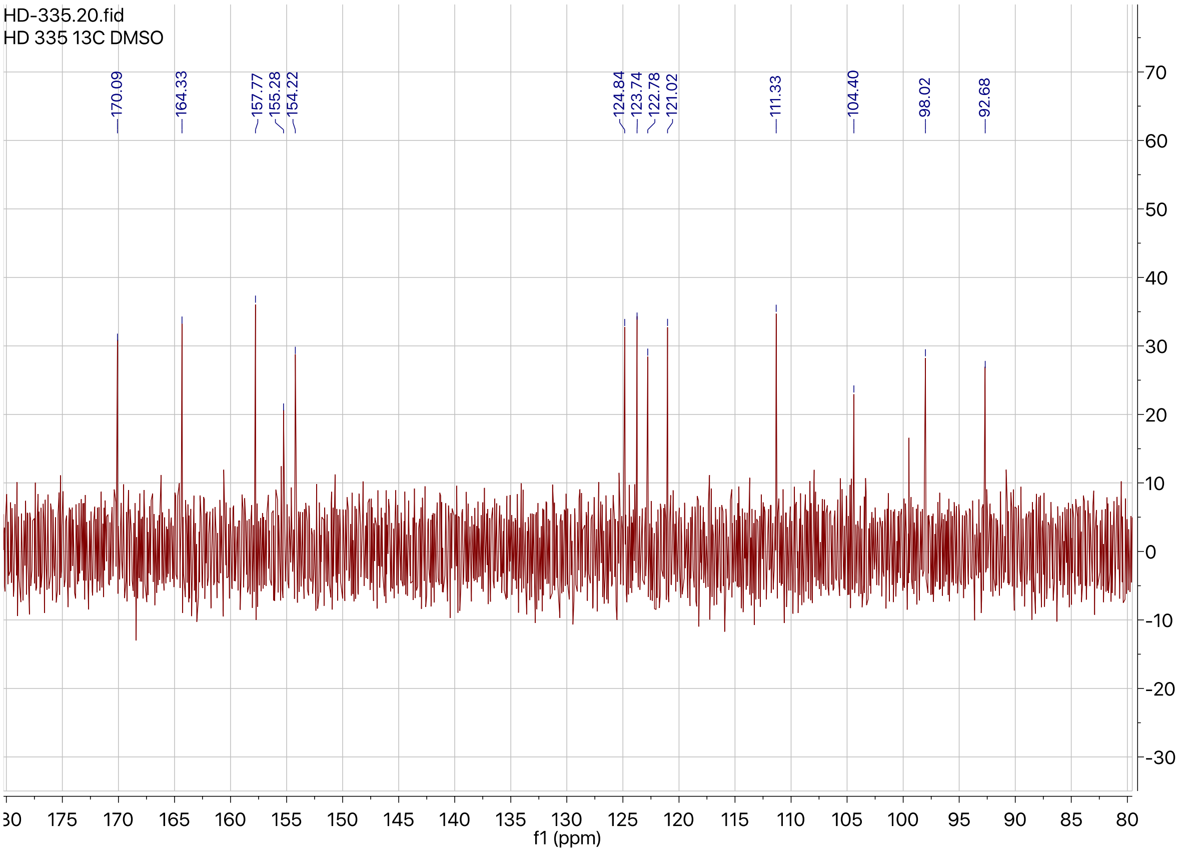

6-Amino-1,3-dimethoxydibenzo[*b*,*d*]furan-4-carboxamide (18)

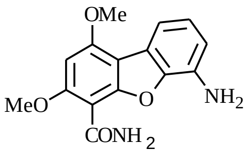

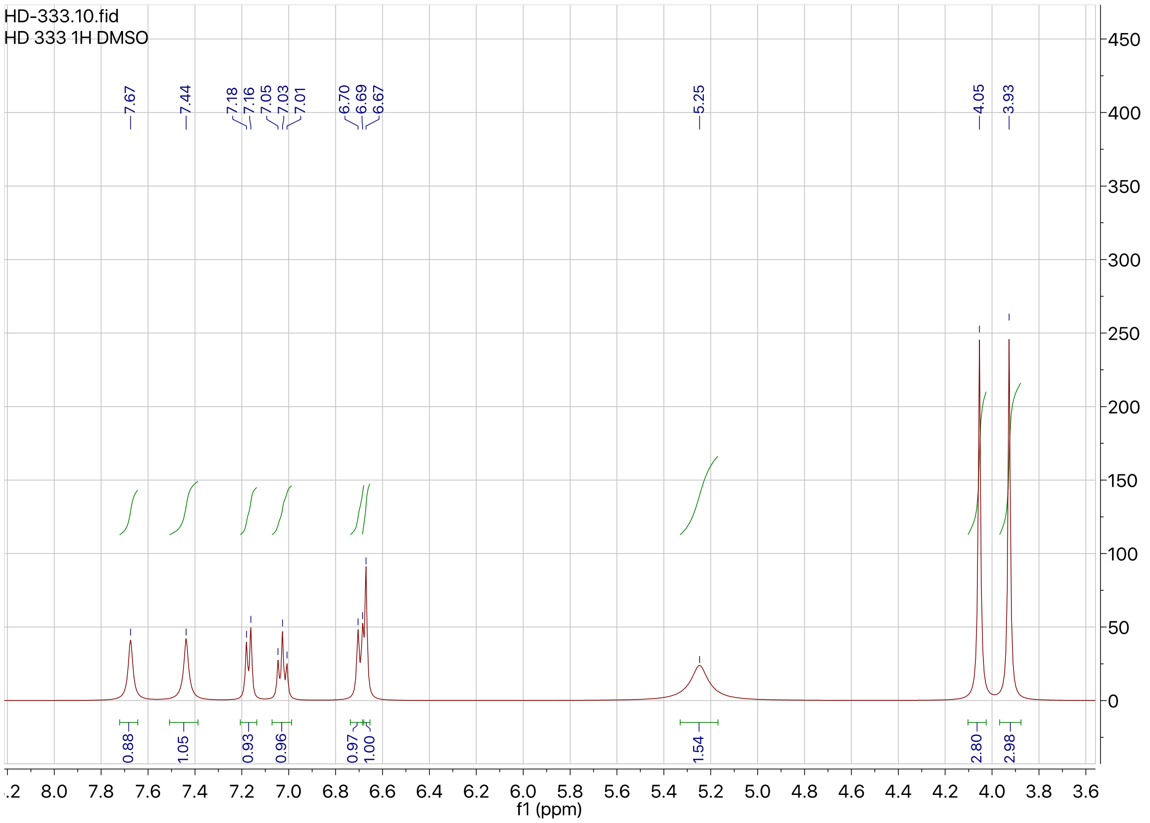

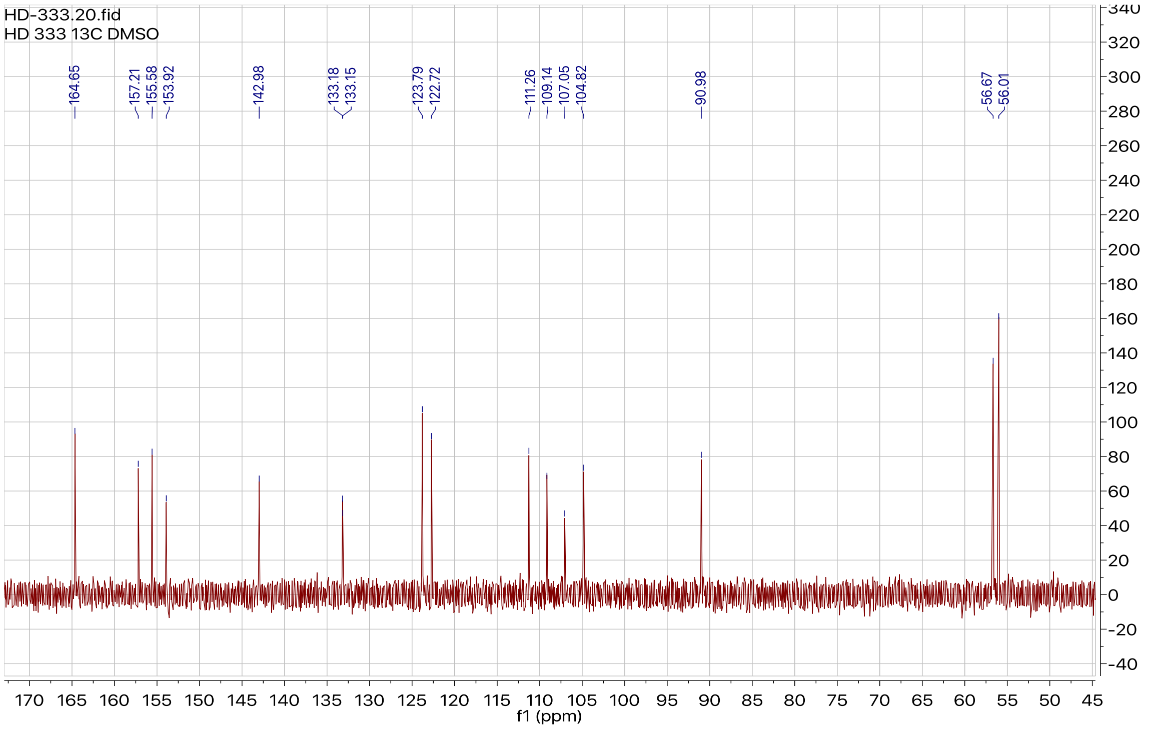

1,3-Dimethoxydibenzo[*b,d*]furan-4-carboxamide (19)

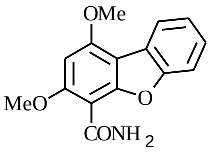

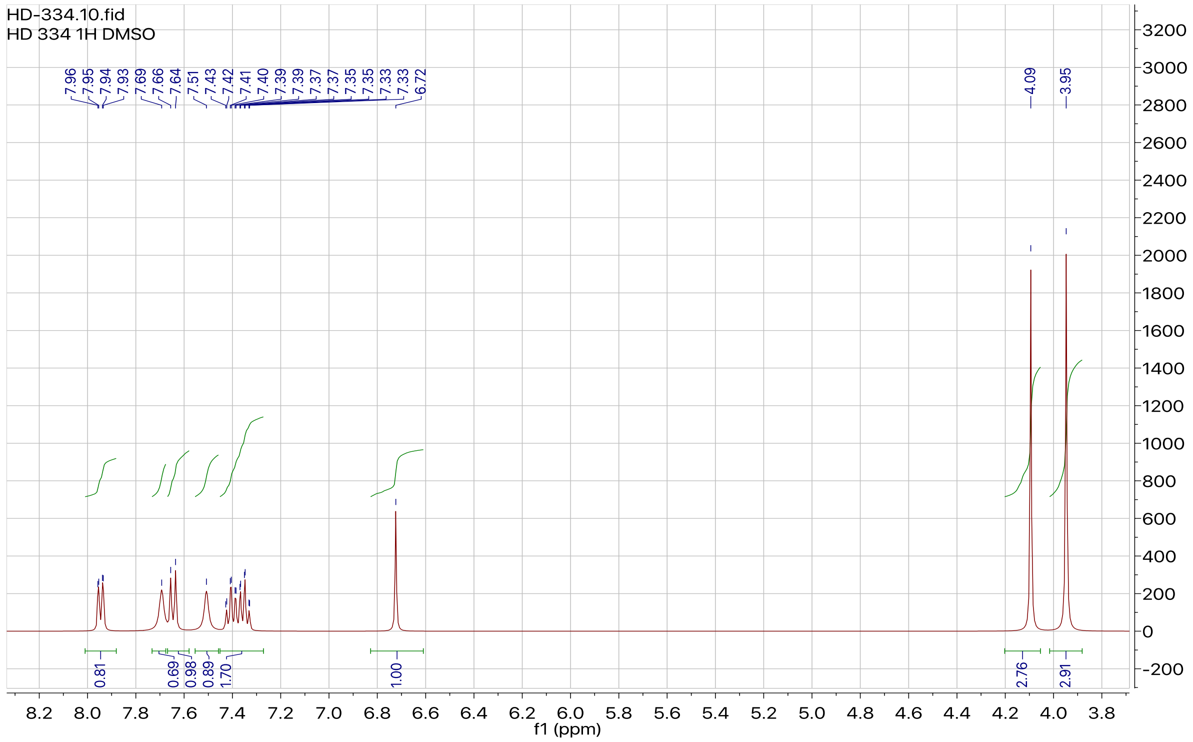

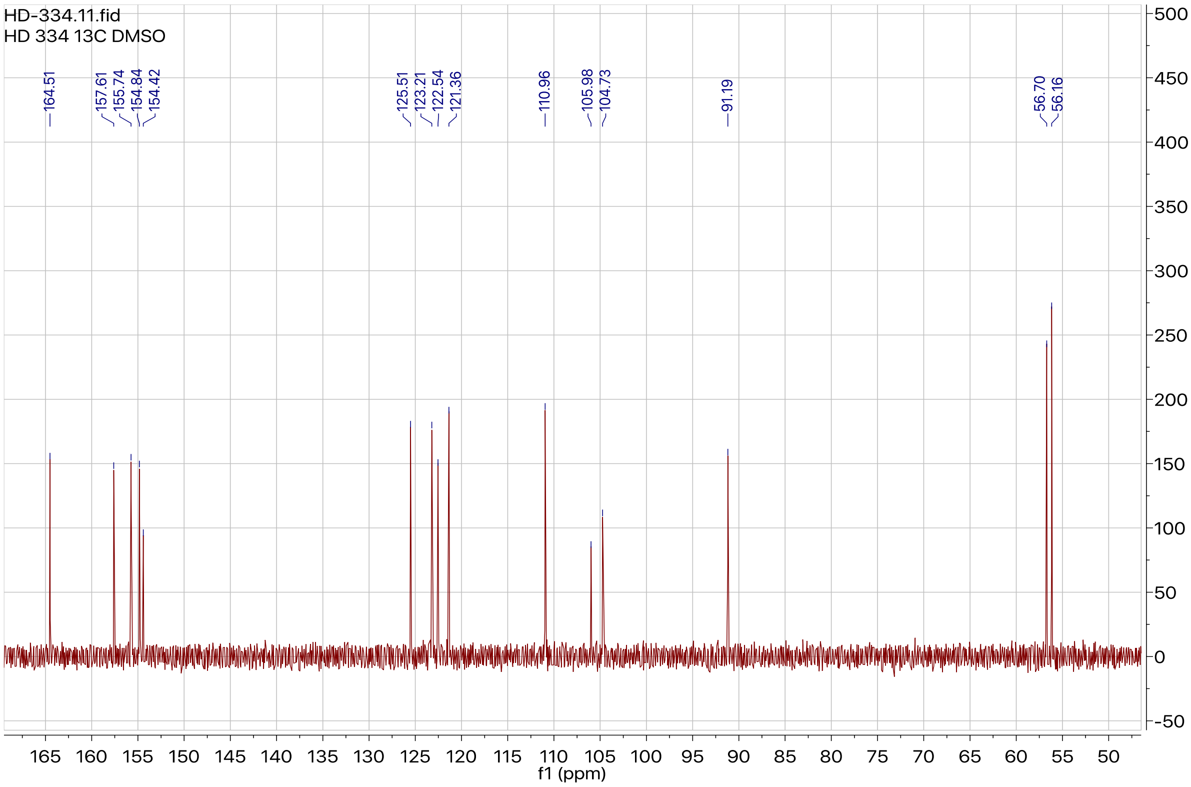

3- Iodophenyl acetate (21)

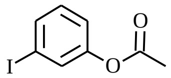

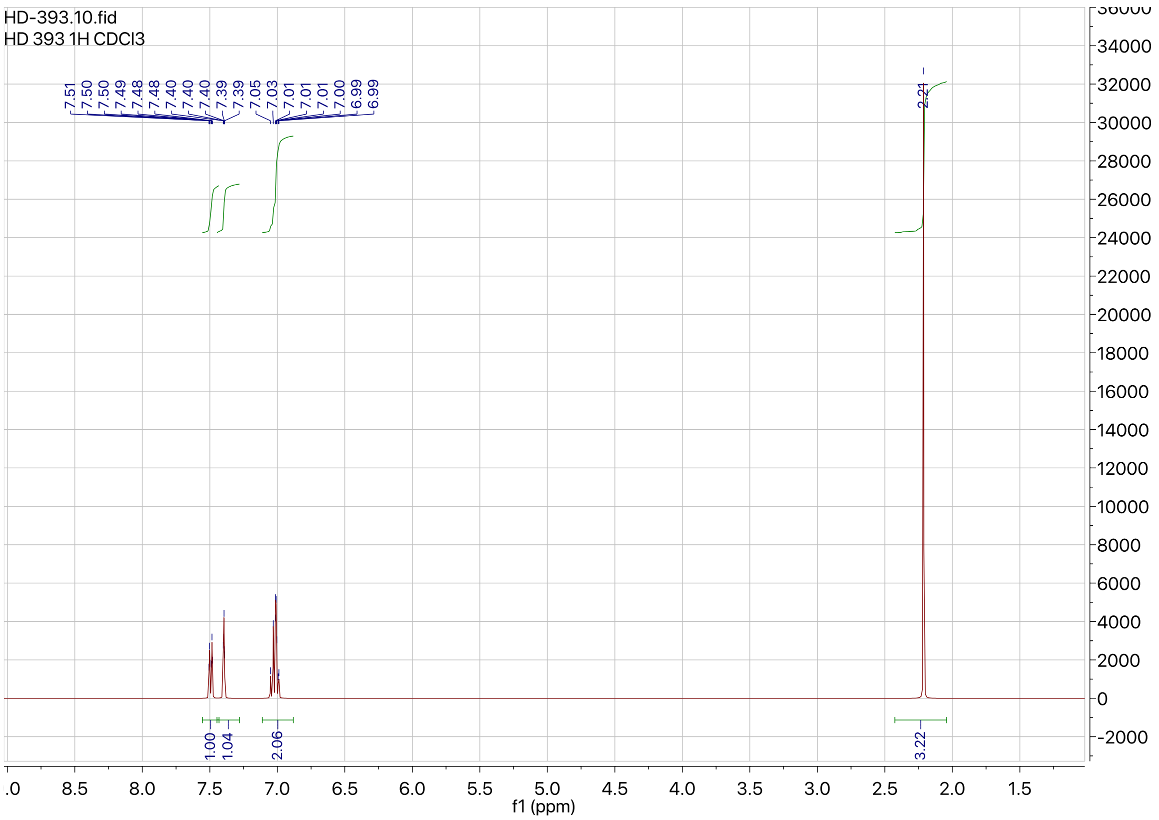

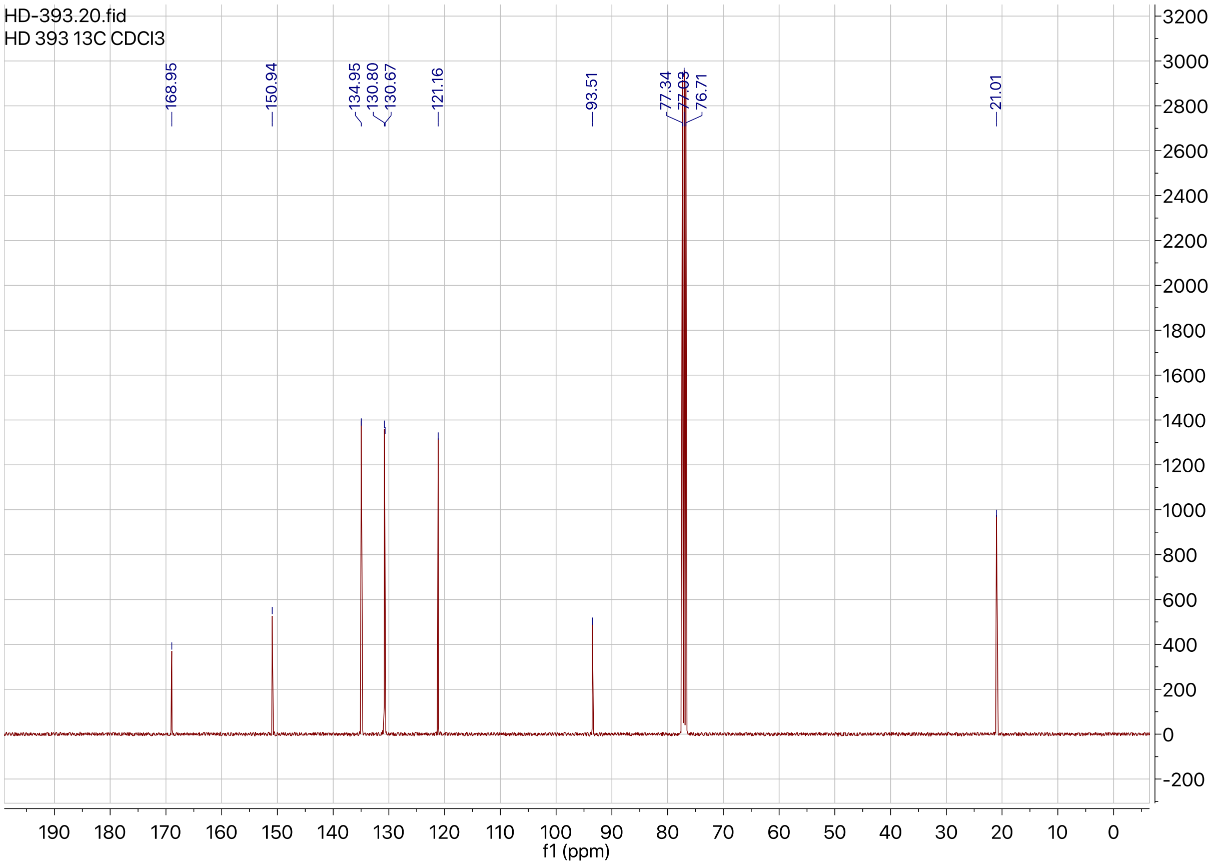

1-(2-Hydroxy-4-iodophenyl)ethanone (22)

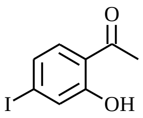

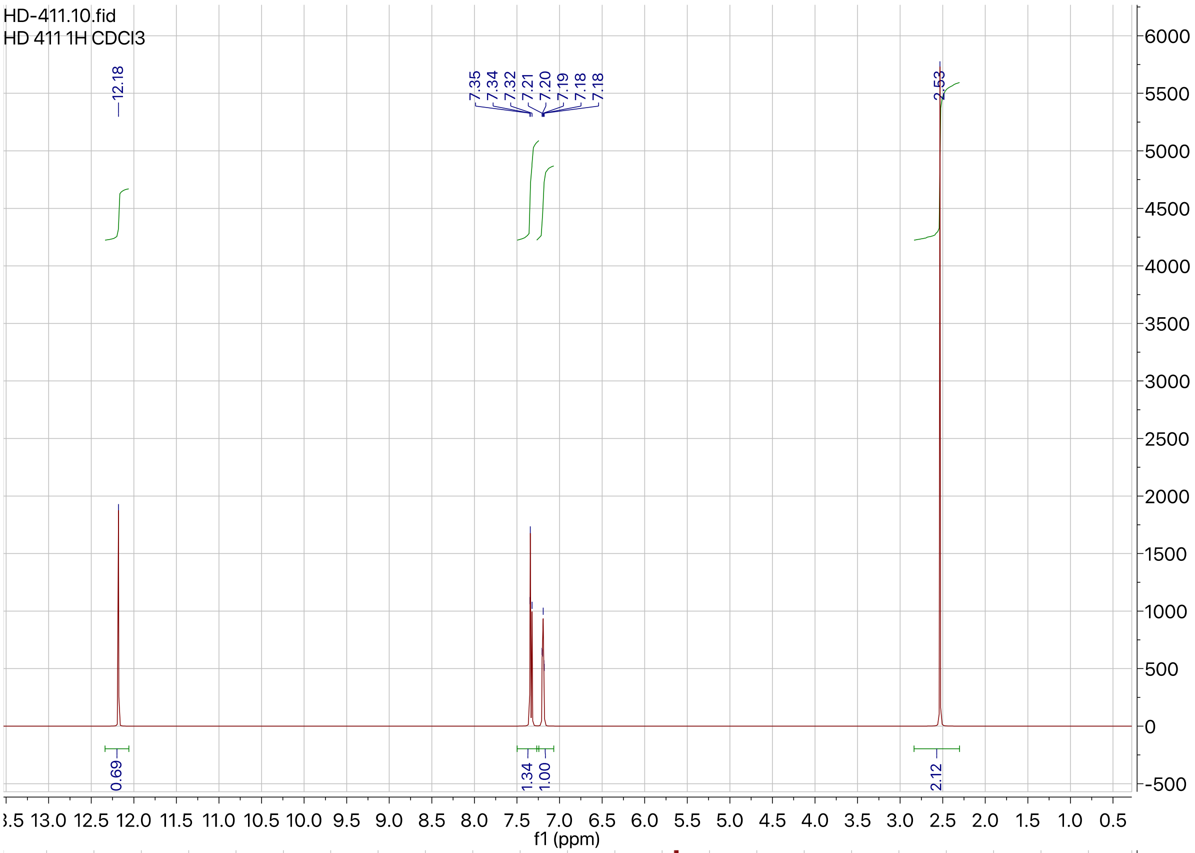

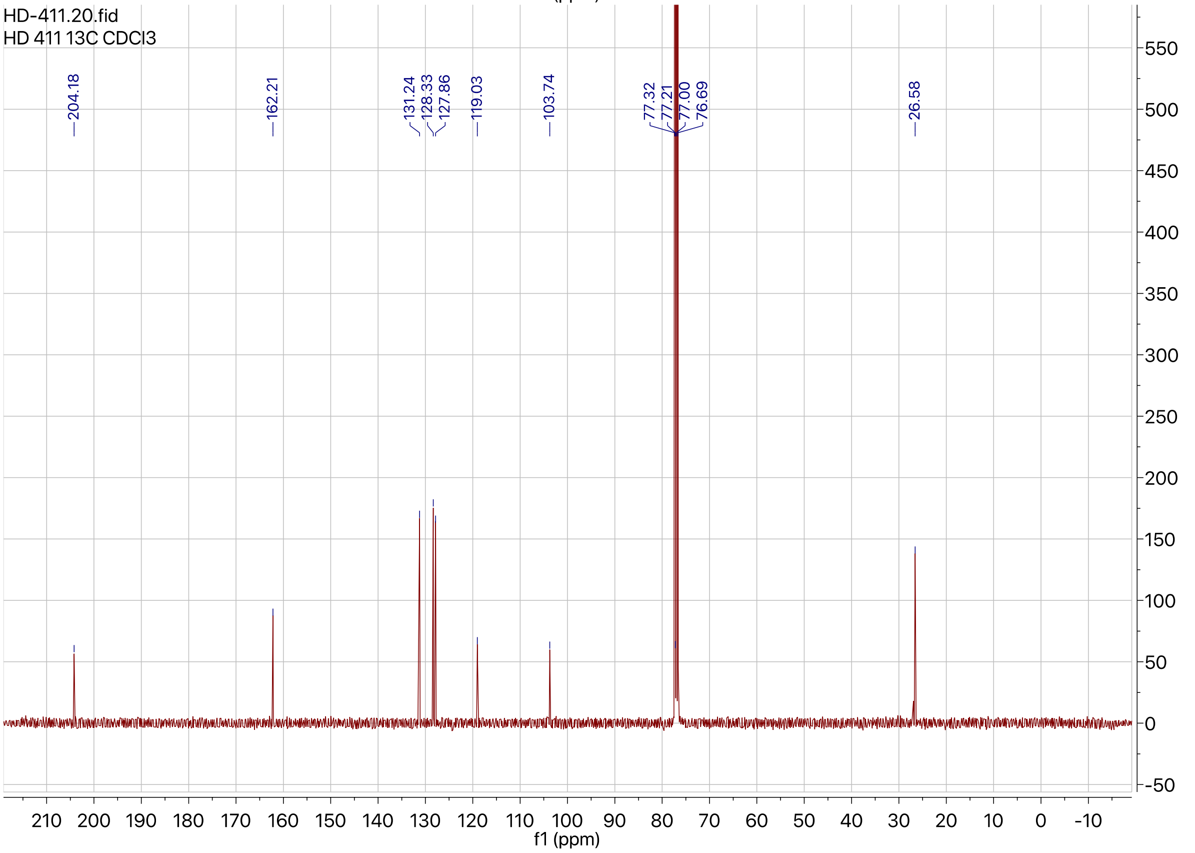

1-(4-Iodo-2-methoxyphenyl)ethanone (23)

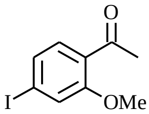

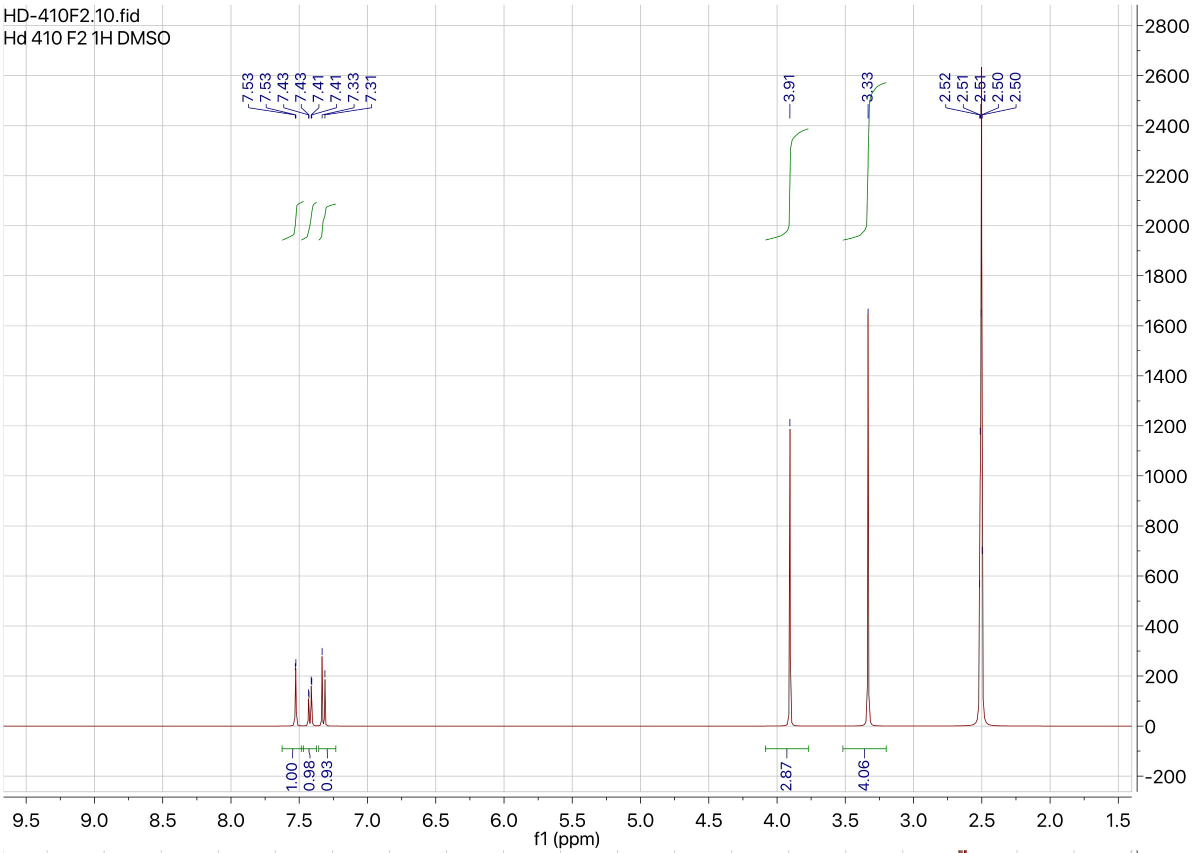

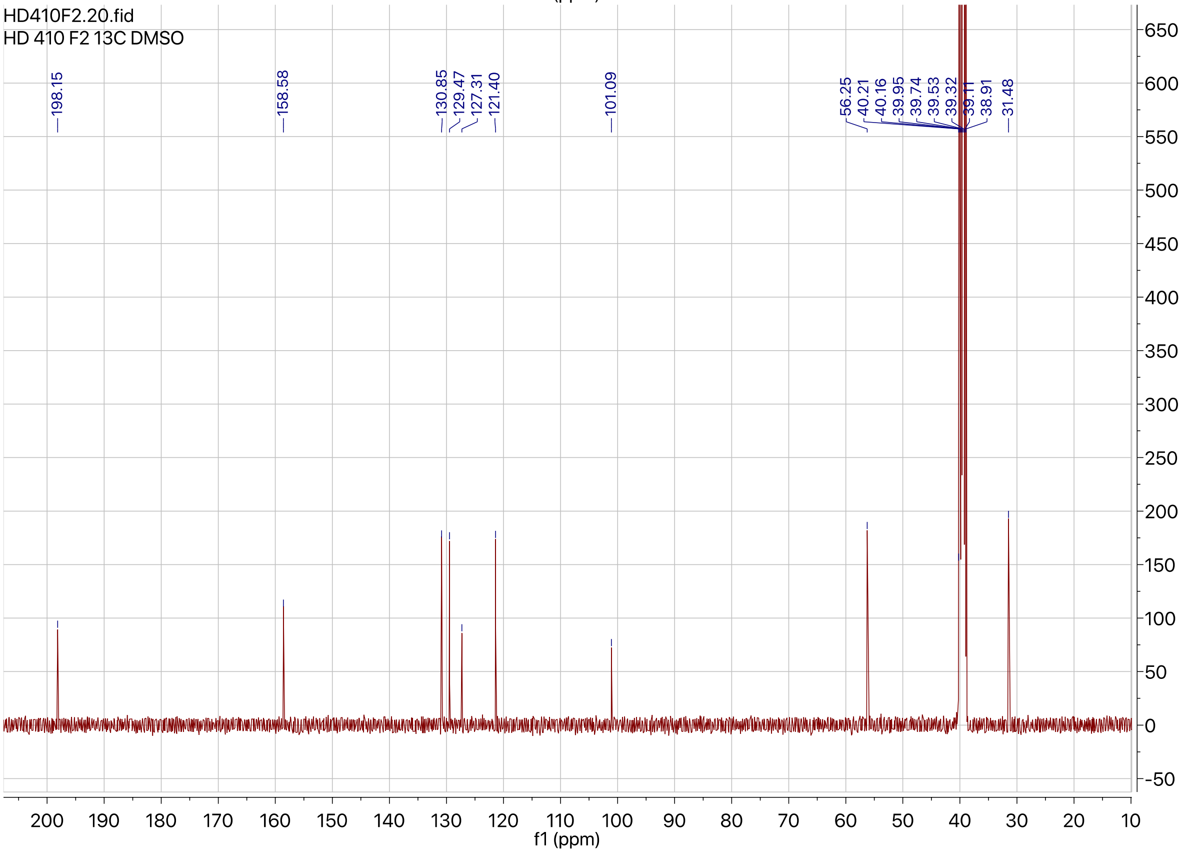

1-Iodo-3-methoxybenzene (24)

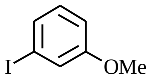

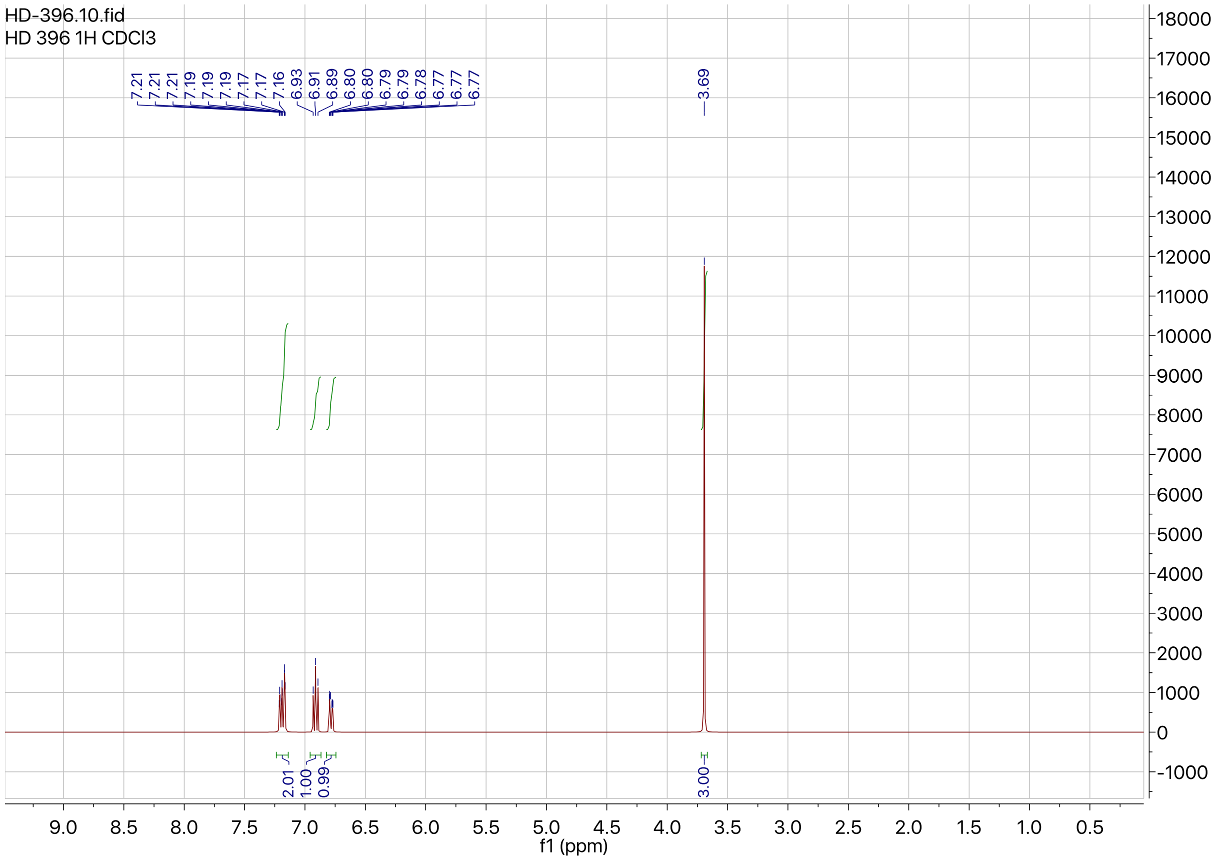

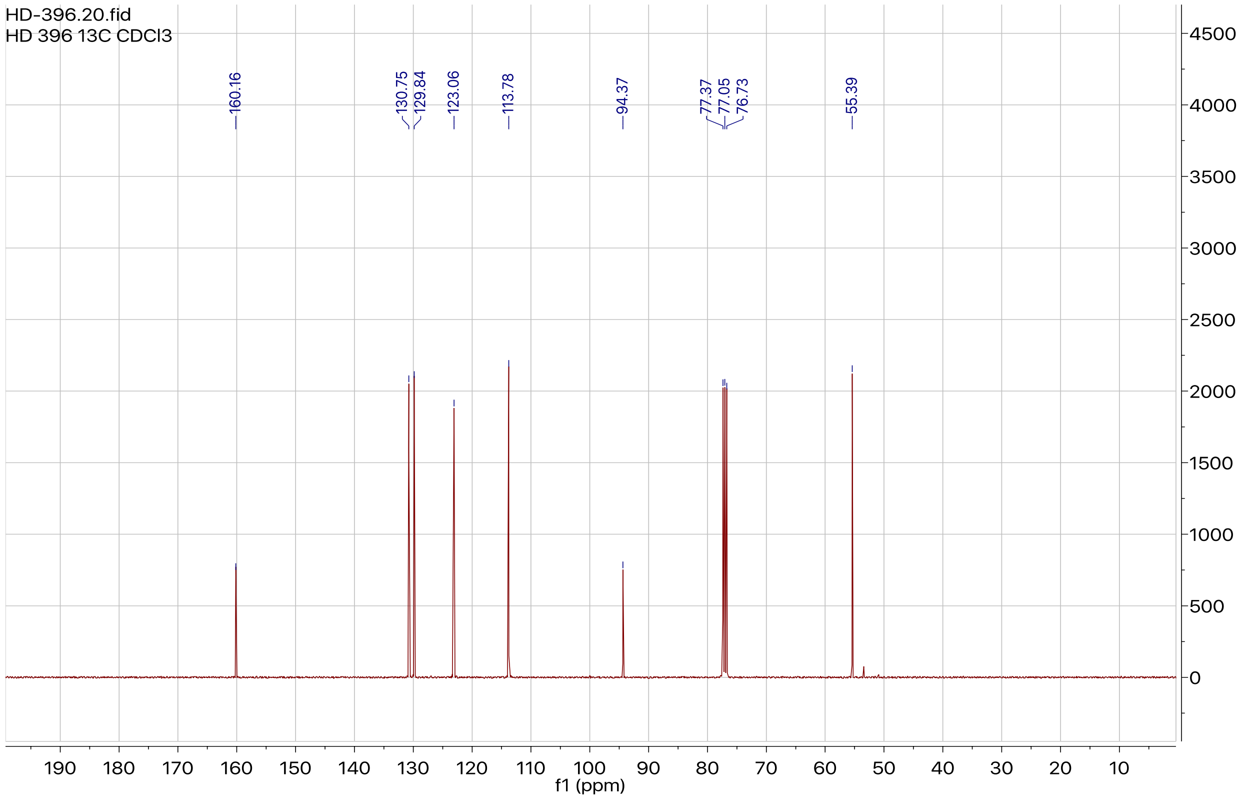

1-[4-(3′,5′-Dimethoxyphenoxy)-2-methoxyphenyl]ethanone (28)

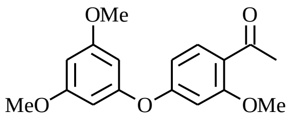

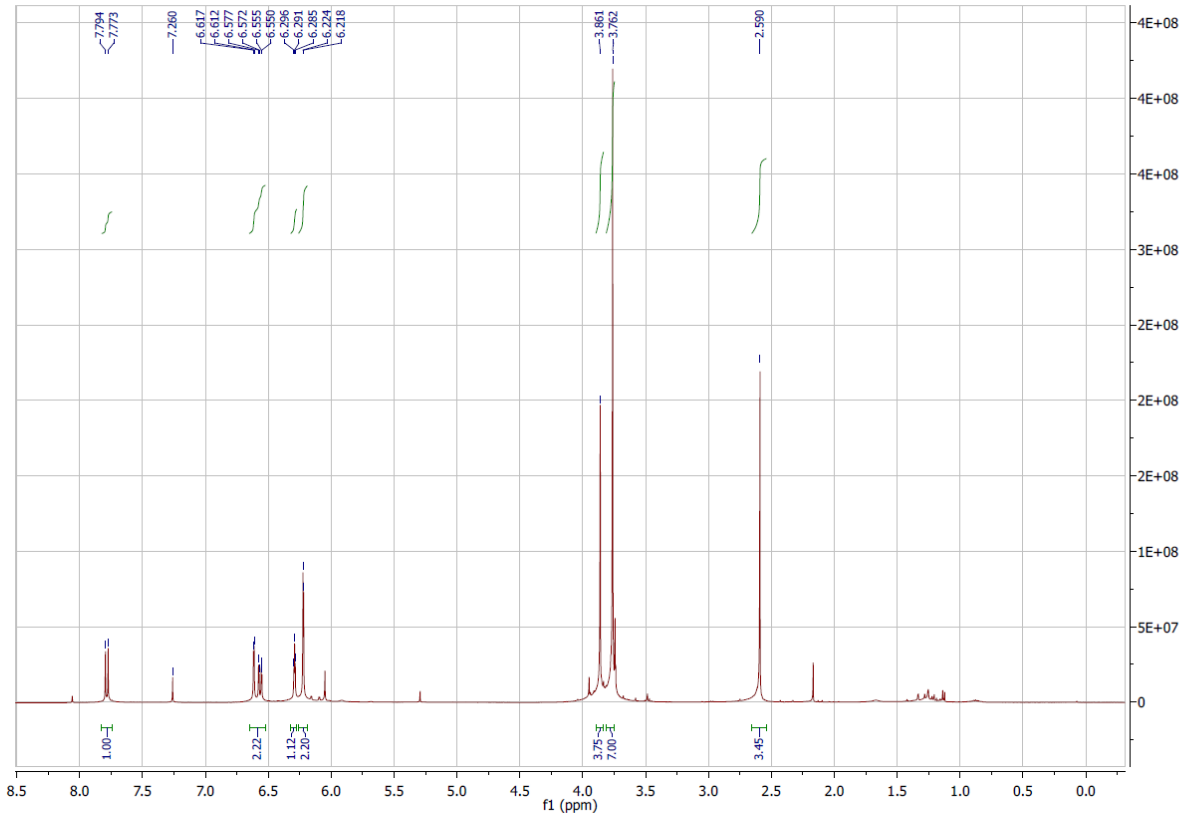

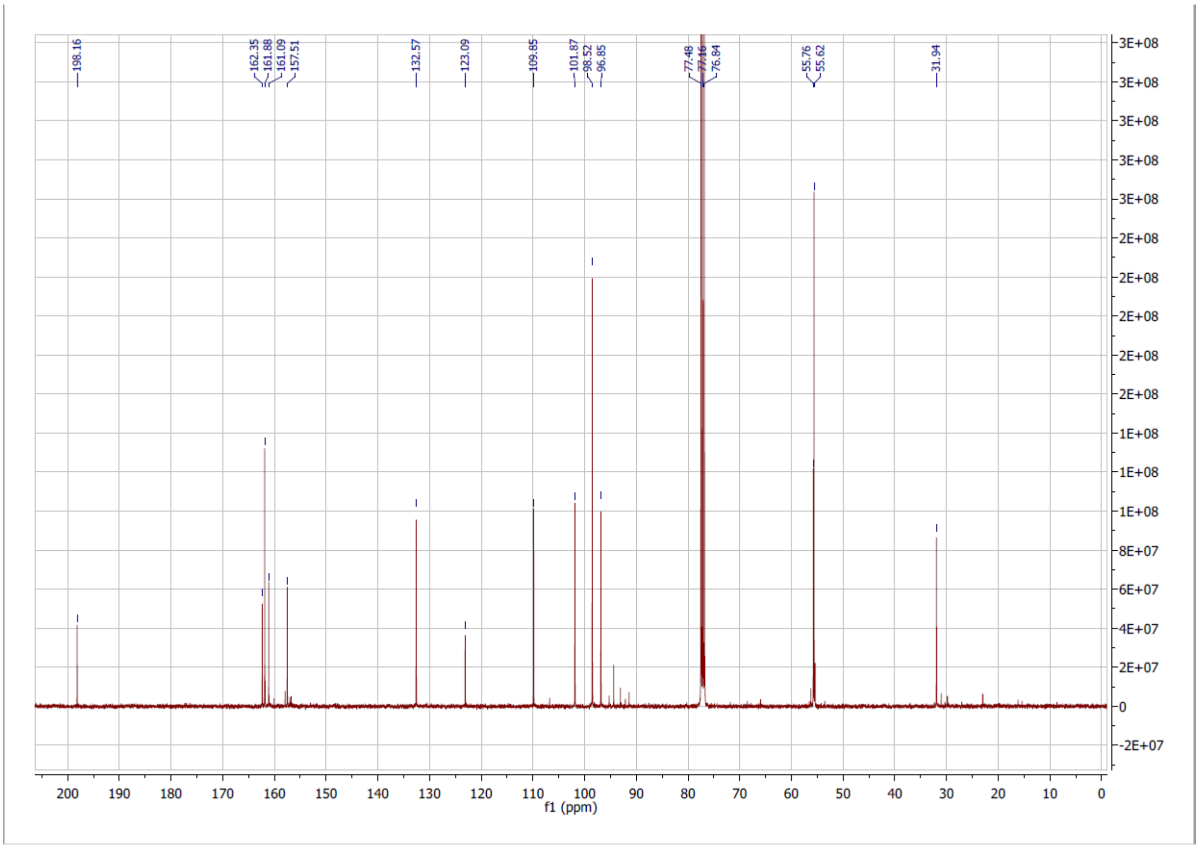

1,3-Dimethoxy-5-(3′-methoxyphenoxy)benzene (29)

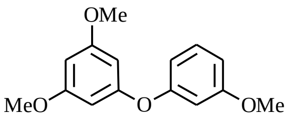

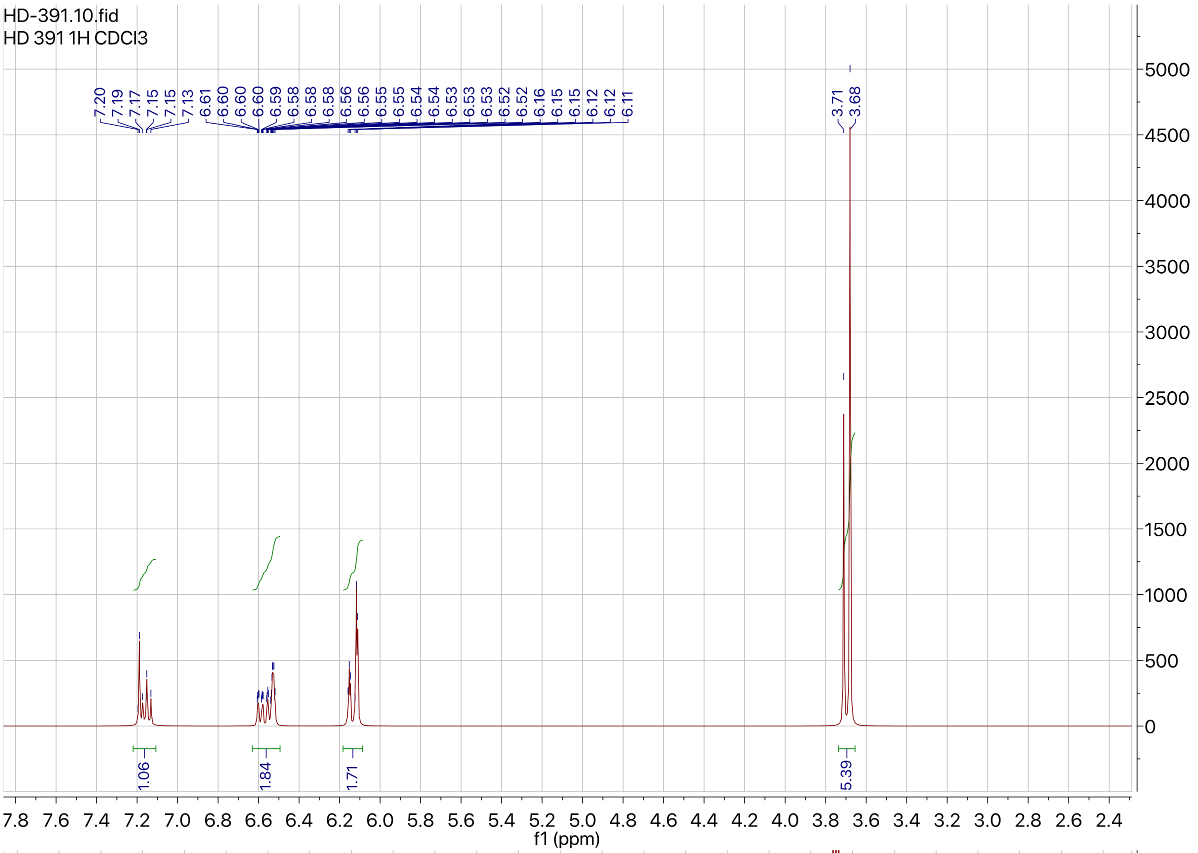

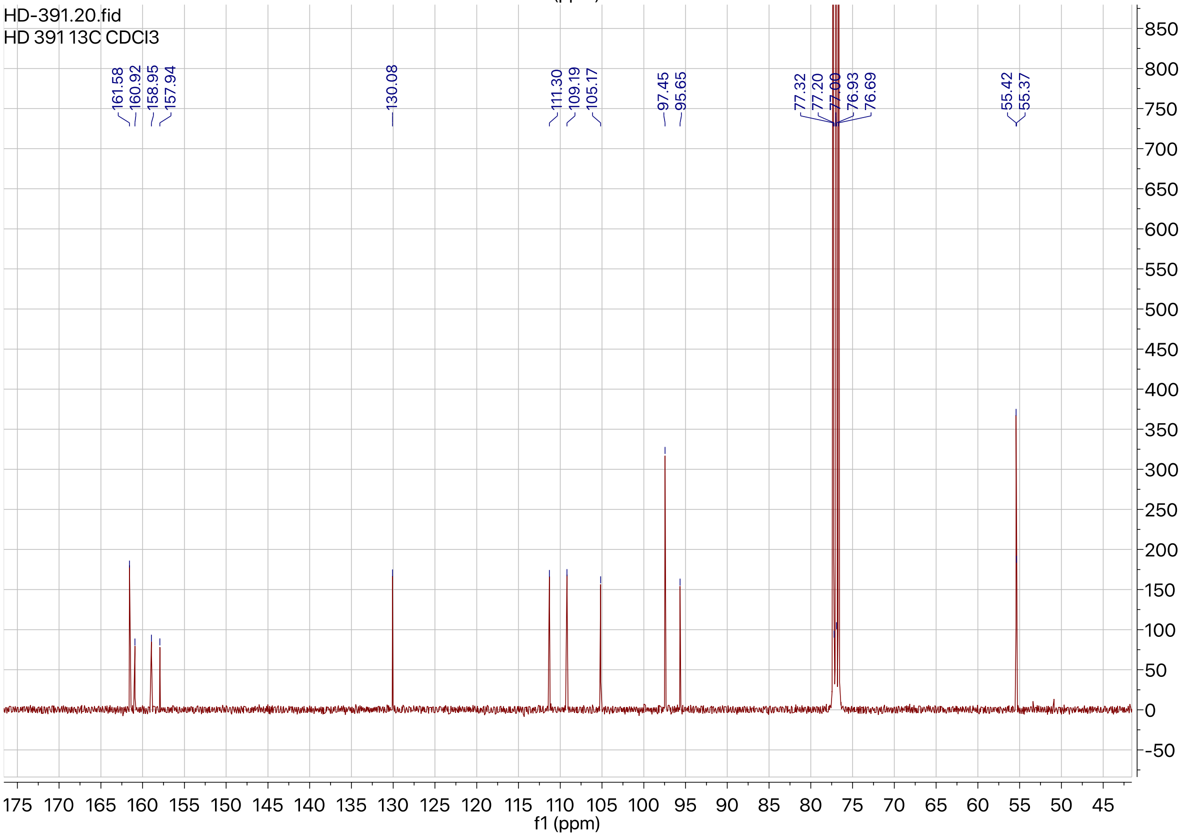

1-[4-(3′,5′-Dimethoxyphenoxy)phenyl]ethanone (30)

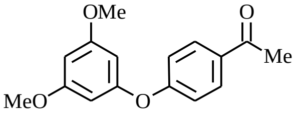

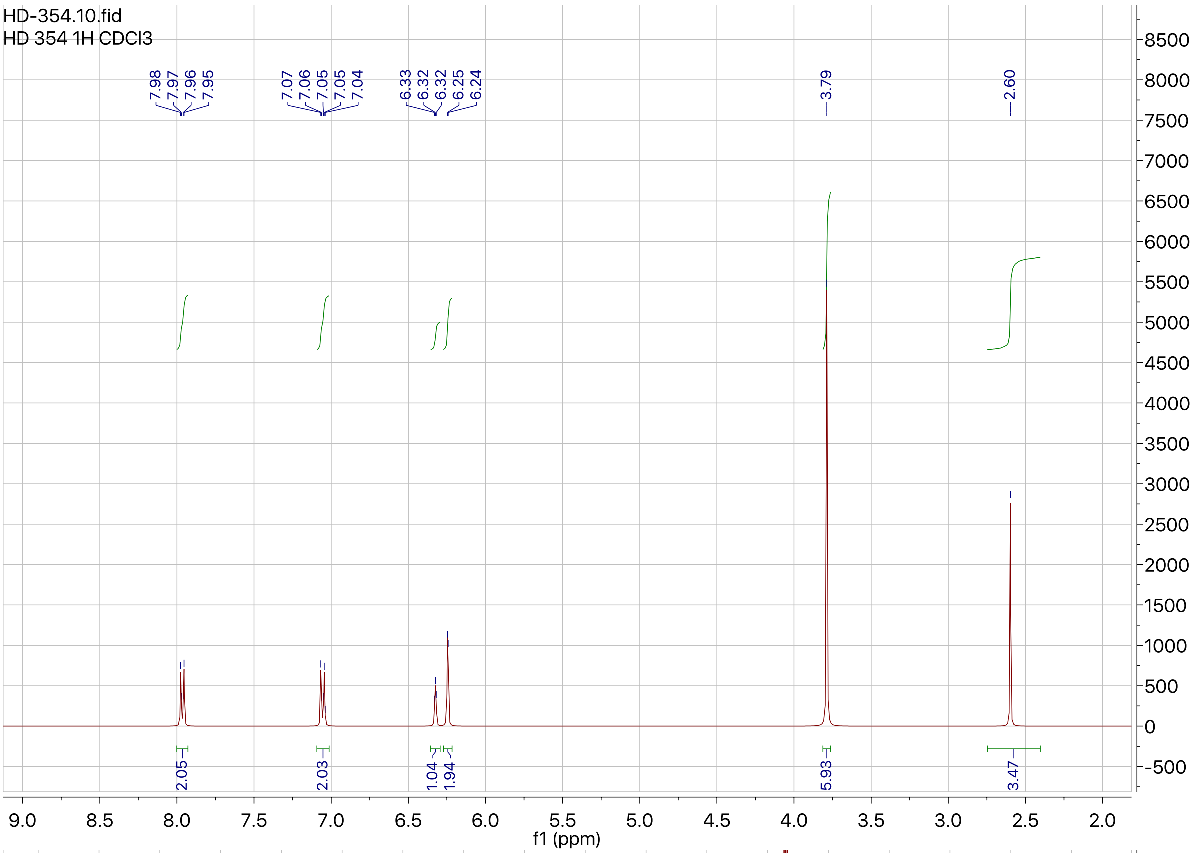

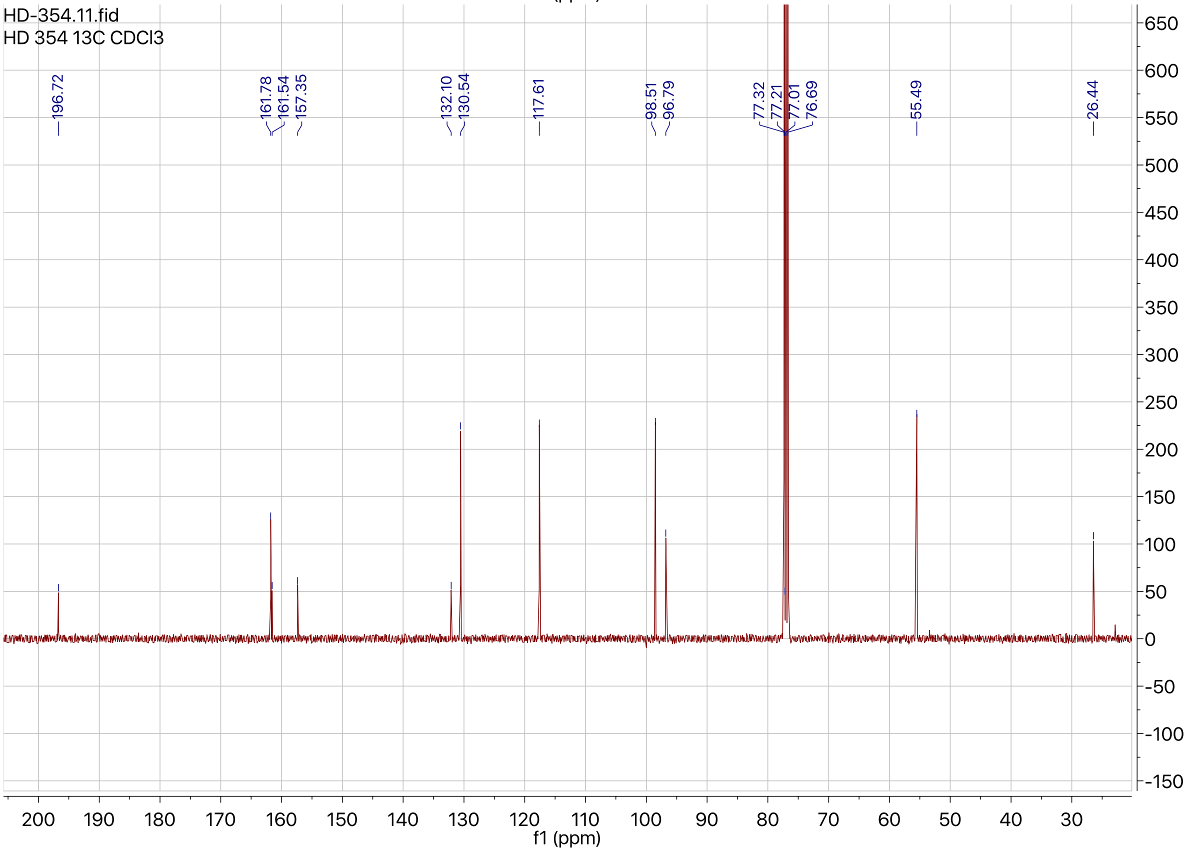

1-(4'-Fluorophenoxy)-3,5-dimethoxybenzene (31)

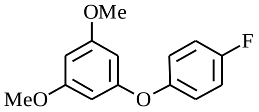

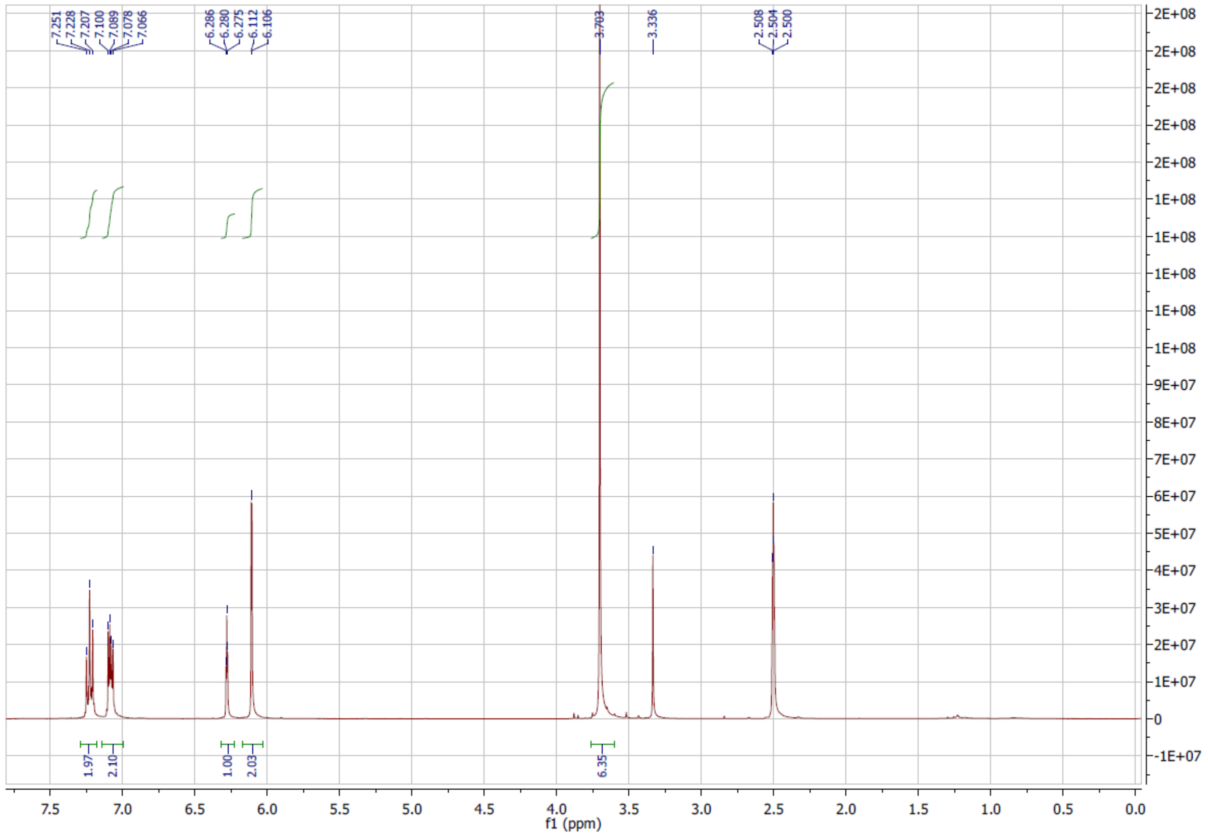

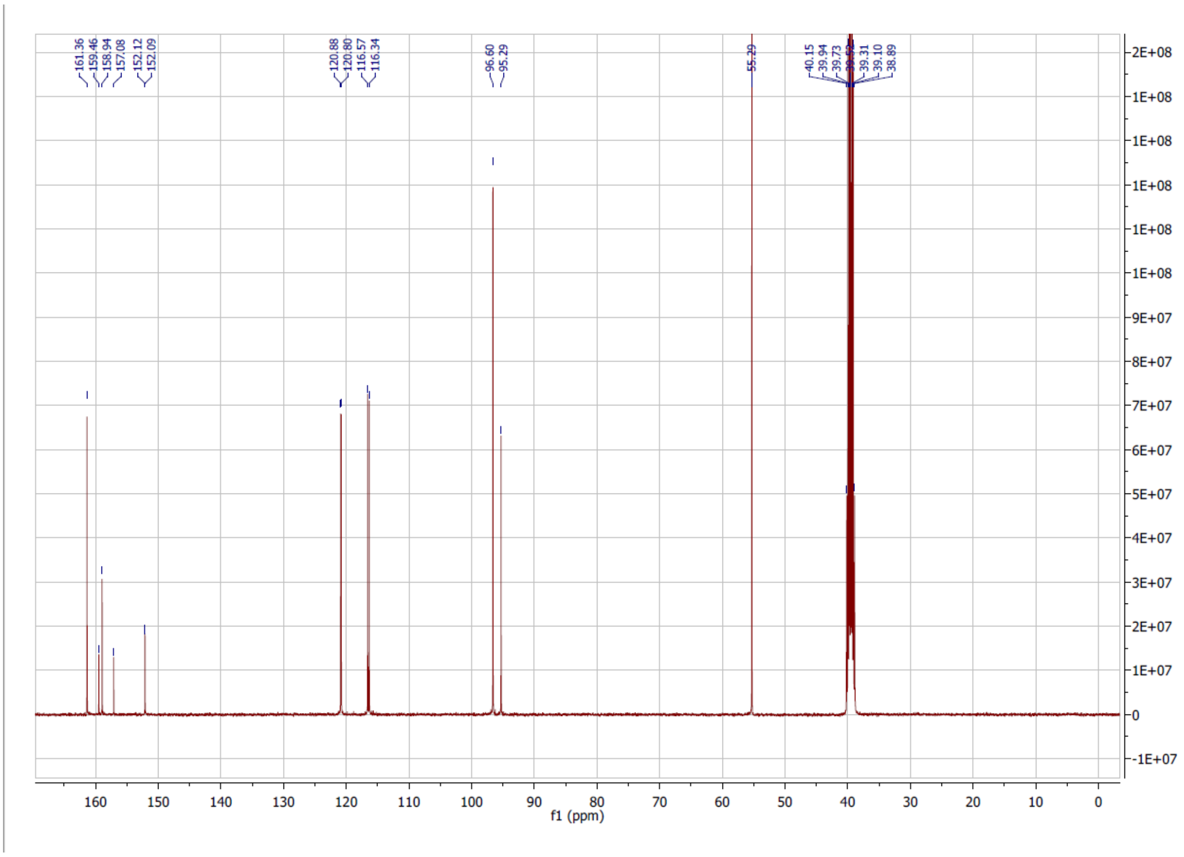

1,3-Dimethoxy-5-[4′-(trifluoromethyl)phenoxy]benzene (32)

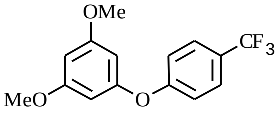

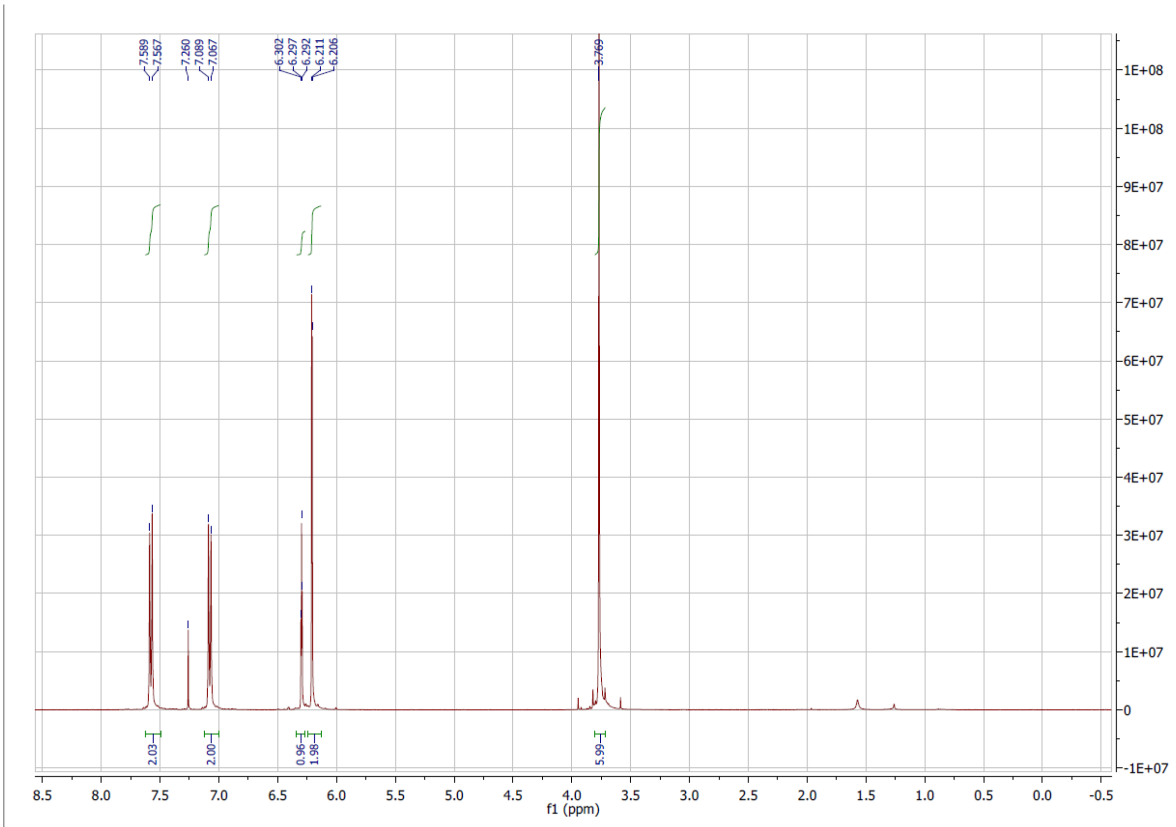

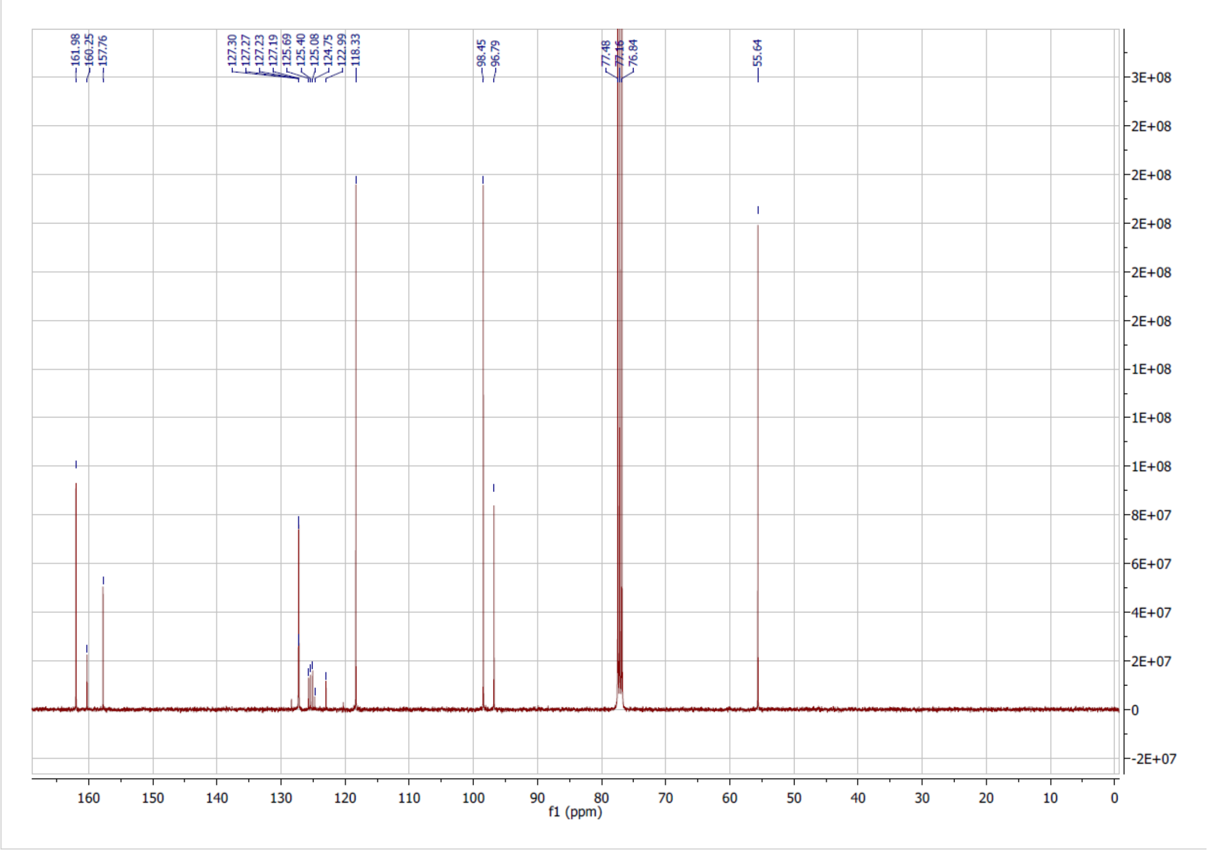

1-(3,7,9-Trimethoxydibenzo[*b*,*d*]furan-2-yl)ethanone (33)

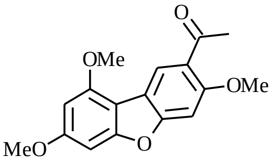

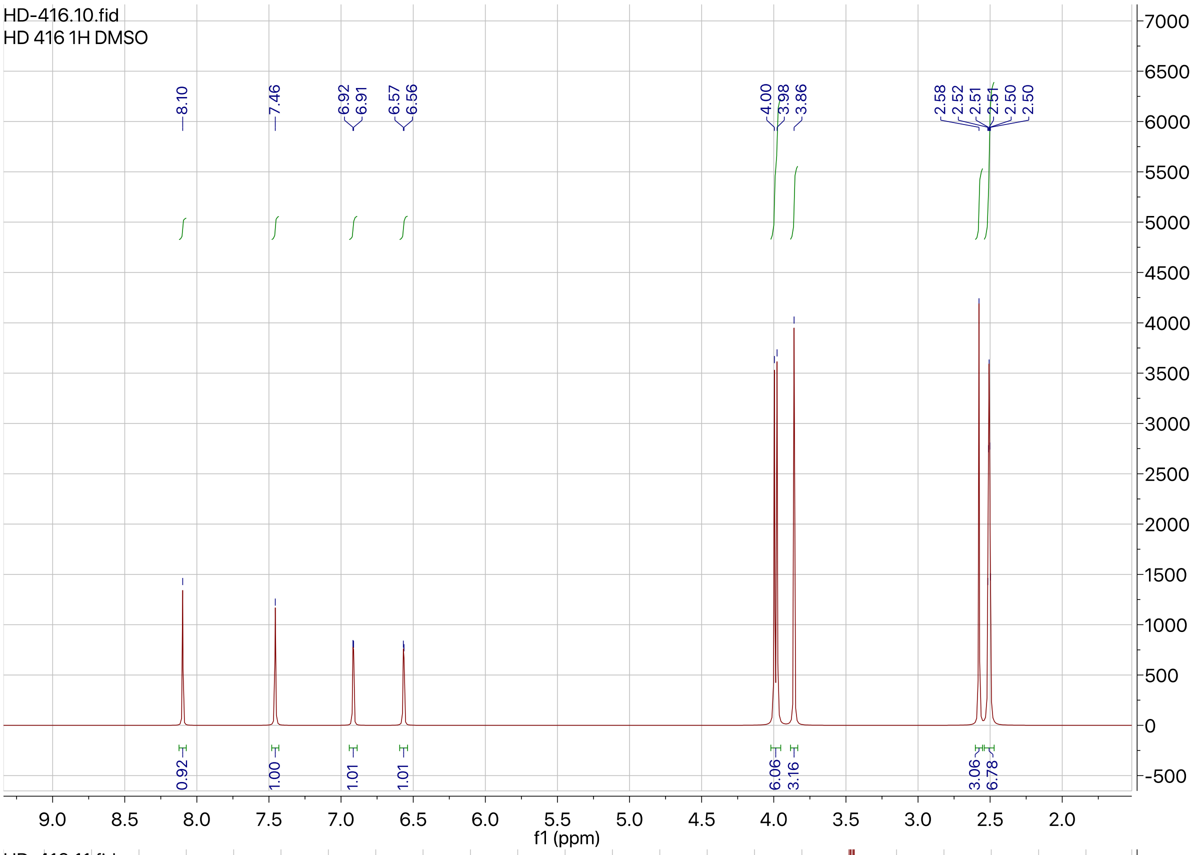

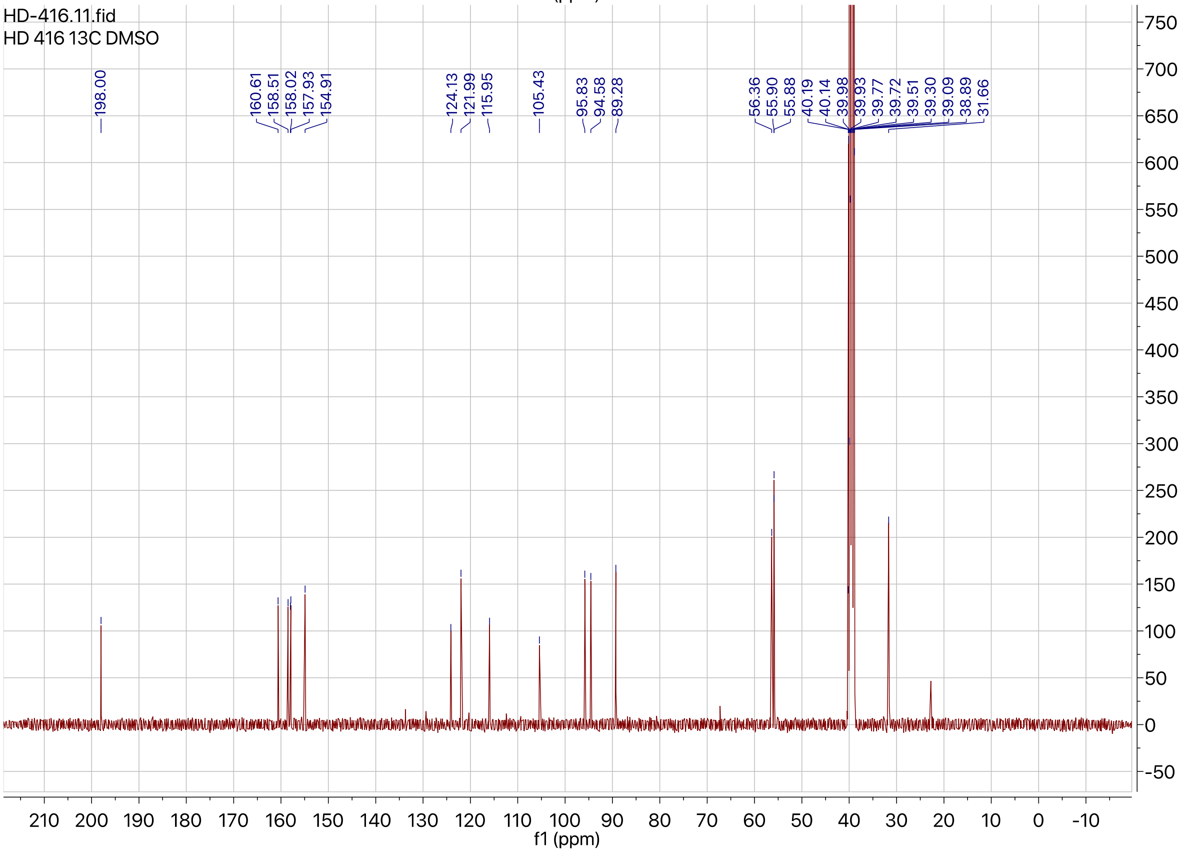

1,3,7-Trimethoxydibenzo[*b*,*d*]furan (34)

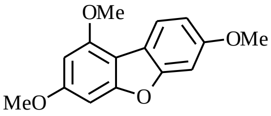

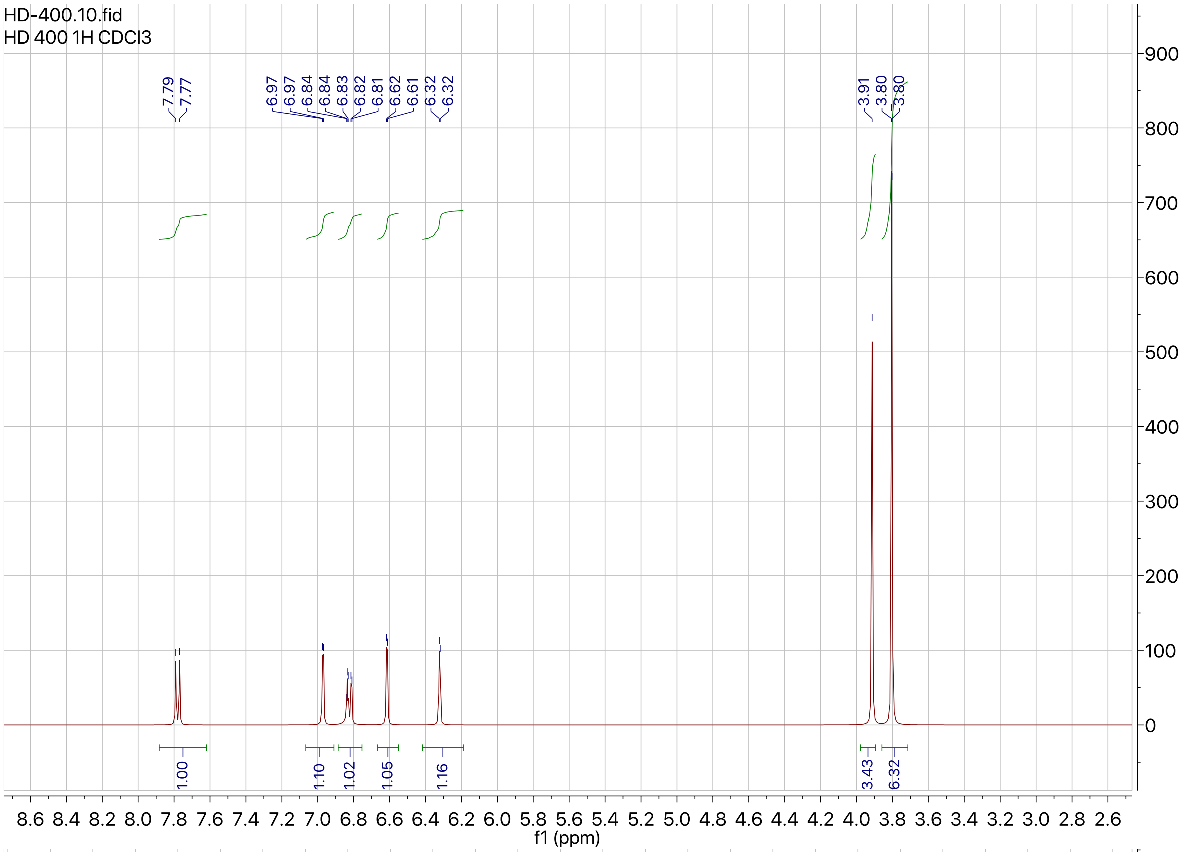

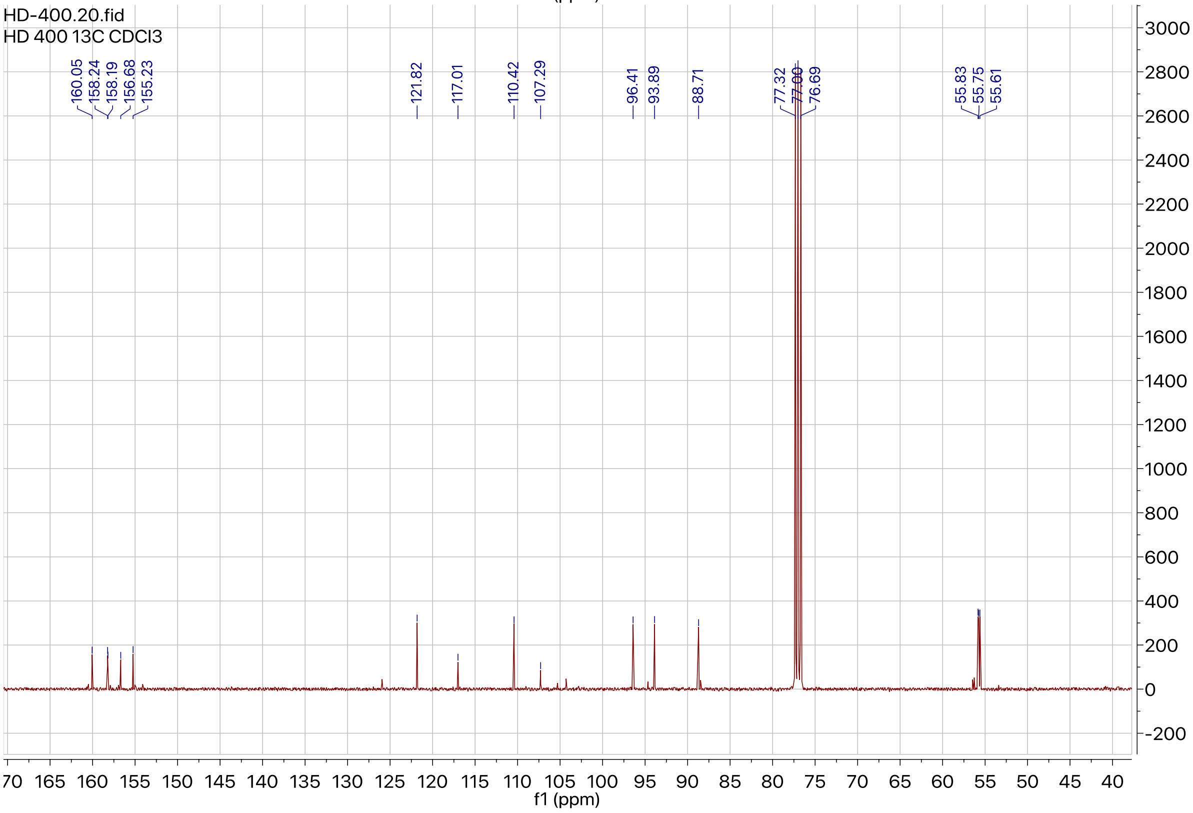

1-(7,9-Dimethoxydibenzo[*b*,*d*]furan-2-yl)ethanone (35)

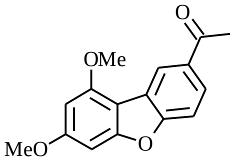

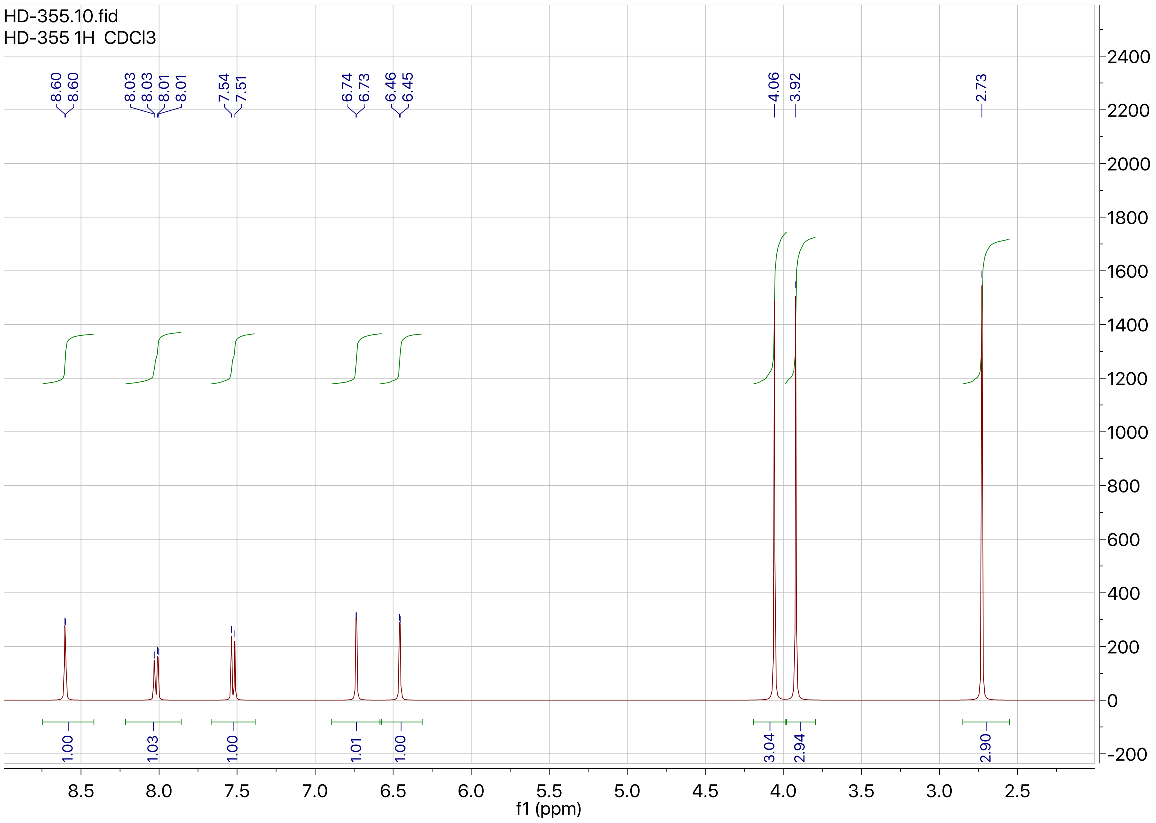

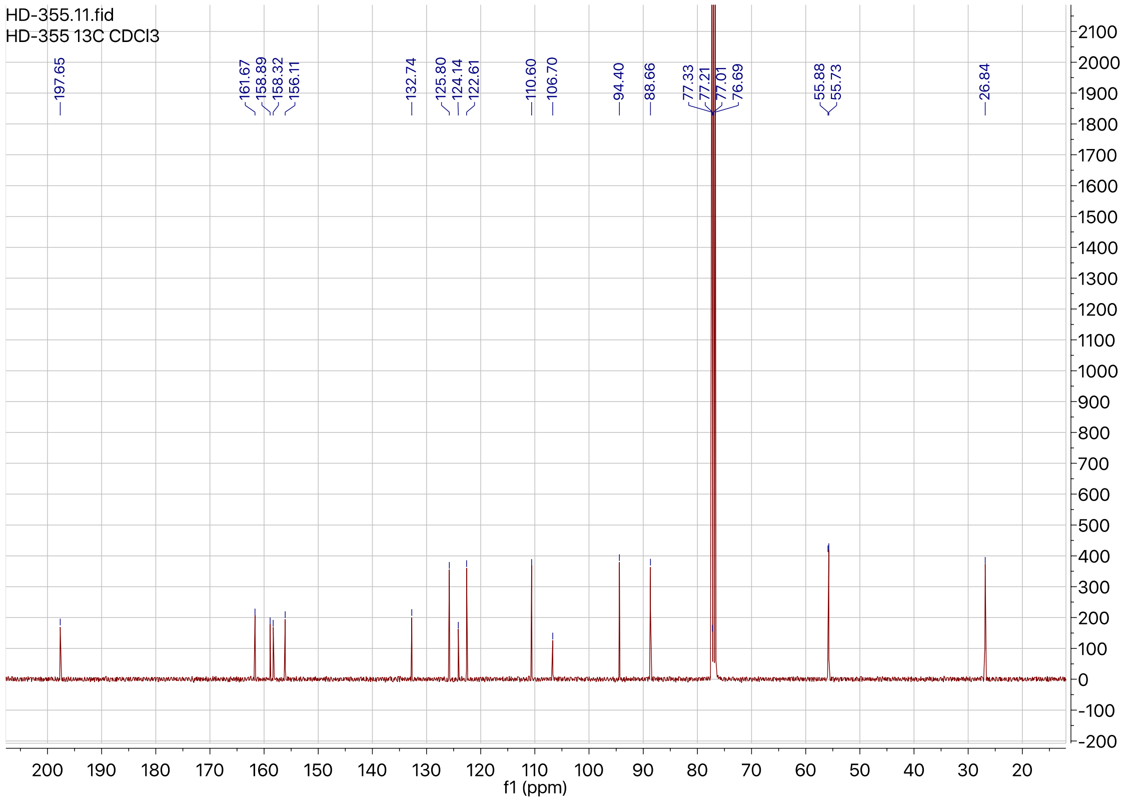

8-Fluoro-1,3-dimethoxydibenzo[*b*,*d*]furan (36)

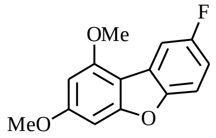

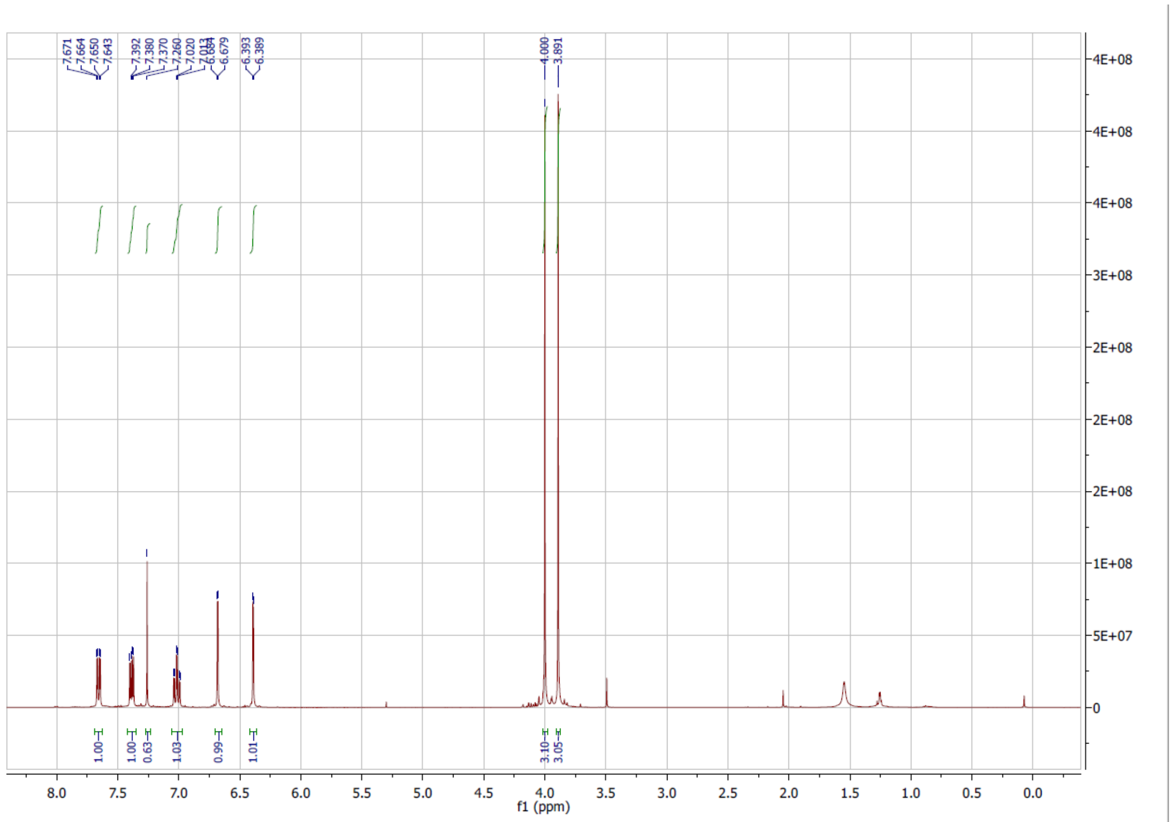

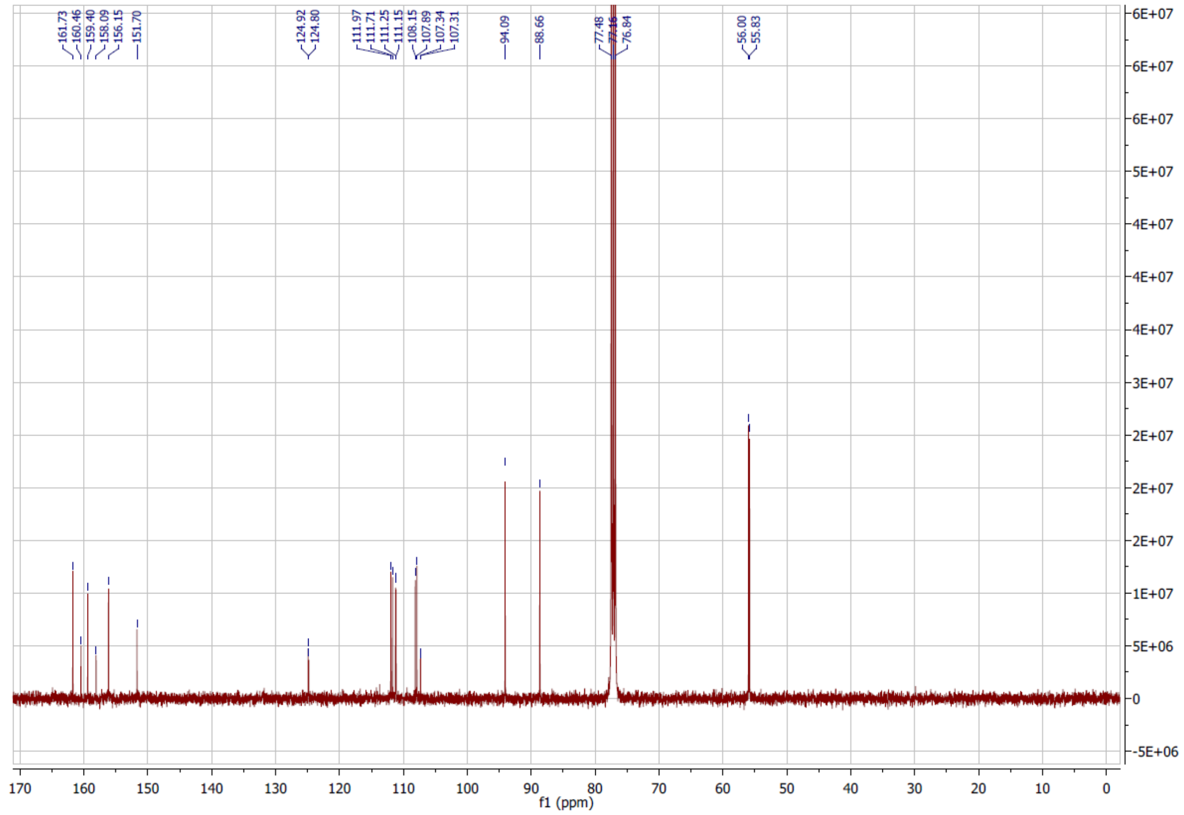

1,3-Dimethoxy-8-(trifluoromethyl)dibenzo[*b,d*]furan (37)

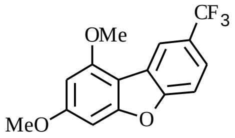

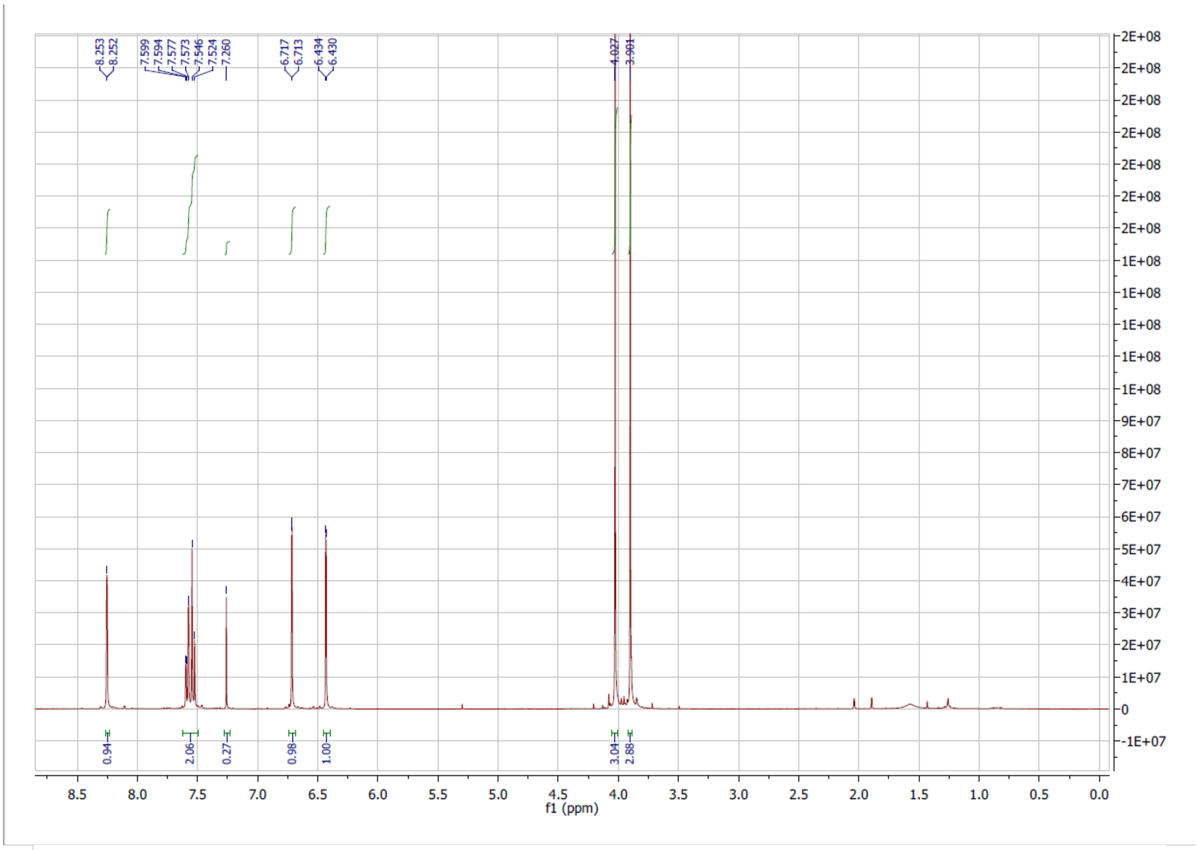

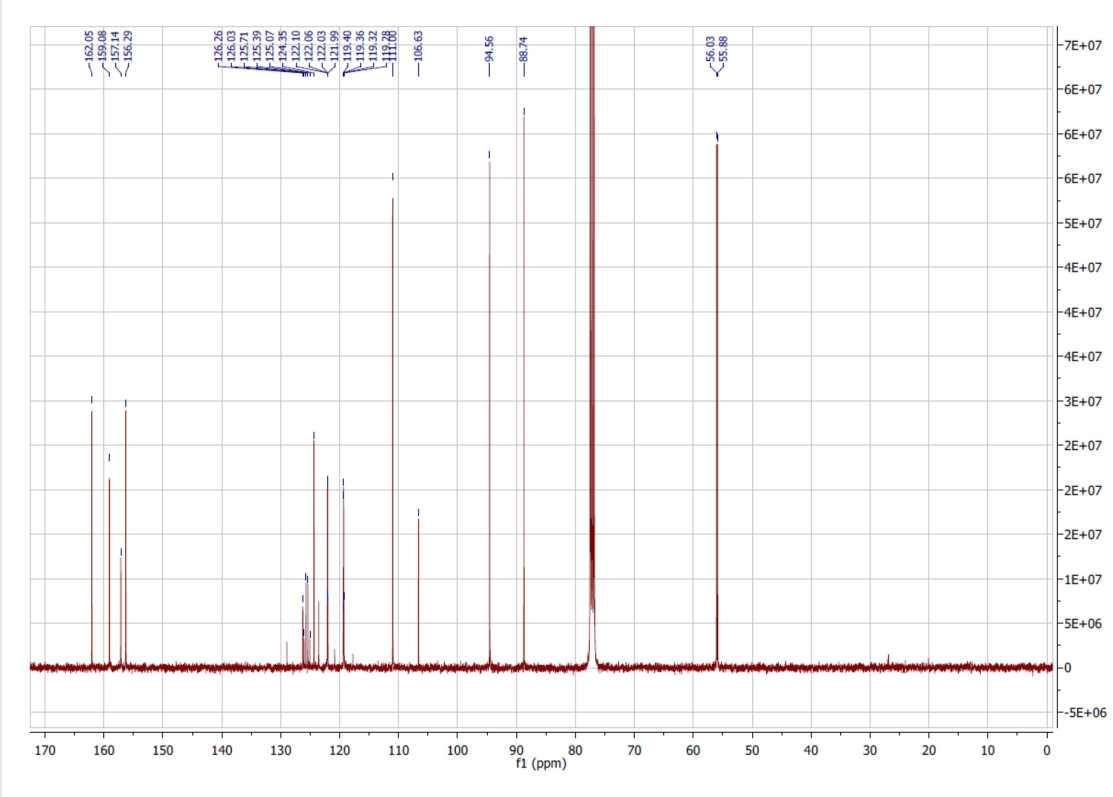

8-Acetyl-1,3,7-trimethoxydibenzo[*b*,*d*]furan-4-carboxamide (38)

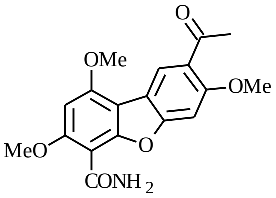

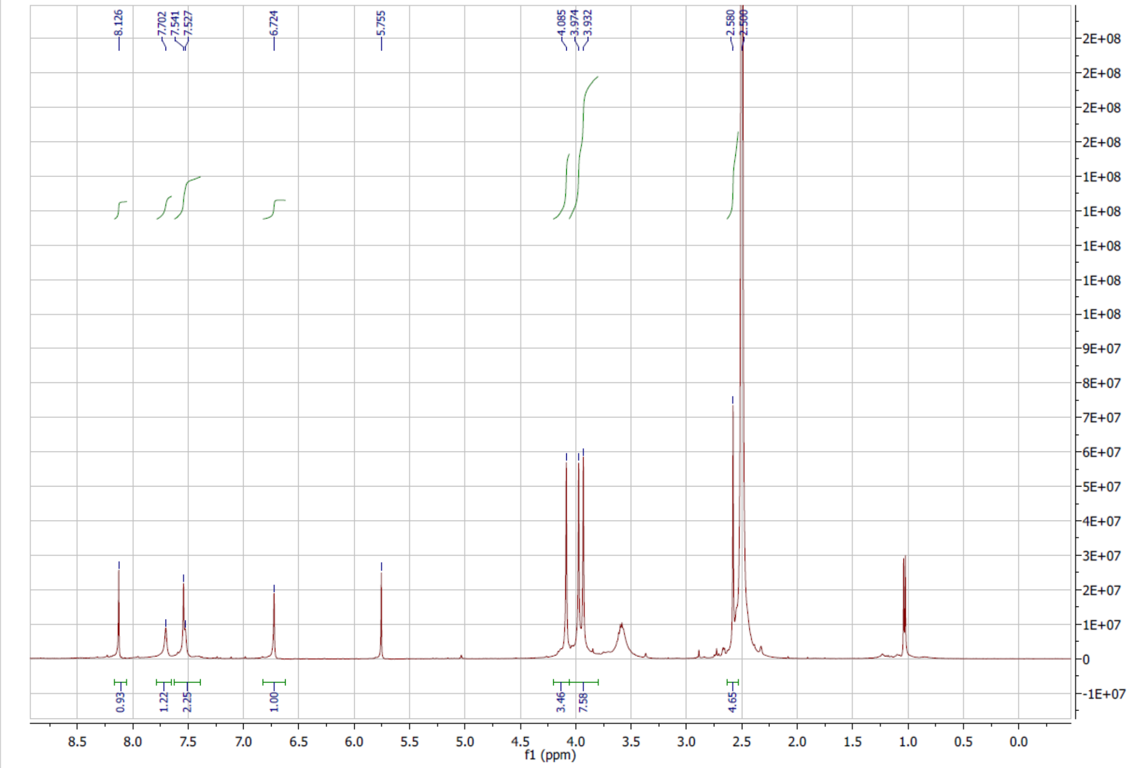

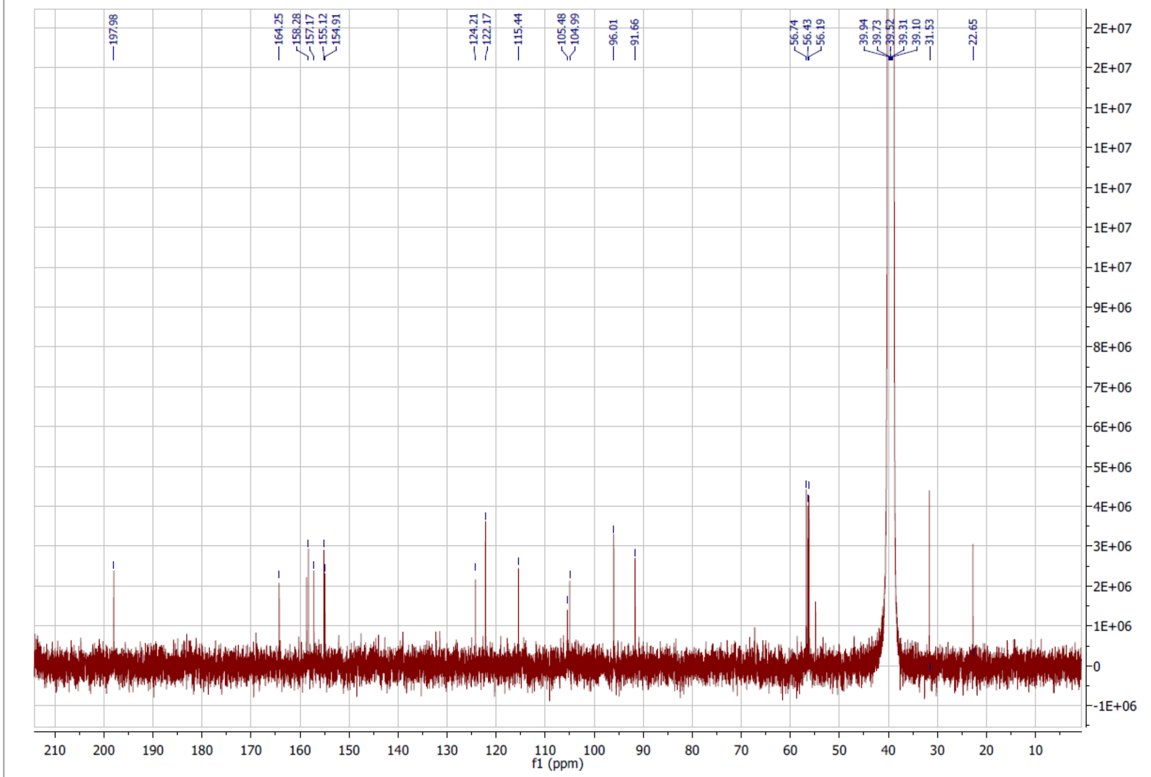

1,3,7-Trimethoxydibenzo[*b*,*d*]furan-4-carboxamide (39)

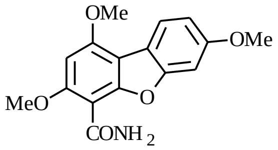

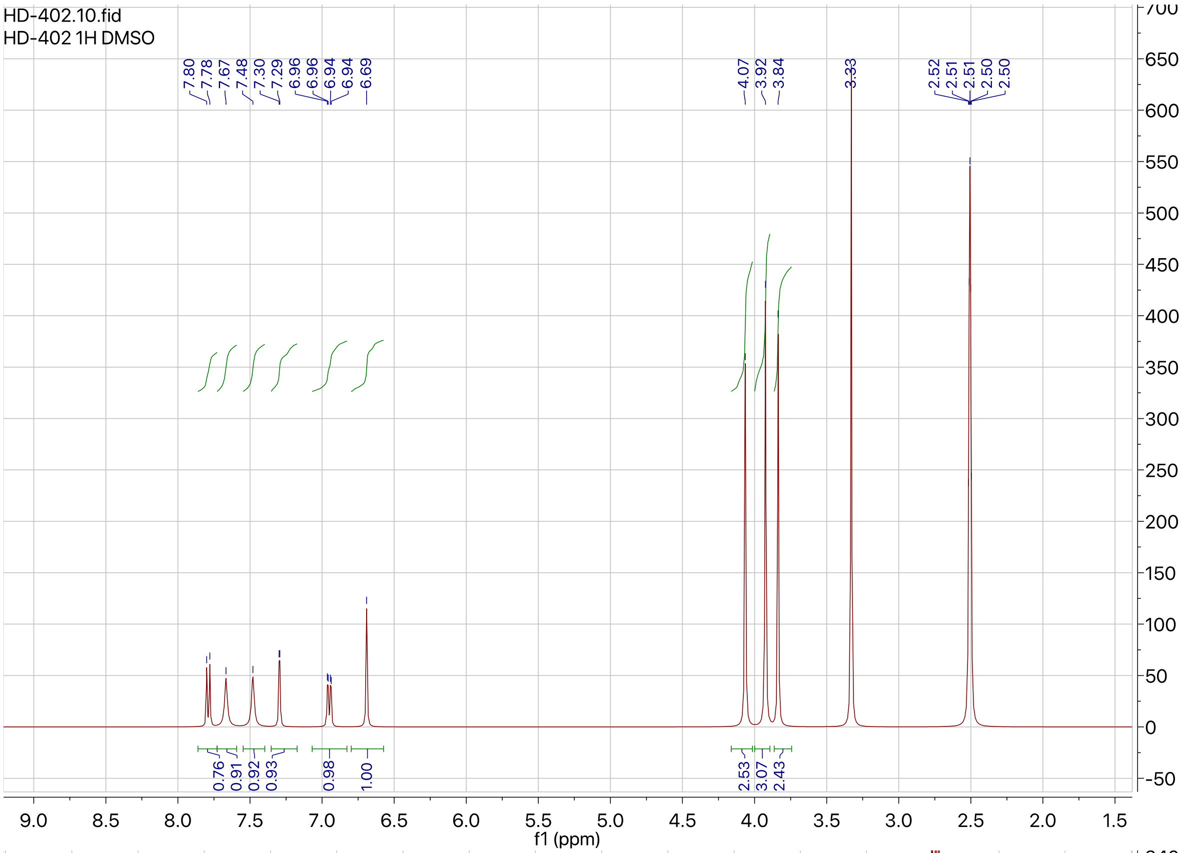

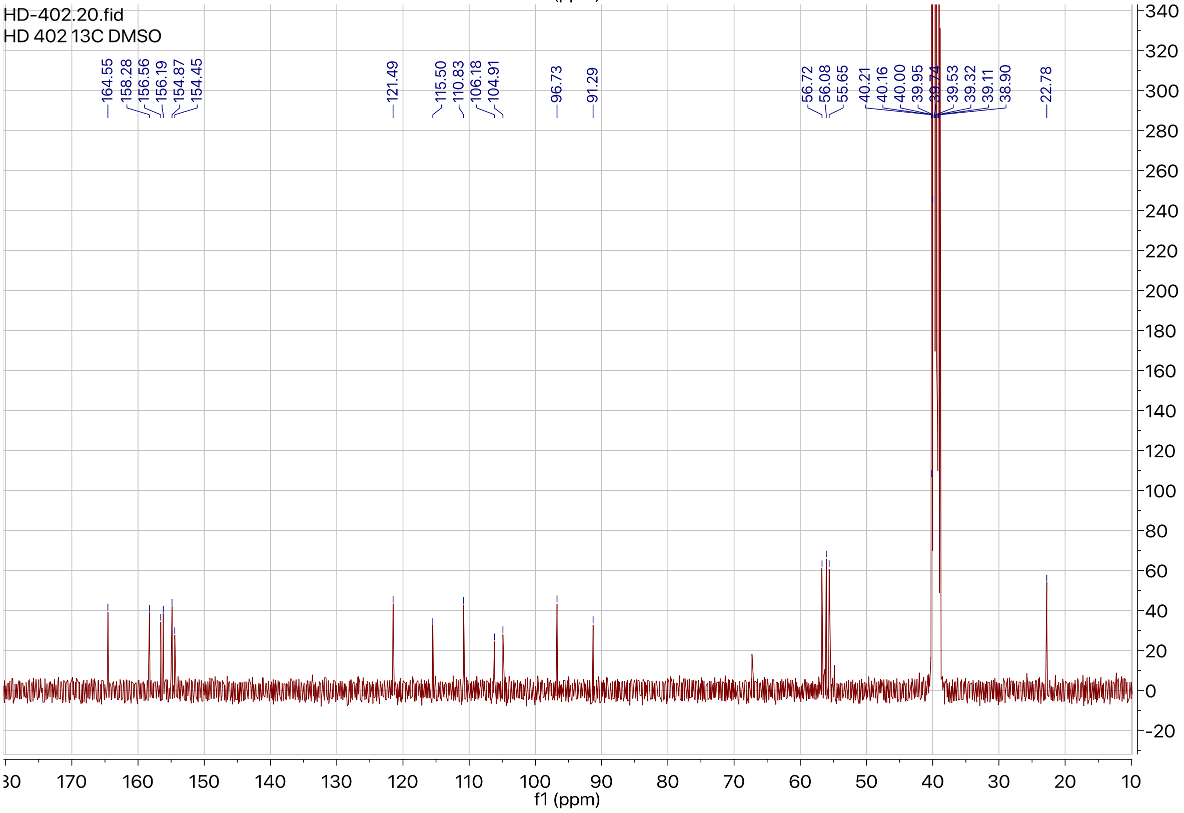

8-Acetyl-1,3-dimethoxydibenzo[*b*,*d*]furan-4-carboxamide (40)

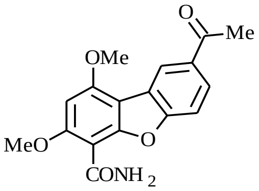

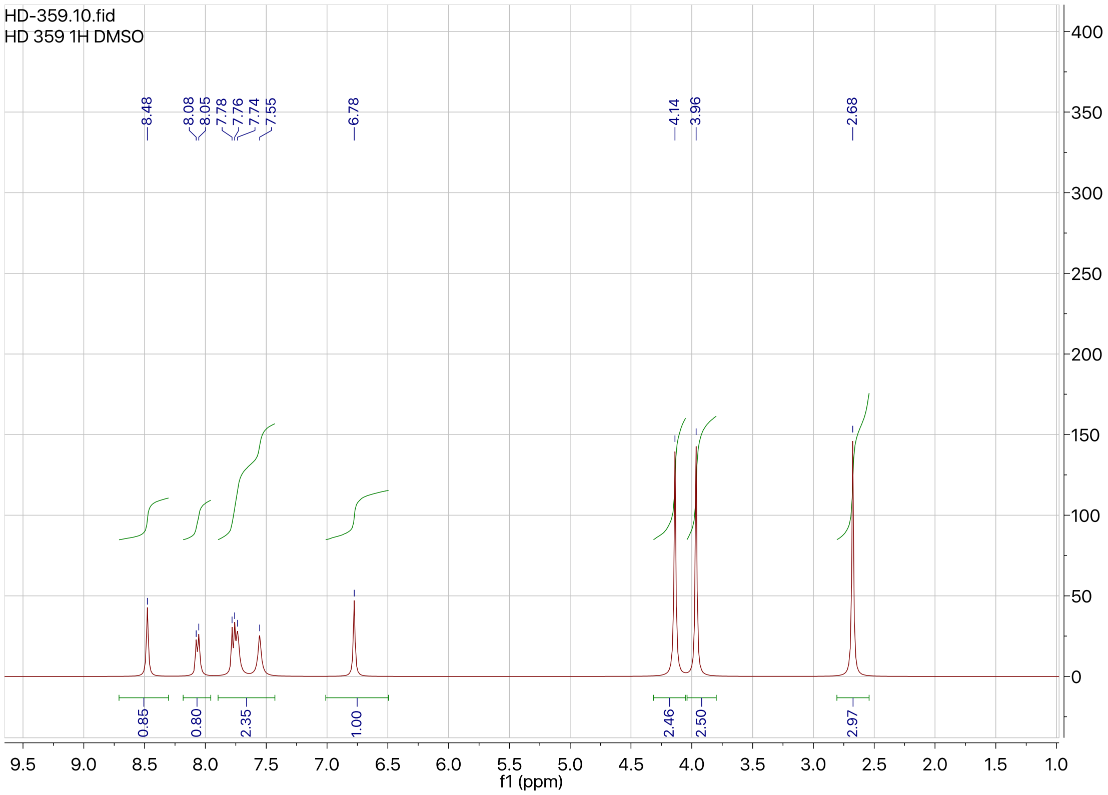

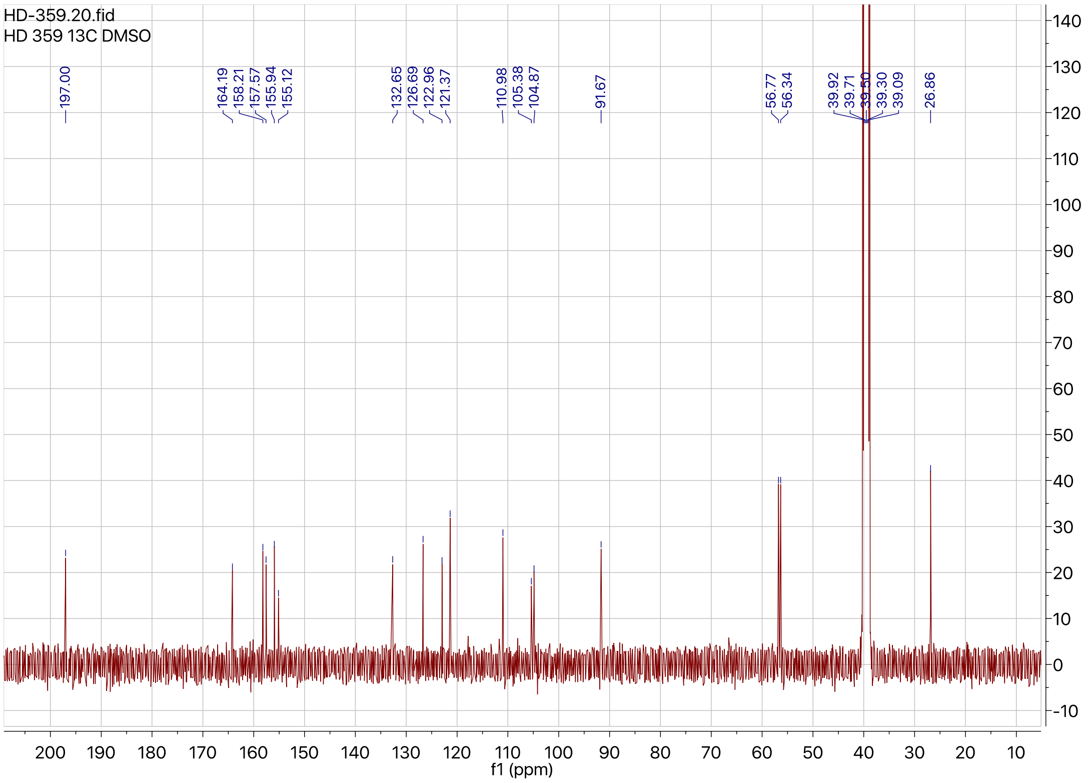

8-Fluoro-1,3-dimethoxydibenzo[*b*,*d*]furan-4-carboxamide (41)

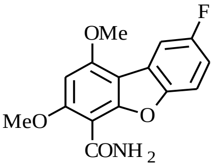

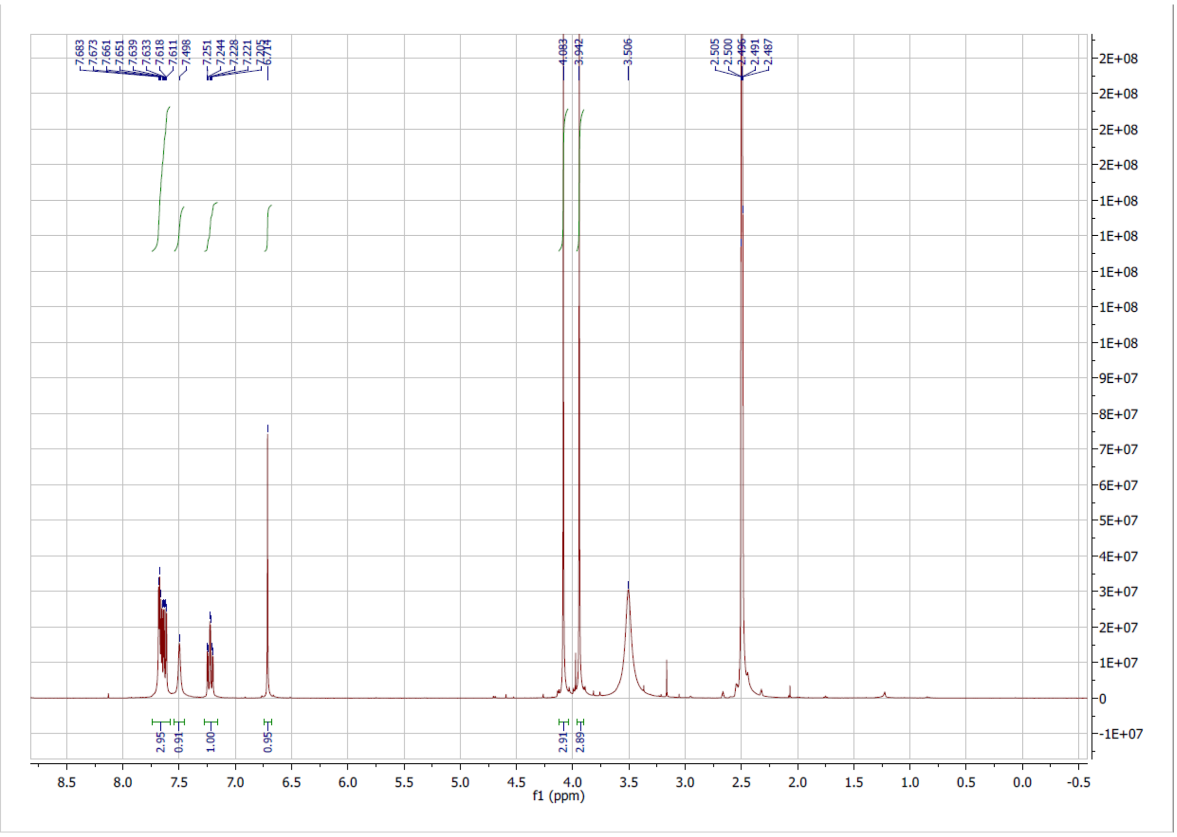

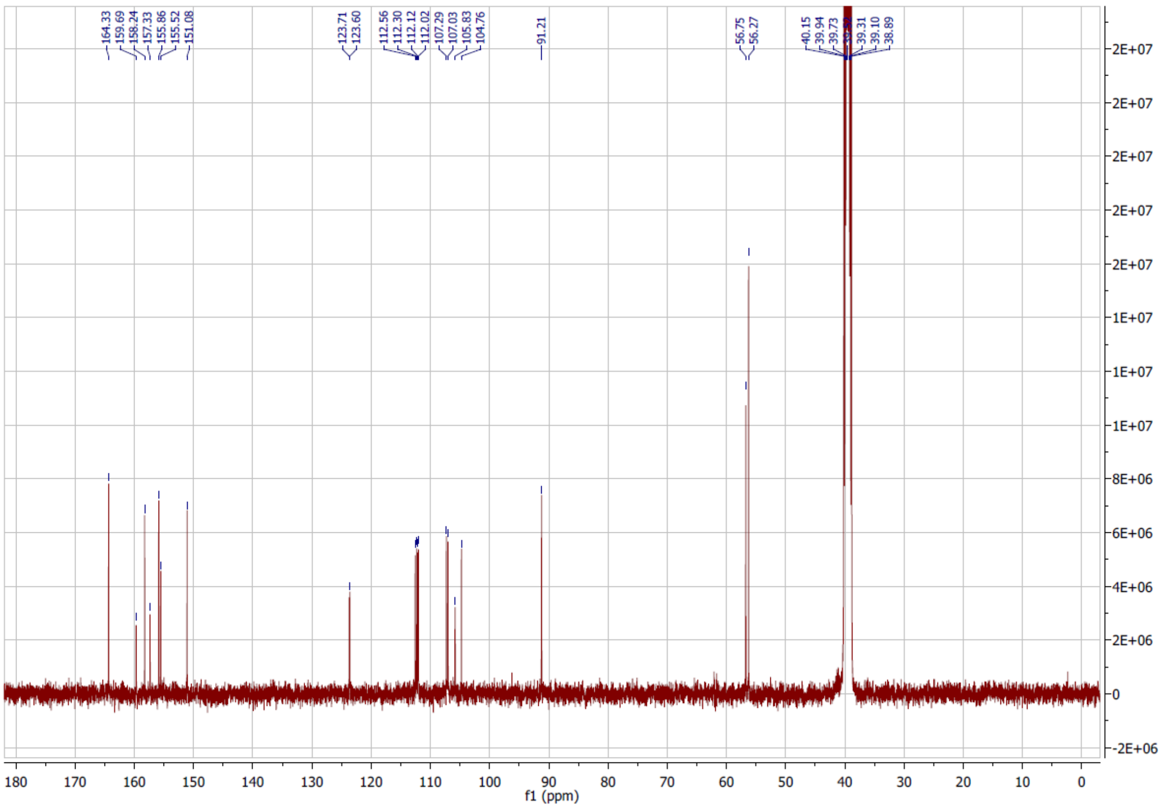

1,3-Dimethoxy-8-(trifluoromethyl)dibenzo[*b*,*d*]furan-4-carboxamide (42)

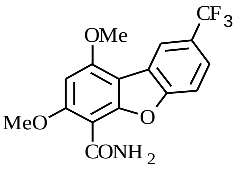

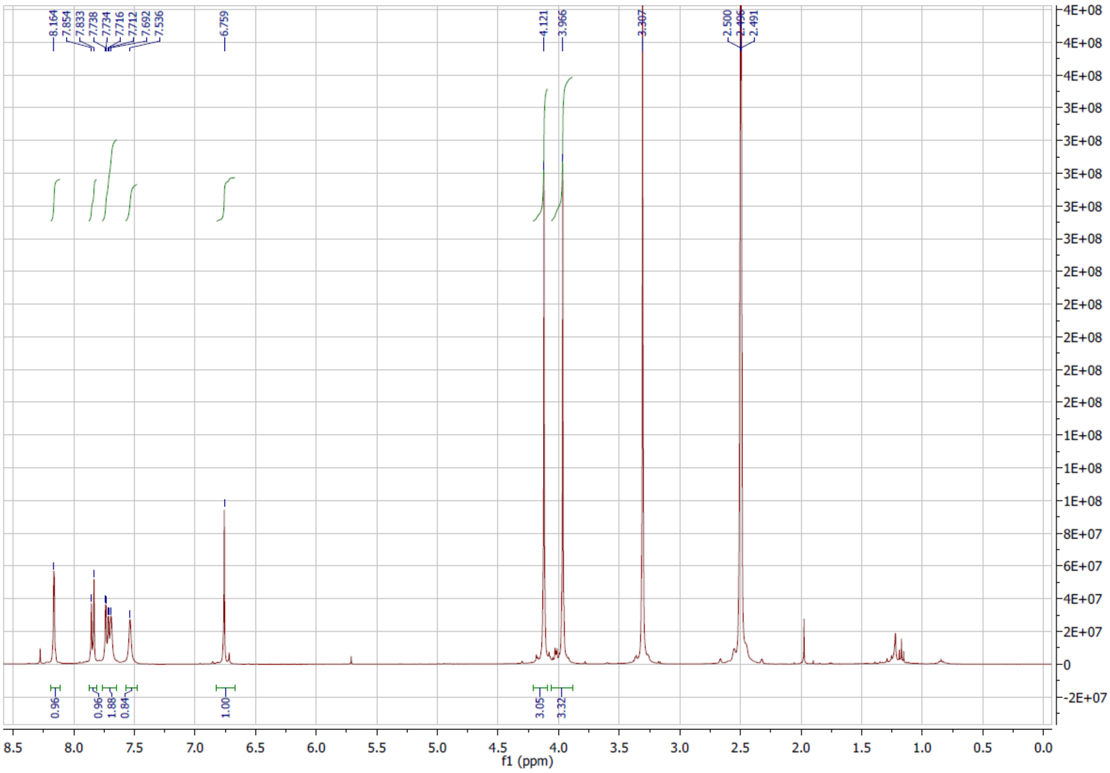

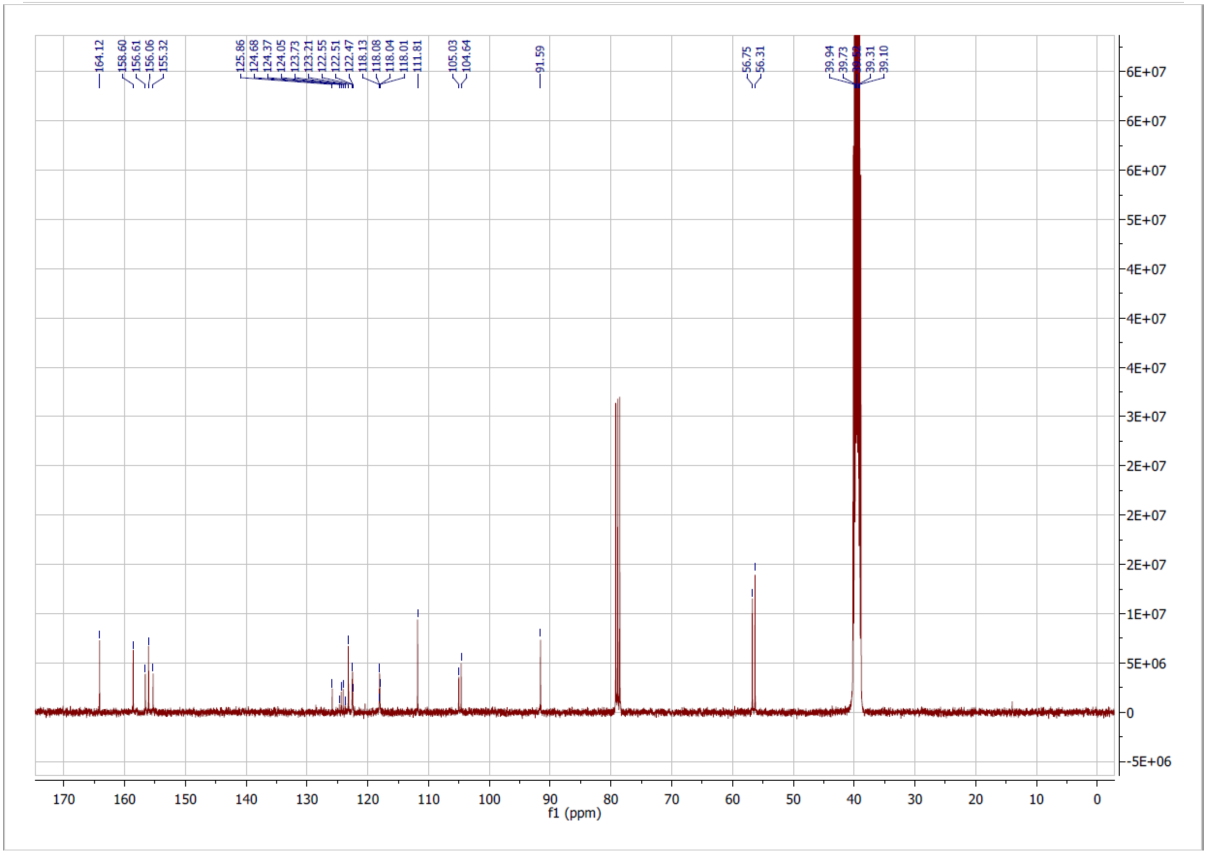

8-Acetyl-1,3,7-trihydroxydibenzo[*b*,*d*]furan-4-carboxamide (43)

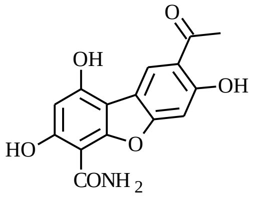

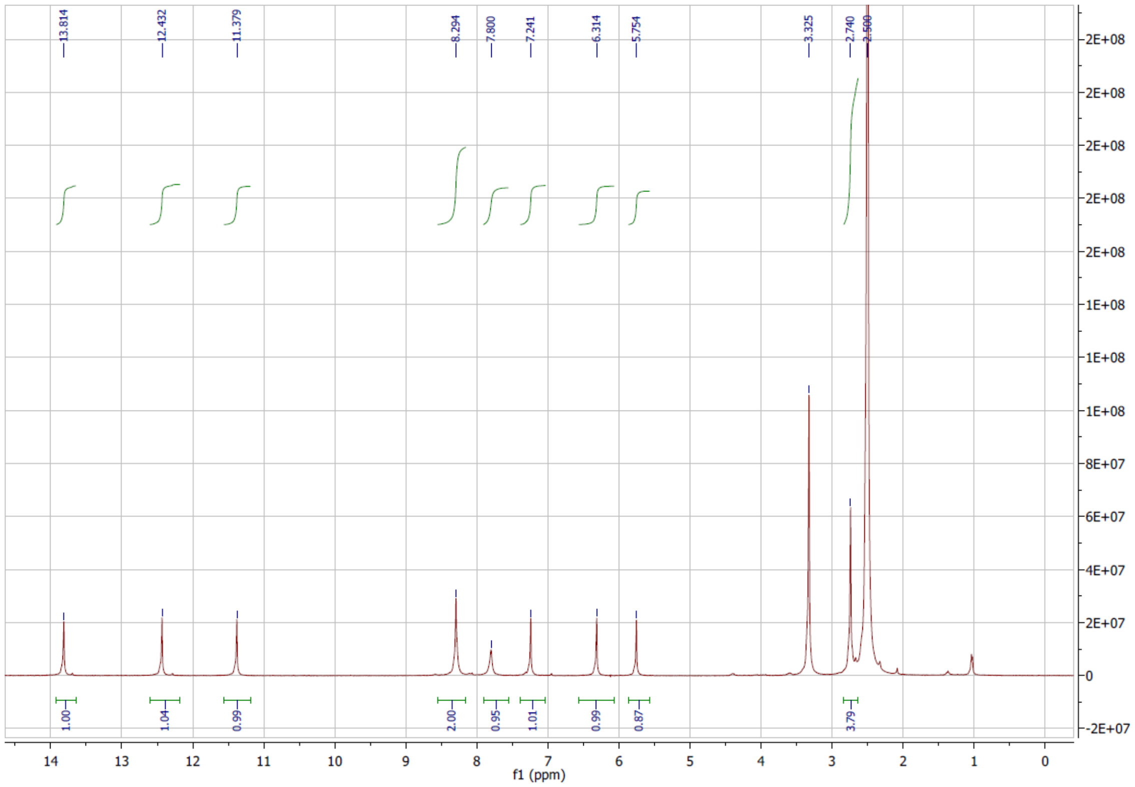

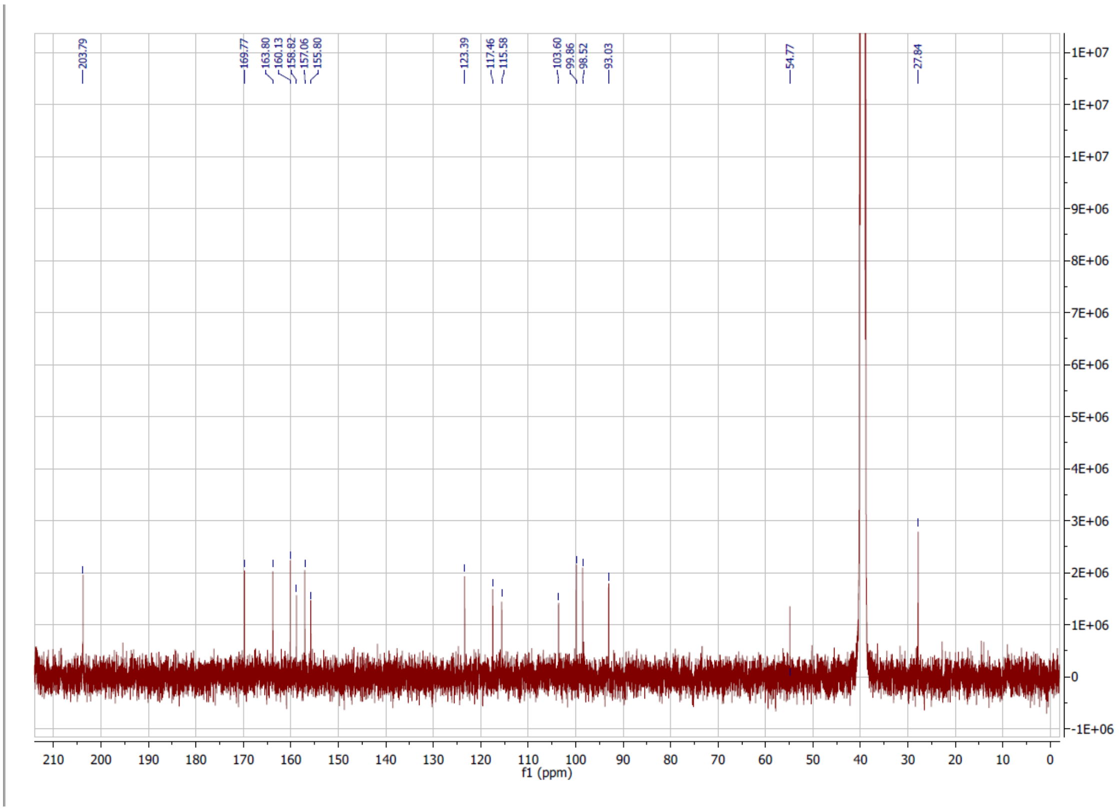

1,3,7-Trihydroxydibenzo[*b,d*]furan-4-carboxamide (44)

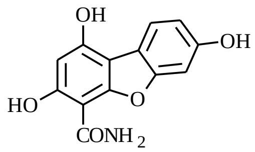

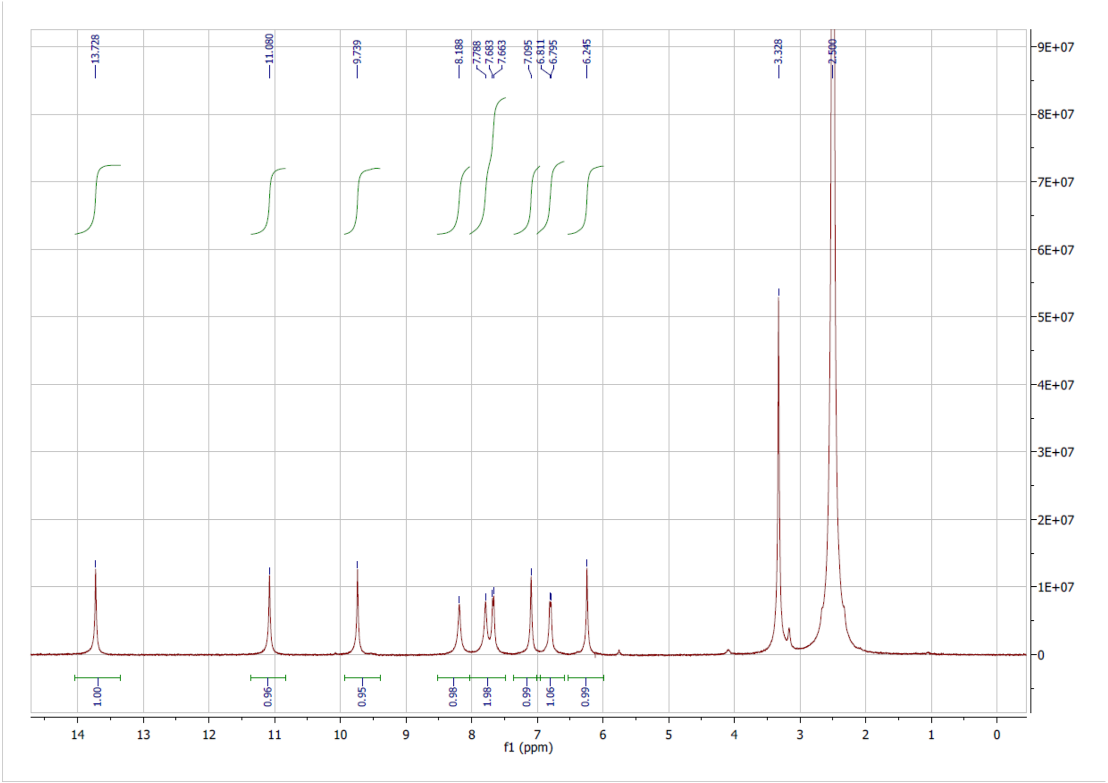

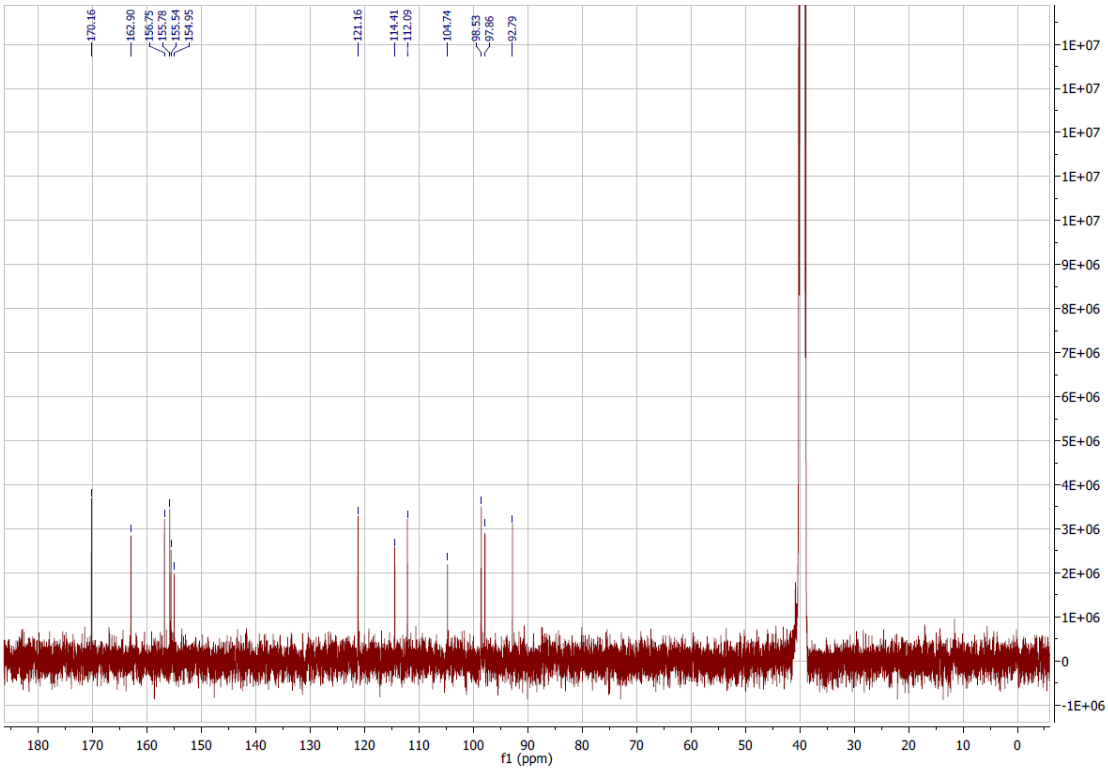

8-Acetyl-1,3-dihydroxydibenzo[*b*,*d*]furan-4-carboxamide (45)

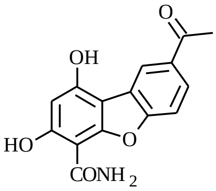

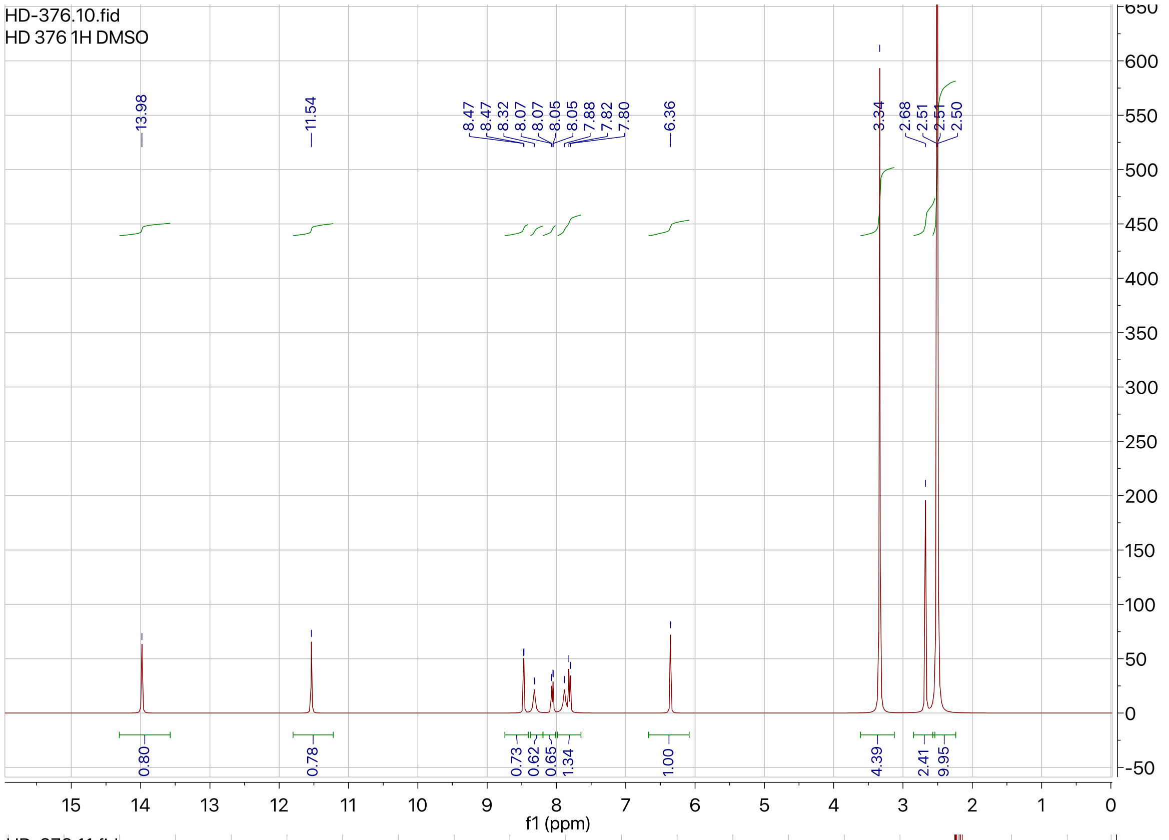

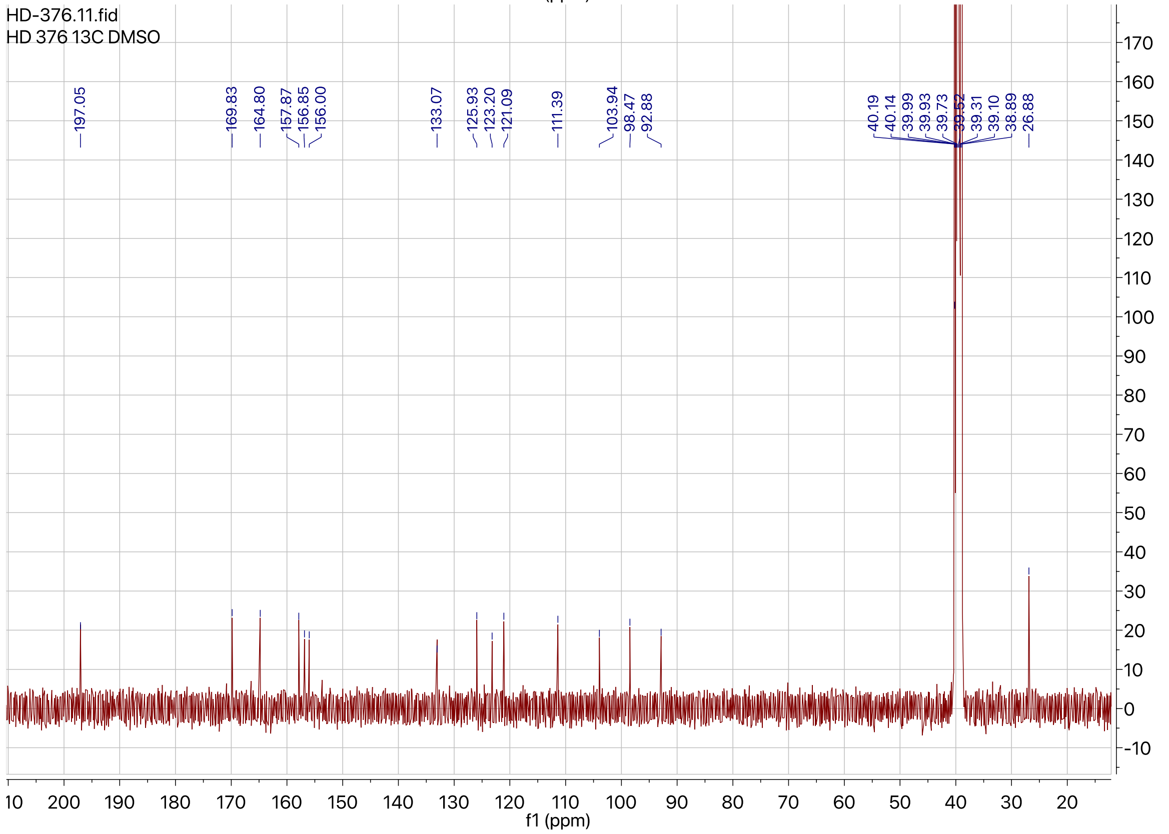

8-Fluoro-1,3-dihydroxydibenzo[*b*,*d*]furan-4-carboxamide (46)

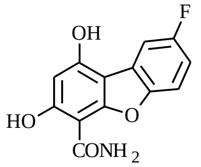

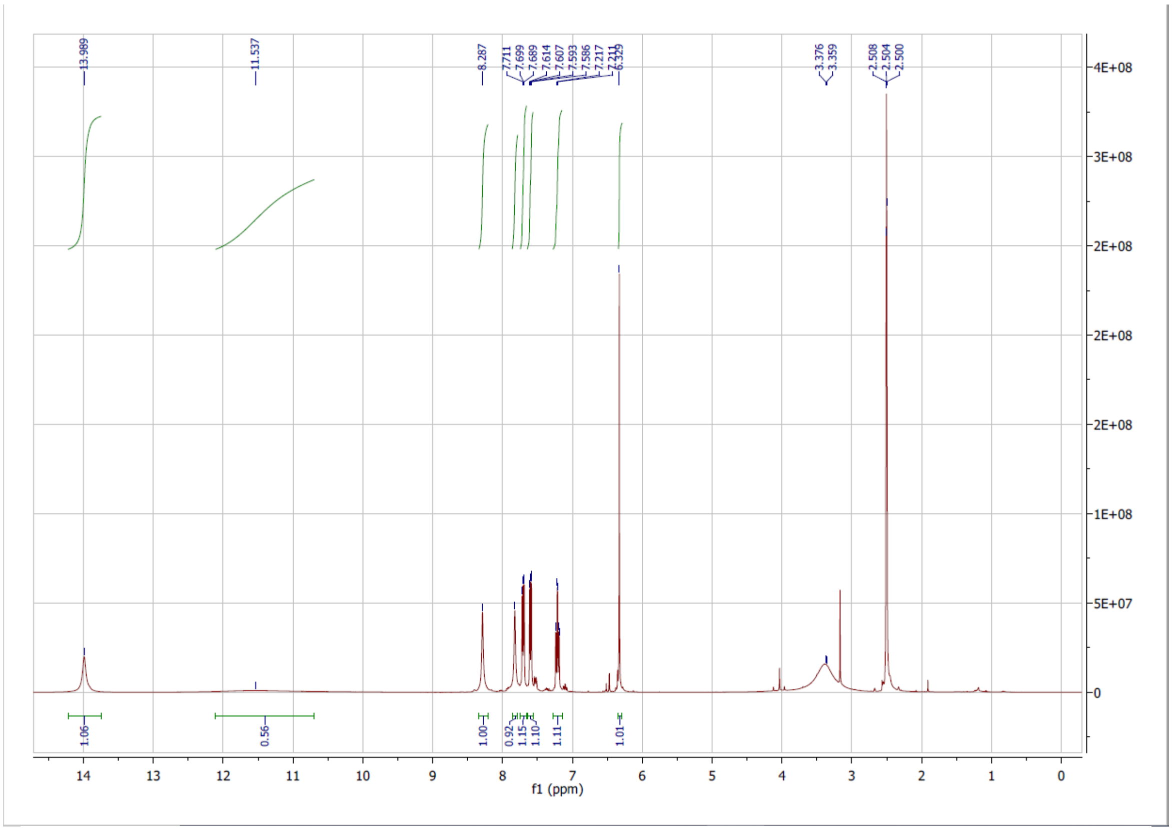

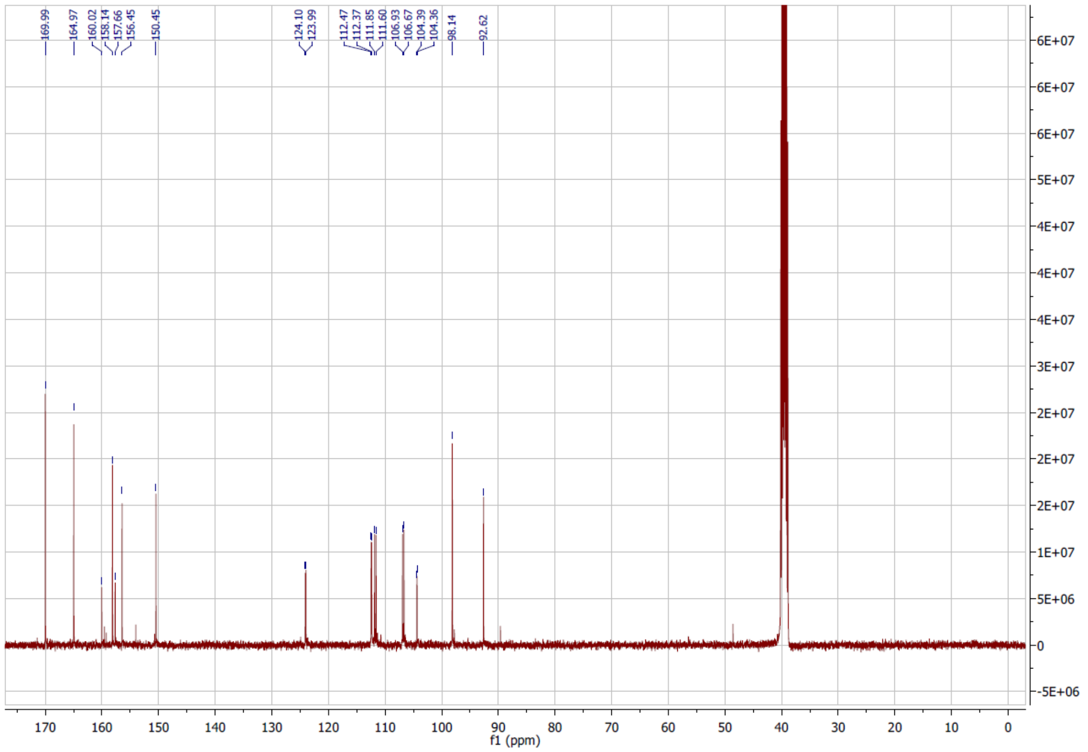

1,3-Dihydroxy-8-(trifluoromethyl)dibenzo[*b*,*d*]furan-4-carboxamide (47)

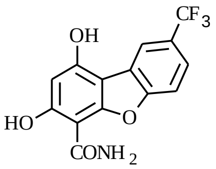

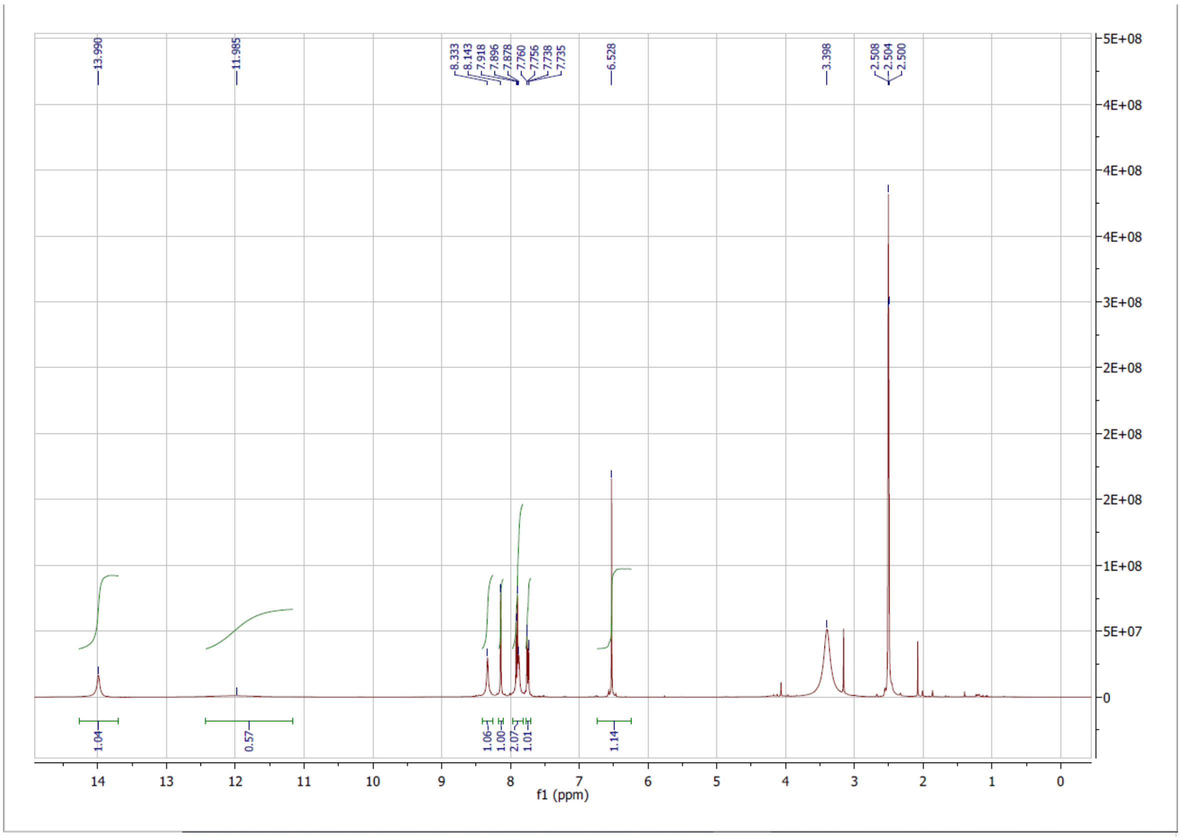

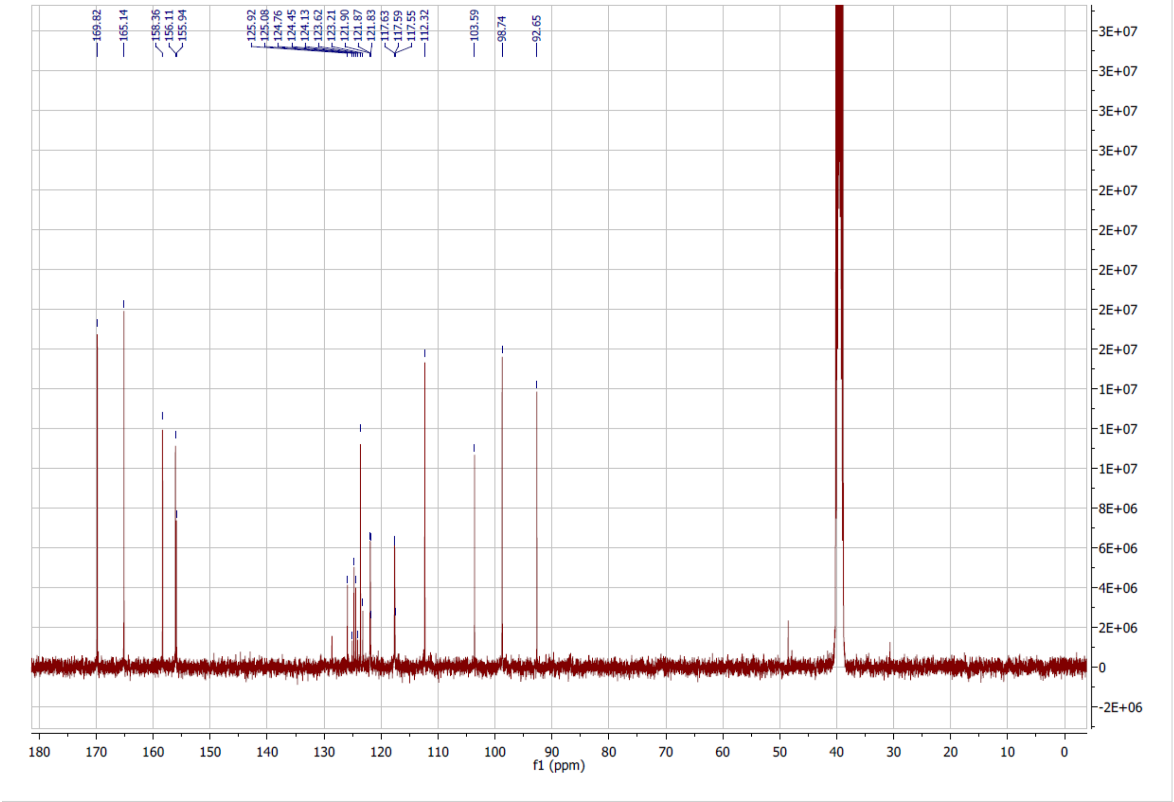

2-Hydroxy-3-iodo-4,6-dimethoxybenzamide (48)

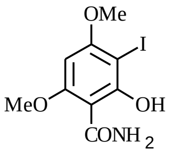

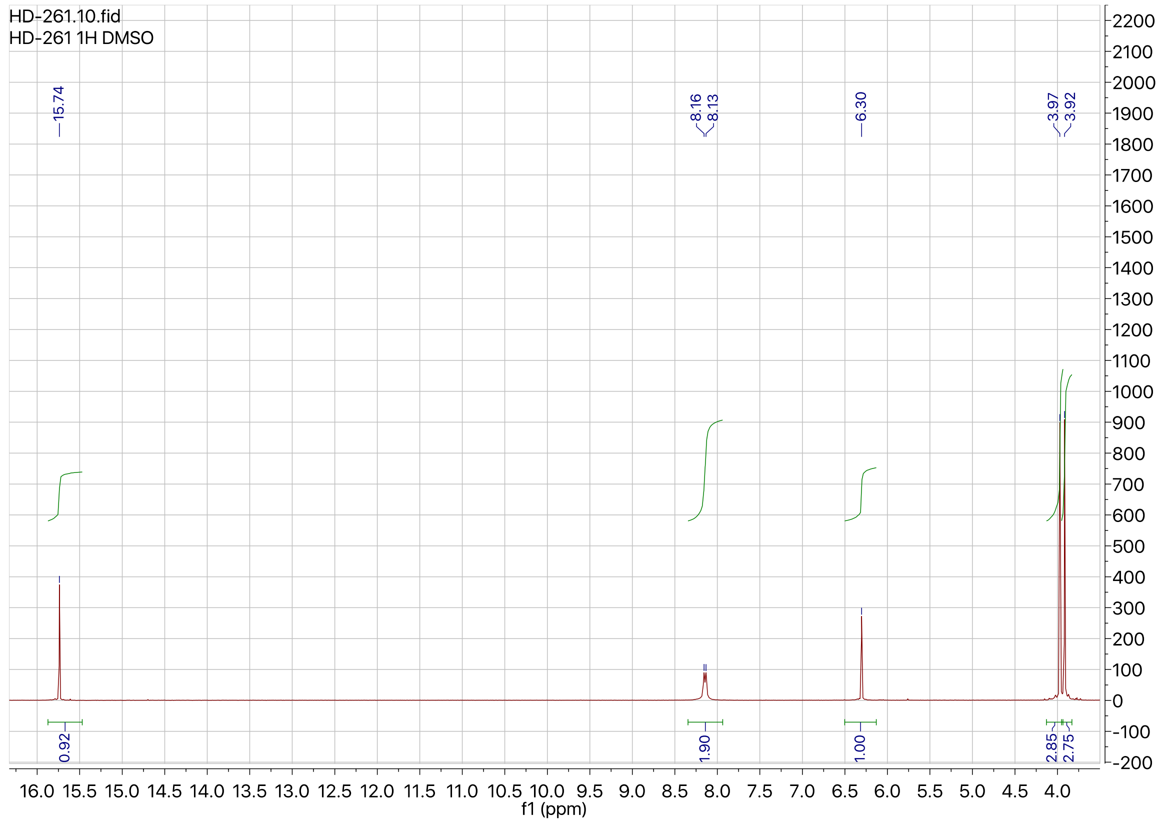

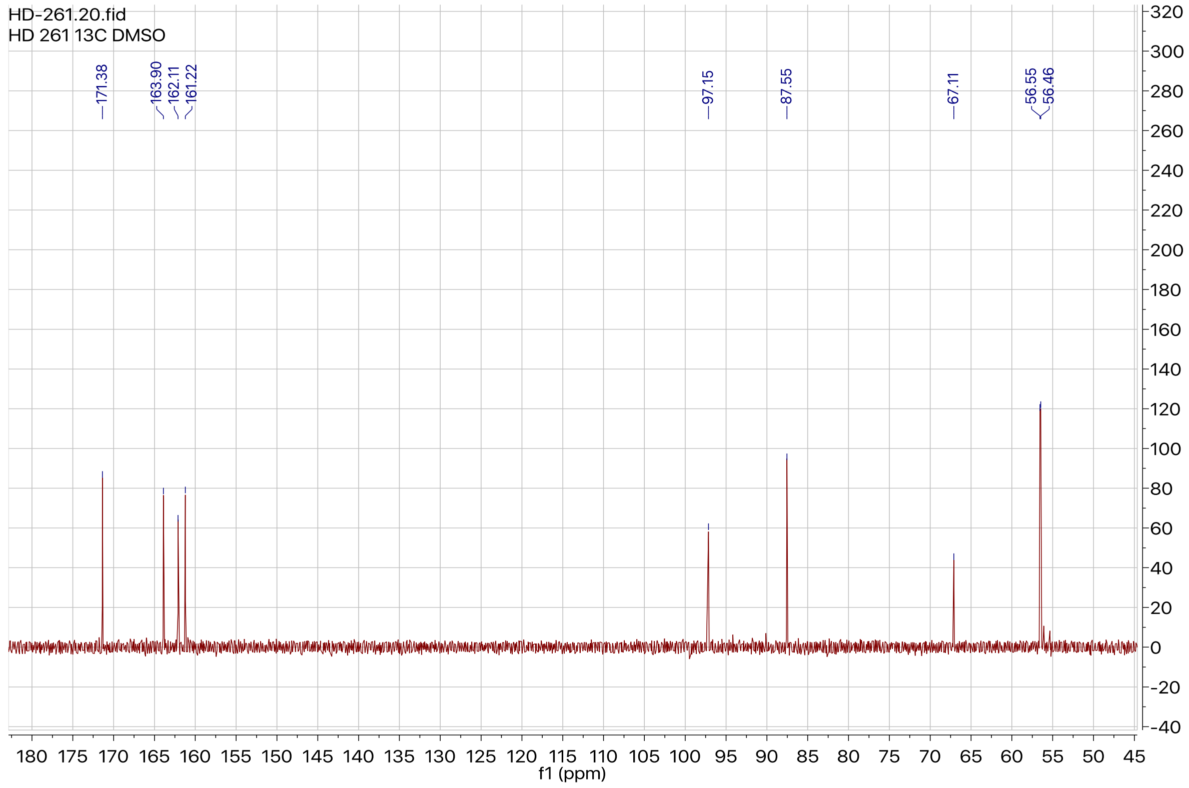

2'-Fluoro-2-hydroxy-4,6-dimethoxybiphenyl-3-carboxamide (49)

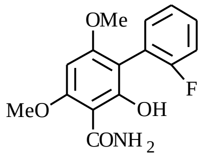

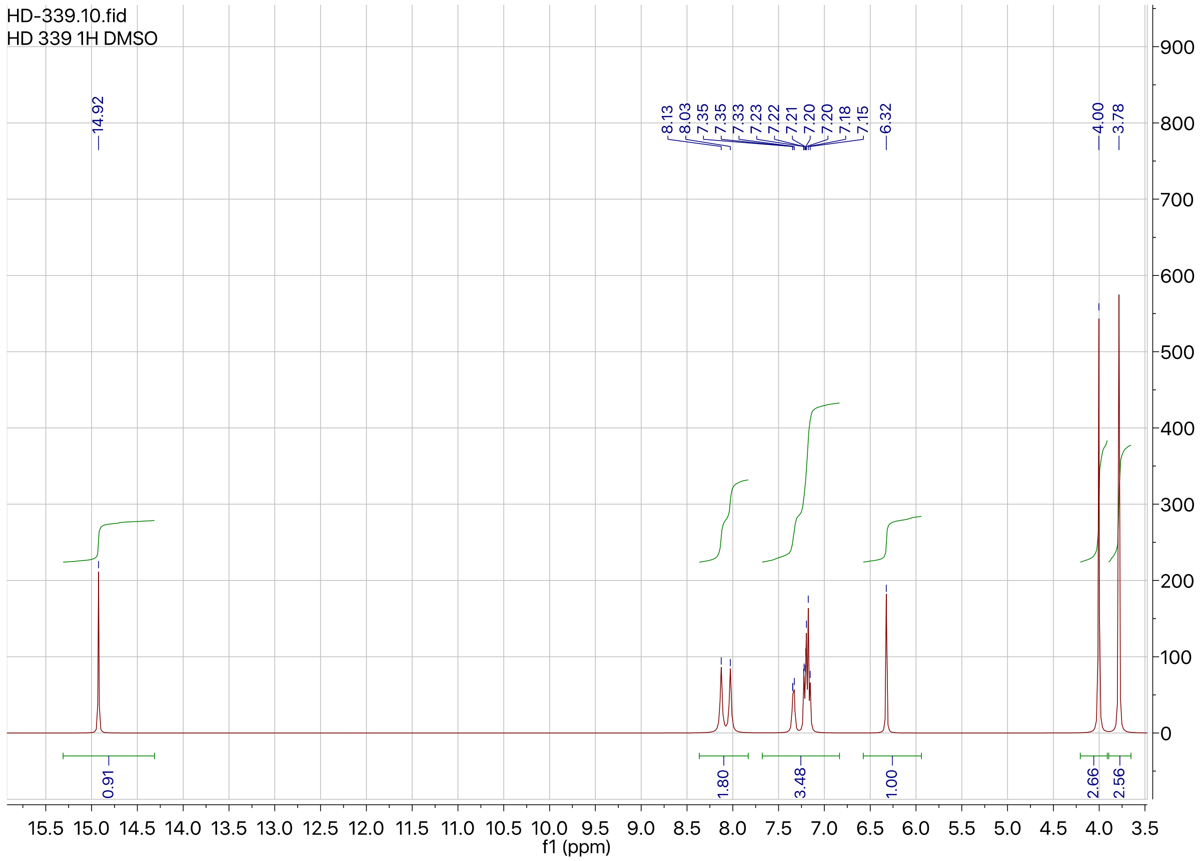

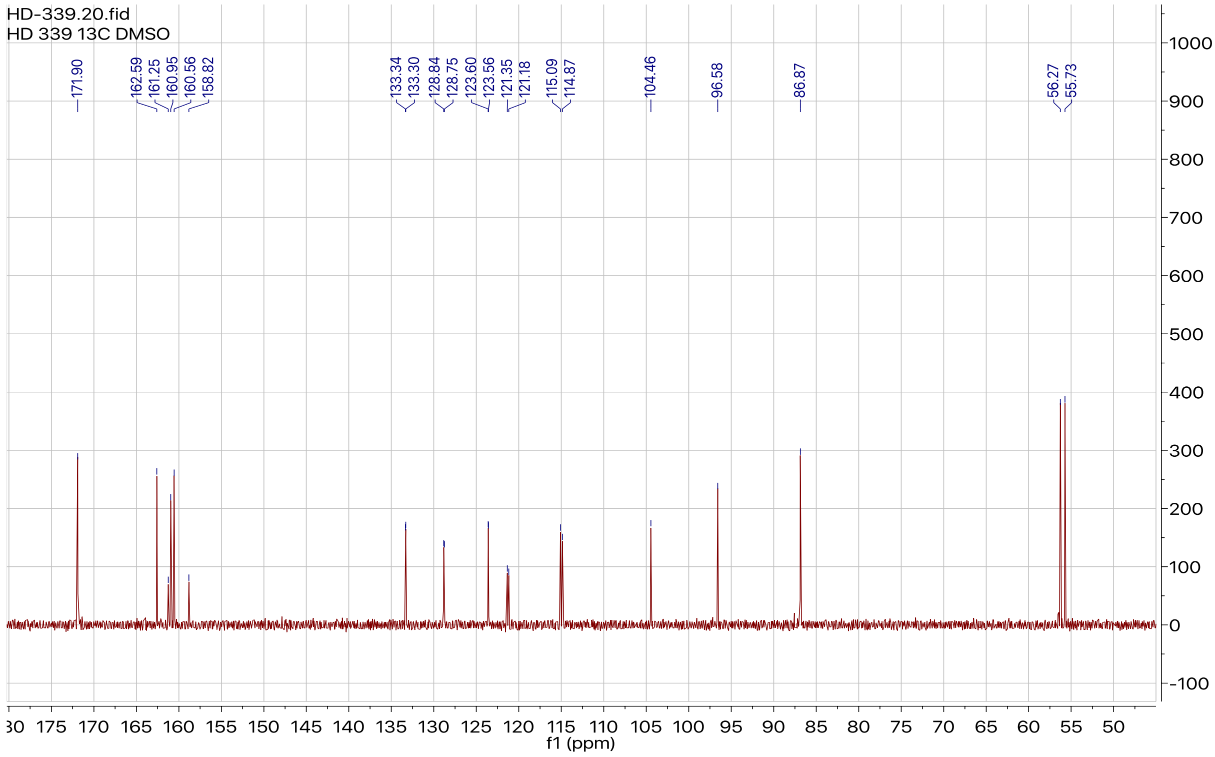

Supplement: Supplementary file 1 [file molecules-26-06572-s001.zip › molecules-1428883-supplementary.pdf]
